# An *in silico* approach to develop potential therapies against Middle East Respiratory Syndrome Coronavirus (MERS-CoV)

**DOI:** 10.1016/j.heliyon.2024.e25837

**Published:** 2024-02-09

**Authors:** Suvro Biswas, Mohasana Akter Mita, Shamima Afrose, Md. Robiul Hasan, Mst. Sharmin Sultana Shimu, Shahriar Zaman, Md. Abu Saleh

**Affiliations:** a Microbiology Laboratory, Department of Genetic Engineering and Biotechnology, University of Rajshahi, Rajshahi, 6205, Bangladesh; b Department of Genetic Engineering and Biotechnology, University of Rajshahi, Rajshahi, 6205, Bangladesh

**Keywords:** Phytoconstituents, MERS-CoV, 3CL^pro^, Molecular docking, ADMET, Molecular dynamics simulation

## Abstract

A deadly respiratory disease Middle East Respiratory Syndrome (MERS) is caused by a perilous virus known as MERS-CoV, which has a severe impact on human health. Currently, there is no approved vaccine, prophylaxis, or antiviral therapeutics for preventing MERS-CoV infection. Due to its inexorable and integral role in the maturation and replication of the MERS-CoV virus, the 3C-like protease is unavoidly a viable therapeutic target. In this study, 2369 phytoconstituents were enlisted from Japanese medicinal plants, and these compounds were screened against 3C-like protease to identify feasible inhibitors. The best three compounds were identified as Kihadanin B, Robustaflavone, and 3-beta-O- (*trans*-p-Coumaroyl) maslinic acid, with binding energies of −9.8, −9.4, and −9.2 kcal/mol, respectively. The top three potential candidates interacted with several active site residues in the targeted protein, including Cys145, Met168, Glu169, Ala171, and Gln192. The best three compounds were assessed by *in silico* technique to determine their drug-likeness properties, and they exhibited the least harmful features and the greatest drug-like qualities. Various descriptors, such as solvent-accessible surface area, root-mean-square fluctuation, root-mean-square deviation, hydrogen bond, and radius of gyration, validated the stability and firmness of the protein-ligand complexes throughout the 100ns molecular dynamics simulation. Moreover, the top three compounds exhibited better binding energy along with better stability and firmness than the inhibitor (Nafamostat), which was further confirmed by the binding free energy calculation. Therefore, this computational investigation could aid in the development of efficient therapeutics for life-threatening MERS-CoV infections.

## 1 Introduction

Middle East Respiratory Syndrome Coronavirus (MERS-CoV) is an unprecedented virus that caused Middle East respiratory syndrome (MERS), a highly deadly respiratory disease reported in June 2012 in the Arabian Peninsula and subsequently expanded globally [1]. It is the first lineage 2C Betacoronavirus with severe pathogenicity, imposing a dangerous impact on human health with a 35% fatality rate [2]. MERS-CoVs have several natural hosts, including *Camelus dromedarius* (dromedary camel), *Vespertilio superans* and *Neoromicia capensis* (two species of bats), and *Erinaceus europaeus* (European hedgehog). It is believed that MERS-CoV has originated from bats, and as human-to-bat transmission is limited, humans likely acquired this virus from the dromedary camels, acting as an intermediate host [3,4]. MERS-CoV belongs to the family Coronaviridae, sub-family Orthocoronavirinae, which includes four genera (Alpha, Beta, Gamma, and Delta) and the order Nidovirales. Six diverse coronaviruses (CoVs) have been identified as disease-causing agents in humans, with MERS causing strongly damaging and intensely severe respiratory tract infections [2,5]. MERS-CoV genome is a single-stranded positive-sense RNA with a size of about 30 kb. Phylogenetic analysis revealed two clades- A and B, with clade A having only few strains, while clade B contains most strains [2].

The length of the MERS-CoV genome is over 30,000 nucleotides (nt). The RNA genome of this virus has a 5′ untranslated region (UTR) with a length of 278 nt and a 3′ untranslated region with a length of 300 nt [6]. The first two-thirds of the 5′ UTR of the MERS-CoV genome comprise the replicase complex, which includes ORF1a and ORF1b. The remaining one-third of the 3′ UTR encodes four structural proteins: spike (S), membrane (M), envelope (E), and nucleocapsid (N), as well as accessory proteins (ORF3, ORF4a, ORF4b, ORF5, and ORF8b), which are not necessary for genome replication but are likely implicated in pathogenesis [7,8]. These accessory proteins of MERS-CoV do not share homology with any known virus or host [8]. Therefore, the order of the MERS-CoV genome is 5′ UTR-ORF1ab (Open reading frames)-S-ORF3-ORF4a-ORF4b-E-M-N-3′ UTR-poly(A) tail [7,9]. The spike (S) protein is located on the surface of beta-coronaviruses, playing a crucial role in transmission between different species by mediating receptor-virus recognition and activating the viral infection procedure [10]. The 5’ end of the MERS-CoV genome is translated to generate a large polyprotein, which is subsequently cleaved in cis into 16 functional nonstructural proteins by two viral proteases. These proteases collaborate in a complex process for viral RNA recombination and RNA synthesis [6].

In MERS-CoV, the S protein, containing 1353 amino acids, is a type I transmembrane glycoprotein comprising two subunits: S1 and S2. The S1 subunit contains the receptor binding domain (RBD), responsible for attaching to the target cell receptor and determining cellular tropism. On the other hand, the S2 subunit includes the fusion peptide (FP), a lengthy heptad repeats 1 domain (HR1), and a short heptad repeat 2 domains (HR2), which mediate membrane fusion [5,11,12]. A pivotal host cellular receptor for MERS-CoV is Dipeptidyl peptidase-4, also known as adenosine deaminase (ADA)-complexing protein-2 or CD26. It is an idiosyncratic 766-amino acid prominent type II transmembrane glycoprotein exposed as a homodimer on the cell surface, responsible for dipeptide cleavage [13,14]. The viral fleck of MERS-CoV enters the host cell by engaging with the dipeptidyl peptidase-4 (DPP4) cell receptor via the RBD in the spike protein on the host cell surface. Subsequently, the conformation of the viral virion particle is altered by the S2 subunit and embedded into the endosomal membrane or plasma membrane. Later, a fusion core, which is a six-helix bundle (6-HB), is formed by attaching HR2 with HR1, bringing the cell and viral membranes into close proximity for viral fusion [12,15,16]. MERS-CoV's genome consists of ORF1a and ORF1b, which produce two large polyproteins. The first, pp1a, contains 4382 amino acids, and the second, pp1ab, holds 7073 amino acids following a −1 ribosomal frameshift mechanism [17].

The 3C-like protease (3CL^pro^), also known as the main protease, M^pro^, or nsp5, is a dimer with a Cys-His dyad that can cleave polyproteins at the eleventh individual region, producing several non-structural proteins crucial in the viral replication process [18,19]. The M^pro^ is associated with two proteins, nsp4 and nsp6, and along with some parts of nsp3, forms the replication or transcription complex on the double-membrane vesicle originating from the endoplasmic reticulum membrane during infection. The 3CL^pro^ quaternary structure consists of each monomer formed by three structural domains. Among these domains, domain I and domain II form a catalytic cysteine prominent chymotrypsin-like architecture linked to the third C-terminal domain via an extended loop [[20], [21], [22]]. The M^pro^ cleavage substrate follows a typical pattern, such as (small)-X-(L/F/M)-Q↓(G/A/S)-X, where X denotes any amino acid, and represents the cleavage site. In the P1 position of the substrate, the glutamine (Q) residue is essential, while the P2 position strongly prefers a leucine residue [23,24]. Unlike the tightly associated dimer of SARS-CoV 3CL^pro^, a ligand is necessary to form a loosely associated dimer of MERS-CoV 3CL^pro^. In the presence of a ligand, MERS-CoV 3CL^pro^ structures exhibit a similar shape to SARS-CoV 3CL^pro^, with a backbone root-mean-square deviation (RMSD) of 1.06 Å over 232 Cα atoms in the protomers [25,26]. The 3C-like protease (3CL^pro^) has significant potential as an effective drug target against viral infections, given its auto-cleavage procedure catalyzed by an enzyme that is essential for viral replication and maturation [27].

Several studies have been conducted to develop an efficient strategy to halt MERS-CoV. Among them, an in vitro comparative study was undertaken for the treatment of Ribavirin, Chloroquine, Toremifene, and Chlorpromazine [28,29]. Additionally, numerous comparative studies have been carried out on rhesus monkeys, Ad5-hCD26-transduced mice, and twelve healthy common marmosets [30,31]. Retrospective cohort and case report studies have also been performed to explore potential antiviral therapies [31]. To date, no vaccines, antiviral therapeutics, or prophylaxis have been approved for preventing or treating MERS-CoV infections. Despite this, numerous therapeutics have been demonstrated in preclinical studies, and several of them have undergone clinical testing [32,33]. Several potential vaccines are currently undergoing clinical and preclinical trials. MVA-MERS-S and ChAdOx1 MERS vaccines are in phase I clinical trials [34,35]. Additionally, MVvac2-MERS-N, RLP3-GEM, rNTD, VSVΔG-MERS, pcDNA3.1-S1, and rAd/Spike vaccines are in the preclinical stages [[36], [37], [38], [39]]. This investigation aims to identify potential inhibitors against the 3CL^pro^ of MERS-CoV by screening 2369 phytoconstituents obtained from Japanese medicinal plants, using an extensive array of *in silico* techniques.

## 2 Materials and methods

### 2.1 Protein preparation

The 3D (three-dimensional) crystal structure (PDB ID: 5WKK; Method: X-ray diffraction; Resolution: 1.55 Å; Organism: MERS) of the MERS 3CL protease was obtained from the Protein Data Bank (PDB) database maintained by RCSB (Research Collaboratory for Structural Bioinformatics) [40]. Pymol [41], in conjunction with Discovery Studio software [42], was used to prepare the selected protein structure initially by excluding heteroatoms along with water molecules. YASARA tools [43] were employed to perform the energy-minimization process on the prepared clean protein structure to obtain a validated, optimized, and energy-minimized protein. The minimized protein structure was saved for executing additional molecular docking and molecular dynamics (MD) simulation schemes.

### 2.2 Ligand preparation

About 2369 phytochemical constituents extracted from Japanese medicinal plants were listed after an extensive literature search (Supplementary files 1–5). Google Scholar (https://scholar.google.com/), ResearchGate (https://www.researchgate.net/), PubMed (https://pubmed.ncbi.nlm.nih.gov/), Dr. Duke's Phytochemical and Ethnobotanical database (https://phytochem.nal.usda.gov/), along with various web tools, were utilized for phytochemical screening. Gas chromatography-mass spectroscopy, in conjunction with liquid chromatography-mass spectroscopy data, was considered for additional validation of the phytochemical dataset. Furthermore, the phytochemical dataset was rechecked to ensure accuracy, and duplicate entries were removed. The 3D structure of the listed phytochemicals was retrieved from the PubChem database [44]. The phytochemicals identified as potential ligands were optimized through a cleaning and energy minimization process using the mmff94 force field [45], with approximately 2000 minimization steps and the sheared gradient optimization algorithm. Additionally, a dataset of MERS-CoV inhibitors (10 inhibitors) was prepared based on literature studies [[46], [47], [48], [49]].

### 2.3 Active site prediction

To predict the active sites, we utilized the PDBsum database (https://www.ebi.ac.uk/pdbsum/) and the Protein-Ligand Interaction Profiler (PLIP) database (https://plip-tool.biotec.tu-dresden.de/plip-web/plip/index). These databases offer a pictorial overview of each 3D structure available in the Protein Data Bank (PDB). Consequently, we conducted a search in the database using the PDB code (5WKK) to retrieve the active sites of the 3CL^pro^ protein. The database provides schematic diagrams that accurately predict the functional and active sites of the protein [50].

### 2.4 Molecular docking study

The computer-based screening approach, molecular docking, was implemented to estimate the systematic binding affinity and interaction mode between the listed phytochemicals and the MERS 3CL protease, utilizing the 'Autodock Vina' program [51]. The structural optimization of the phytochemical-based ligands was completed using the Universal Force Field (Uff). After conversion into PDBQT format, the phytochemical-based ligands were made accessible to Autodock for executing the docking method, with unstable bonds being functionally eliminated. A grid box with center points specified as X = −26.99, Y = 12.6039, Z = 58.9455, and dimensions noted as X = 50.3334, Y = 67.2744, Z = 59.2586 (each in Angstrom) was created after ligand and protein preparation using Autodock Vina. In the case of the 3CL protease, the cocrystallized fixed protein structure obtained as (PDB: 5WKK) was prepared in a similar protocol for convenient conversion in Autodock scheme for the docking method. Thus, the molecular docking study between the ligand and protein is implemented as a control system in the docked research. Binding affinities of the ligands were assessed in kcal/mol based on negative values, where higher negativity indicates more competent binding. MERS-CoV inhibitors (10 inhibitors) were also used for docking with the 3CL^pro^ protein.

### 2.5 ADMET

Several reliable online servers, including admetSAR [52], SwissADME [53], and pKCSM [54], were employed to assess the pharmacokinetic features based on ADMET predictions. These predictions encompass distribution, excretion, absorption, metabolism, and toxicity. The Canonical SMILES (simplified molecular-input line-entry system) for the screened plausible antiviral phytochemicals were retrieved from the PubChem database. These Canonical SMILES, which lack stereochemical and isotopic information and are derived from chemical structures, were used as input for the mentioned web servers to obtain ADMET predictions that can reveal the drug-likeness features of the phytochemicals.

### 2.6 Molecular dynamics simulations

The molecular dynamics simulation study was conducted using the YASARA dynamics software package [43], assisted by the AMBER14 force field [55]. The docked complexes were initially cleaned, optimized, and hydrogen bond networks were oriented. The TIP3P water solvation model was applied with periodic boundary conditions [56]. The simulations were conducted under physiological conditions set at 298K, pH 7.4, and 0.9% NaCl. Initial energy minimizations were performed using the steepest gradient algorithms with the simulated annealing method (5000 cycles). Long-range electrostatic interactions were calculated using the Particle Mesh Ewald (PME) methods [[57], [58], [59]]. The time step for the simulations was set to 2.0fs, and trajectory snapshots were saved every 100ps. The simulations were extended for 100ns following constant pressure and Berendsen thermostat protocols [60]. The simulation trajectories were utilized to calculate the root mean square deviations (RMSD), root mean square fluctuations (RMSF), solvent accessible surface area (SASA), radius of gyrations (Rg), and hydrogen bonds [[61], [62], [63], [64], [65], [66], [67], [68], [69], [70]]. Additionally, the Molecular Mechanics-Poisson-Boltzmann Surface Area (MM-PBSA) approach, along with the AMBER14 force field, was employed in the YASARA dynamics software package to compute the binding free energy [71]. For the computation of binding free energy, the default macro file was modified, and the following equation was used:

ΔGbind = ΔGcomplex_(minimised)_ – [ΔGligand_(minimised)_ + ΔGreceptor_(minimised)]_

ΔGbind = ΔG_MM_ + ΔG_PB_ + ΔG_SA_ − TΔS

**Fig. 1:**
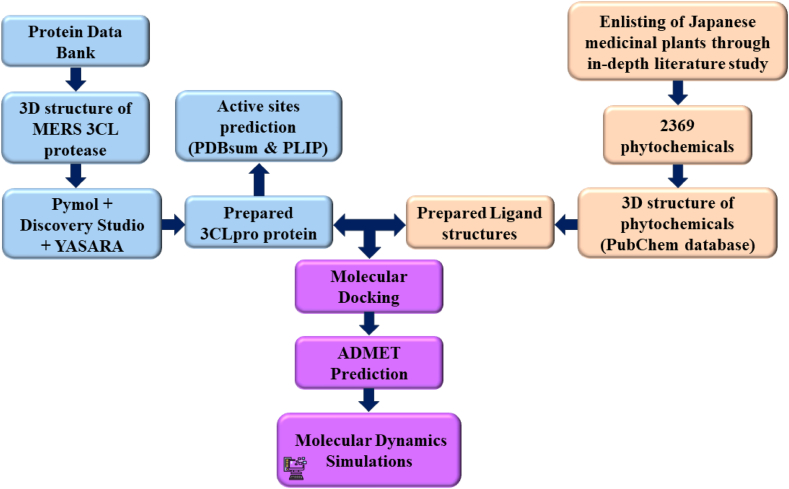
Flow chart showing the main steps of the study.

Here, ΔG_MM_ denotes the sum of electrostatic and van der Waals interactions; ΔG_SA_ and ΔG_PB_ indicate the non-polar and polar solvation energies, respectively; and TΔS denotes the entropic contribution [72]. A flow chart showing the main steps of the study is depicted in Fig. 1.

## 3 Results

### 3.1 Active site prediction

**Fig. 2:**
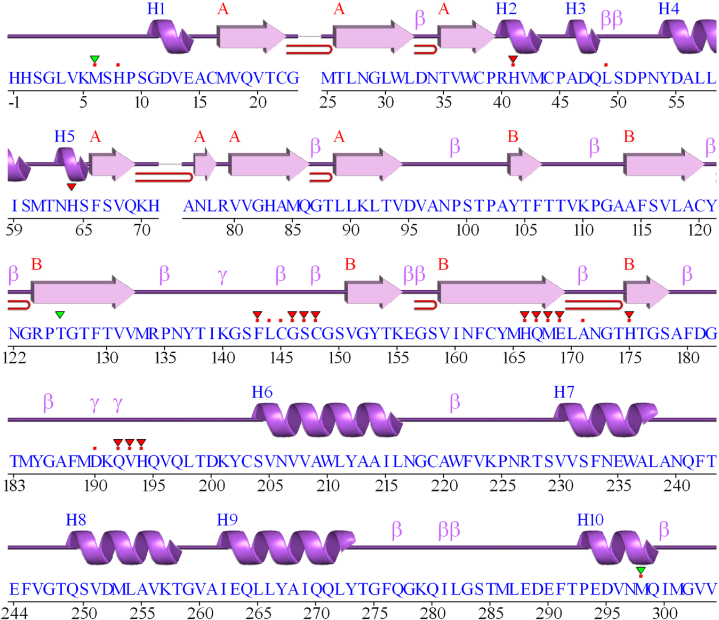
The red dots indicate the active site of the MERS-CoV 3CL^pro^ protein which was retrieved from the PDBsum database. (For interpretation of the references to colour in this figure legend, the reader is referred to the Web version of this article.)

From the PDBsum database, the active groove of the 3CL^pro^ from MERS-CoV was identified. The red dots in Fig. 2, retrieved from the PDBsum database, indicate the active site of the 3CL^pro^ protein. A total of twenty-one active sites were determined by the PDBsum database, including Met6, His8, His41, Leu49, Phe143, Leu144, Cys145, Gly146, Ser147, Cys148, His166, Gln167, Met168, Glu169, Ala171, His175, Asp190, Qln192, Val193, His194, and Met298. The active site residues obtained from the PDBsum database encompass all the active site residues found in a previous study [73].

**Fig. 3:**
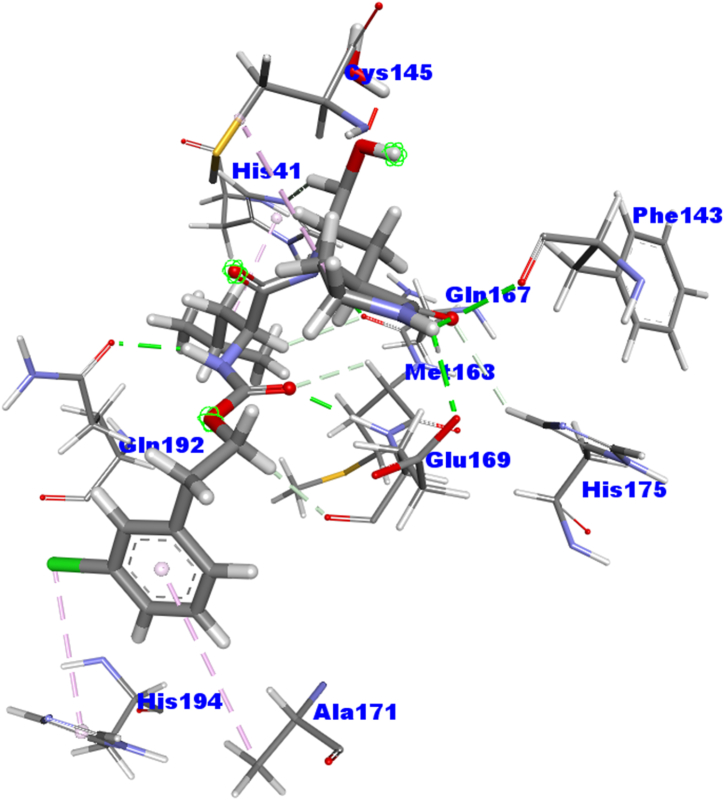
The active sites of the MERS-CoV 3CL^pro^ protein were retrieved from the PLIP database.

On the contrary, a total of ten active sites were determined (Fig. 3) by the PLIP database: His41, Phe143, Cys145, Gln167, Met168, Glu169, Ala171, His175, Gln192, and His194. Therefore, ten amino acid residues are considered active sites of the 3CL^pro^ protein for this study, based on the amino acid residues obtained from both databases (PDBsum & PLIP). To evaluate the non-bonding interactions from the molecular docking study, these ten amino acid residues from the 3CL^pro^ were carefully considered.

### 3.2 Molecular docking analysis

**Fig. 4:**
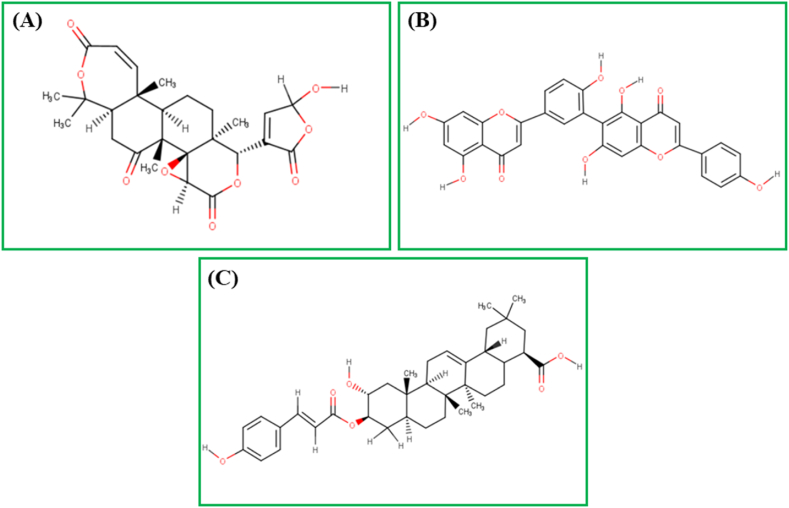
Two-dimensional (2D) chemical structures of (A) Kihadanin B, (B) Robustaflavone, and (C) 3-beta-O-(*trans*-p-Coumaroyl)maslinic acid. The structures were drawn using MarvinSketch software.

A total of 2369 phytochemicals were docked with the 3CL^pro^ protein, with 25 being selected as the top binding molecules (Supplementary File 6; Supplementary Table 1). Re-docking was performed to ensure accuracy and avoid errors after selecting the top 25 molecules with higher binding affinities (Supplementary File 6; Supplementary Table 2). After redocking the top 25 compounds, the top 10 phytochemicals were selected based on docking energy (Supplementary File 6; Supplementary Table 3), and finally, the top three **[**Fig. 4(A–C)**]** compounds were chosen for further study.

**Fig. 5:**
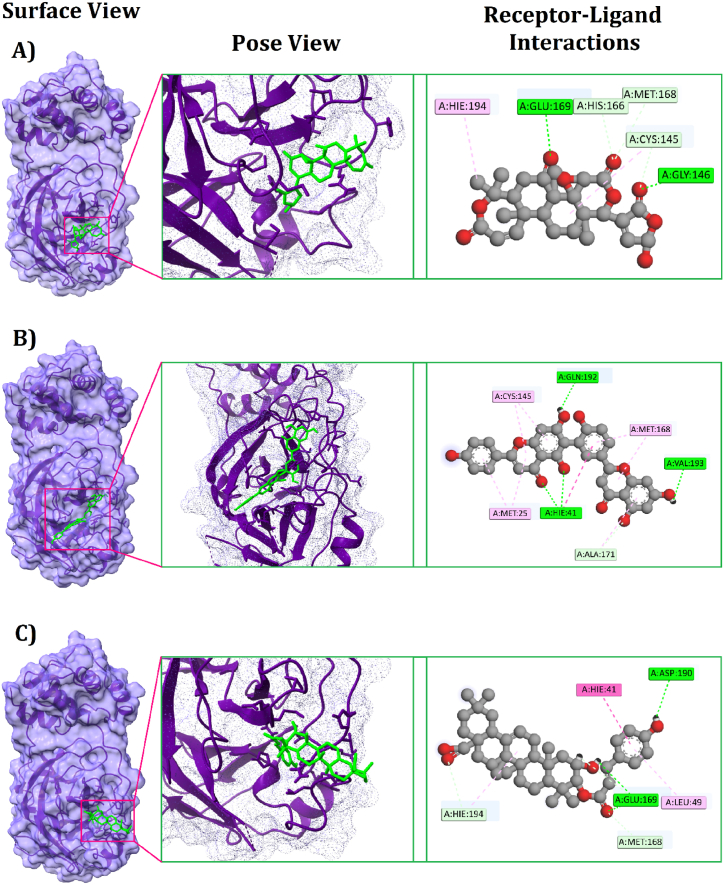
Different binding modes for the selected compounds within the MERS-CoV 3CL^pro^ protein's active and catalytic sites; (A) Kihadanin B, (B) Robustaflavone, (C) 3-beta-O-(*trans*-p-Coumaroyl)maslinic acid.

**Fig. 6:**
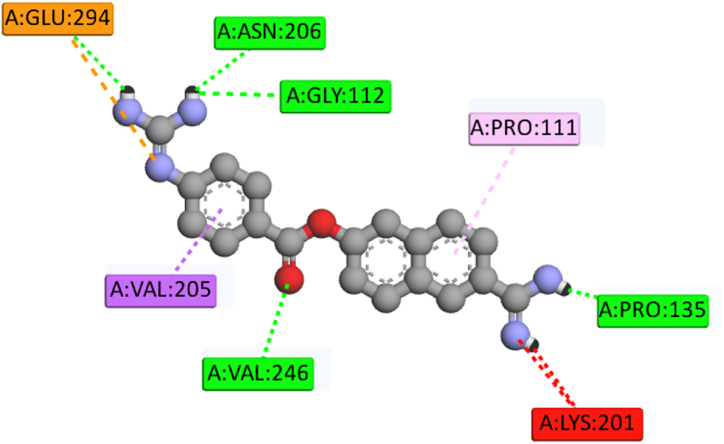
Two-dimensional (2D) view of the interactions between MERS-CoV 3CL^pro^ and the top inhibitor (Nafamostat).

The selected 10 inhibitors were also docked against the MERS-CoV 3CL^pro^ protein **(**Table 2**)**. Nafamostat exhibited the highest binding affinity as −8.1 kcal/mol and for this reason, only Nafamostat is selected and used for further studies. Nafamostat expressed eight interactions with 3CL^pro^ having one Electrostatic Bond at GLU294 position, four Conventional Hydrogen Bonds at PRO135, GLY112, ASN206, VAL246 positions, one Pi-Sigma Bond at VAL205 position, one Pi-Alkyl Bond PRO111 position, and one Unfavorable Bond at LYS201 position (Table 3, Fig. 6).

**Table 1:** Non-bond interactions between the top three compounds (selected based on docking scores) and the MERS-CoV 3CL^pro^ protein.

Compounds	PubChem CID	Binding Affinity (kcal/mol)	Residues in contact	Interaction type	Distance in Å
Kihadanin B	156766	−9.8	GLU169	Conventional Hydrogen Bond	1.93232
			MET168	Carbon Hydrogen Bond	2.76511
			HIE194	Pi-Alkyl Bond (Hydrophobic)	4.00511
			HIS166	Carbon Hydrogen Bond	2.44893
			CYS145	Carbon Hydrogen Bond	2.79336
			GLY146	Conventional Hydrogen Bond	1.76957
Robustaflavone	5281694	−9.4	HIE41	Conventional Hydrogen Bond	2.1817
			GLN192	Conventional Hydrogen Bond	1.68389
			VAL193	Conventional Hydrogen Bond	1.74401
			ALA171	Carbon Hydrogen Bond	2.66935
			CYS145	Pi-Alkyl Bond (Hydrophobic)	5.11343
			MET25	Pi-Alkyl Bond (Hydrophobic)	4.63918
			MET168	Pi-Alkyl Bond (Hydrophobic)	5.42348
3-beta-O-(*trans*-p-Coumaroyl) maslinic acid	14335962	−9.2	GLU169	Conventional Hydrogen Bond	1.99887
			ASP190	Conventional Hydrogen Bond	2.78672
			MET168	Carbon Hydrogen Bond	2.47132
			HIE194	Carbon Hydrogen Bond	3.09155
			HIE41	Pi-Pi Stacked Bond (Hydrophobic)	4.14442
			LEU49	Pi-Alkyl Bond (Hydrophobic)	4.97781

With absolute ensuring on proper running in the analyzing tool tilled as ‘Autodock vina’, the top three ‘Kihadanin B’, ‘Robustaflavone’, and ‘3-beta-O-(*trans*-p-Coumaroyl)maslinic acid’ showed the binding affinity as −9.8, −9.4, and −9.2 kcal/mol respectively. On the contrary, ‘BIOVIA Discovery Studio’ explored the non-binding interaction between these three drug candidates and the 3C-like protease (3CL^pro^). Preliminary, the first drug candidate, Kihadanin B expressed six interactions with 3CL^pro^ where one Pi-Alkyl bond at HIE194, two Conventional Hydrogen bonds at GLU169, and GLY146, and three Carbon Hydrogen bonds at MET168, HIS166, and CYS145 was observed. Robustaflavone also provided seven interacting sites where one was at ALA171 with Carbon Hydrogen bond, three Conventional Hydrogen bonds at HIE41, GLN192, VAL193 positions, and three Pi-Alkyl bonds at CYS145, MET25, MET168 positions. On the other hand, the last drug candidates showed complex interaction having one Pi-Pi Stacked bond at the HIE41 residue position, one Pi-Alkyl bond at the LEU49 residue position, and two Conventional Hydrogen bonds at GLU169 and ASP190 residue position, and two Carbon Hydrogen bonds at MET168 and HIE194 residue position **(**Table 1**)**, **[**Fig. 5(A–C)**].**

**Table 2:** Binding Affinities of the selected 10 inhibitors against MERS-CoV 3CL^pro^ protein.

Inhibitor Names	PubChem CID	Binding Affinity (kcal/mol)
Nafamostat	4413	−8.1
Nelfinavir	64143	−7.8
Camostat	2536	−7
Resveratrol	445154	−6.7
Nitazoxanide	41684	−6.4
Ribavirin	37542	−6.3
Alisporivir	11513676	−6.1
Cyclosporin A	5284373	−6
Ritonavir	392622	−5.9
Mycophenolate	446541	−5.7

**Table 3:** Non-bond interactions between MERS-CoV 3CL^pro^ and the top inhibitor (Nafamostat).

Inhibitors	PubChem CID	Binding Affinity (kcal/mol)	Residues in contact	Interaction type	Distance in Å
Nafamostat	4413	−8.1	GLU294	Electrostatic Bond	4.73878
			PRO135	Conventional Hydrogen Bond	2.19994
			GLY112	Conventional Hydrogen Bond	2.72113
			ASN206	Conventional Hydrogen Bond	2.5218
			VAL246	Conventional Hydrogen Bond	2.23728
			VAL205	Pi-Sigma Bond	2.38199
			PRO111	Pi-Alkyl Bond	4.90979
			LYS201	Unfavorable Bond	3.82713

### 3.3 ADMET

The drug-likeness properties of the three selected compounds were evaluated using ADMET calculations (Table 4). According to Lipinski's rule of 5, which indicates drug-likeness, the standard values for molecular weight, hydrogen bond donors, and hydrogen bond acceptors should be less than 500 g/mol, less than 5, and less than 10, respectively [74]. Kihadanin B (486.51 g/mol) with 1 hydrogen bond donor and 9 hydrogen bond acceptors, Robustaflavone (538.46 g/mol) with 6 hydrogen bond donors and 10 hydrogen bond acceptors, and 3-beta-O-(*trans*-pCoumaroyl) maslinic acid (618.84 g/mol) with 3 hydrogen bond donors and 6 hydrogen bond acceptors meet the drug-likeness criteria. It is worth noting that natural compounds, especially, may accept up to 2 violations of the Lipinski rule [74]. In a recent analysis, the highest mean TPSA value was calculated to be 108 Å^2^ (95th percentile: 202 Å^2^), and the TPSA values for Kihadanin B (128.73 Å^2^), Robustaflavone (181.80 Å^2^), and 3-beta-O-(*trans*-pCoumaroyl) maslinic acid (104.06 Å^2^) are below the 95th percentile value. Concerning human intestinal absorption, the mean experimental value for drugs is 75.97% [75]. Therefore, Kihadanin B (100%), Robustaflavone (90.473%), and 3-beta-O-(*trans*-p-Coumaroyl) maslinic acid (78.769%) exhibit satisfactory human intestinal absorption. Toxicity, which indicates potential damage to cells and organs, is a crucial parameter for drug development [76]. As all of the compounds are noncarcinogens, they exhibit no AMES toxicity, hepatotoxicity, and skin sensitization [77,78].

**Table 4:** Pharmaceutical profiles derived from the SwissADME, admetSAR, and pKCSM webservers for the top three potential candidates obtained from docking.

Parameters	Kihadanin B	Robustaflavone	3-beta-O-(*trans*-p-Coumaroyl)maslinic acid
Molecular Weight	486.51 g/mol	538.46 g/mol	618.84 g/mol
Num. H-bond acceptors	9	10	6
Num. H-bond donors	1	6	3
TPSA (S)	128.73 Å^2^	181.80 Å^2^	104.06 Å^2^
Human Intestinal Absorption	100%	90.473%	78.769%
AMES Toxicity	No	No	No
Hepatotoxicity	No	No	No
Skin sensitization	No	No	No
Carcinogens	Non-carcinogens	Non-carcinogens	Non-carcinogens

### 3.4 Molecular dynamics simulation

**Fig. 7:**
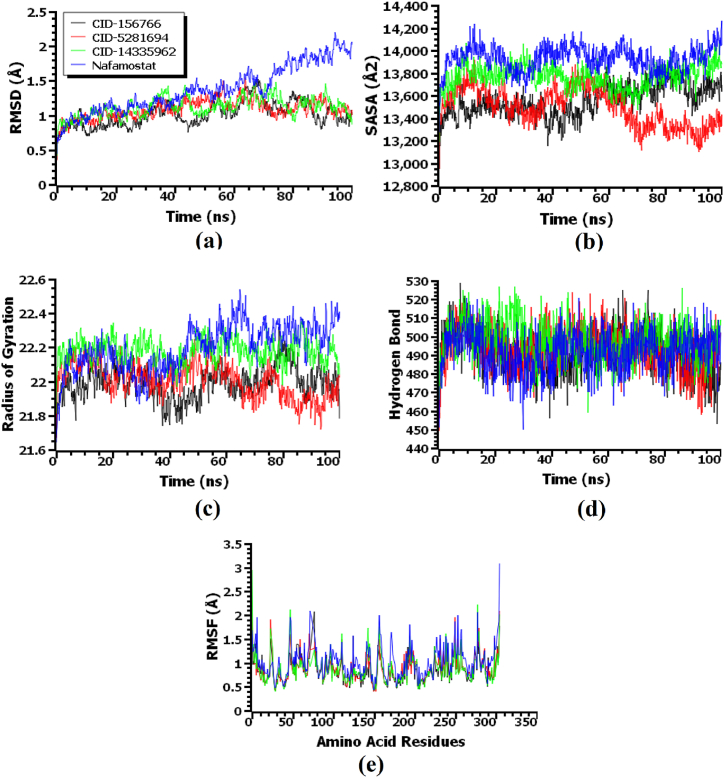
The molecular dynamics simulation of the top three compound-MERS-CoV 3CL^pro^ complexes along with Nafamostat-MERS-CoV 3CL^pro^ complex, here (a) root mean square deviation of the c-alpha atoms, (b) solvent accessible surface area, (c) radius of gyration, (d) hydrogen bonding of the complexes, (e) root mean square fluctuation of the complexes.

The molecular dynamics simulation study aimed to understand the structural stability of the docked complexes. The root mean square deviations (RMSD) of the C-alpha atoms of the docked complexes were examined to illustrate the binding stability. Fig. 7 (a) indicates that the CID-156766 (Kihadanin B)-3CL^pro^, CID-5281694 (Robustaflavone)-3CL^pro^, CID-14335962 (3-beta-O-(*trans*-p-Coumaroyl) maslinic acid)-3CL^pro^, and Nafamostat-3CL^pro^ complexes initially exhibited an upper trend in RMSD, indicating the flexible nature of the complexes at the beginning phase. However, the top three complexes began to stabilize after 25ns and maintained stability for the rest of the simulation periods with minor fluctuations. In contrast, the Nafamostat-3CL^pro^ complex showed increased flexibility in the final 30 ns of the simulation time. Overall, the RMSD of the complexes remained below 2.5 Å, defining the stable nature of the complexes [64].

Furthermore, the solvent accessible surface area (SASA) of the complexes was analyzed to understand changes in the surface area, where higher SASA defines the extensions of the surface area, and a lower SASA value relates to the truncated nature of the complexes [68]. Fig. 7 (b) indicates that the CID-14335962 (3-beta-O-(*trans*-p-Coumaroyl) maslinic acid)-3CL^pro^ complex had extensions in the surface upon the binding of the ligand molecules, whereas the other two complexes had a stable SASA profile. The higher SASA values of the Nafamostat-3CL^pro^ complex signify the expansion of the protein surface area. The radius of gyrations profile of the simulated complexes was also analyzed, where higher Rg relates to the mobile nature of the complexes, and lower Rg relates to the stable nature of the complexes [65]. Fig. 7 (c) indicates that the CID-14335962 (3-beta-O-(*trans*-p-Coumaroyl) maslinic acid)-3CL^pro^ complex exhibited a higher Rg and more flexibility, whereas the other two complexes demonstrated the rigid nature of the complexes. Furthermore, compared to the top three complexes, the Nafamostat-3CL^pro^ complex showed a higher average Rg value, indicating its greater flexibility.

**Fig. 8:**
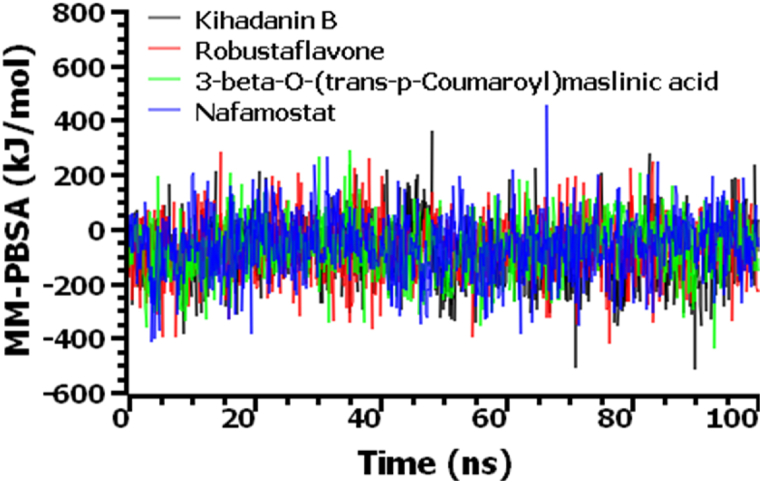
The binding-free energy of the top three drugs and inhibitor complexes calculated by the MM-PBSA method.

The hydrogen bonds in the simulation complexes play a crucial role in determining stability in macromolecular systems [79]. All four complexes formed a substantial quantity of hydrogen bonds, which were essential for the integrity and stability of the complexes (see Fig. 7d). Root mean square fluctuations (RMSF) define the flexibility across the amino acid residues. Fig. 7e indicates that most residues had RMSF values lower than 2.5 Å, with minor fluctuations, signifying the stability of the complexes [68]. Moreover, the CID-156766 (Kihadanin B)-3CL^pro^, CID-5281694 (Robustaflavone)-3CL^pro^, CID-14335962 (3-beta-O-(*trans*-p-Coumaroyl)maslinic acid)-3CL^pro^, and Nafamostat-3CL^pro^ complexes exhibited average binding free energies of −86.54 kJ/mol, −73.82 kJ/mol, −67.08 kJ/mol, and −61.79 kJ/mol, respectively (see Fig. 8). In the MM-PBSA computation, the top three drug complexes exhibited higher average binding free energy, suggesting more efficient binding than the inhibitor (Nafamostat).

## 4 Discussion

Since the detection of MERS-CoV in 2012, the ominous fatality rate of 35% and transmission via close contact with an R0 (the basic reproduction number) varying from 0.8 to 1.3 in the scarcity of infection control measures have raised concerns about a potential global pandemic, especially during the Hajj Pilgrimage [80,81]. Additionally, available data-derived evidence of adaptive evolution in MERS-CoV-related viruses via the heptad repeat region, along with in vitro-based confirmation for boosting MERS-CoV infection efficacy through mutations like T1015 N, has underscored the necessity for designing antiviral therapy against MERS-CoV [82]. However, currently, there is neither an approved vaccine for preventing MERS-CoV infection nor any specific treatment for Middle East respiratory syndrome caused by MERS-CoV [83]. Therefore, computer-based virtual screening through molecular docking and molecular dynamics simulation can be a dependable approach to uncover plausible inhibitors or repurposable drugs against MERS-CoV, as the three-dimensional crystal structure of the MERS 3CL protease is available to reveal receptor−ligand interactions with elaborated investigations.

According to the crystal structure of 3CL^pro^, each monomer remains perpendicular in the homodimer consisting of a core chymotrypsin-like fold assembled by two domains, designated domains I and II, consisting of residues 1–187, a prolonged connecting loop consisting of residues 188–204, and domain III or C-terminal alpha-helical domain consisting of residues 205–306 [21,84]. In the case of the proteolytic site, glutamine, leucine, basic residues, and small hydrophobic residues at P1, P2, P3, and P4 positions, respectively, are preferred by the 3CL^pro^. Additionally, small residues are required at P1′ and P2′ positions [21,85]. As 3CL^pro^ is responsible for cleavage at 11 sites during the production of 16 nonstructural proteins, it holds significance in the autocleavage process during the proteolytical process of the polyproteins named pp1a and pp1ab. Thus, it is required for viral replication and maturation to conduct the coronaviral life cycle, indicating it to be a reliable target for inhibition and making it suitable as a substrate against plausible phytochemicals or repurposable drugs [86,87].

In this investigation, approximately 2369 phytochemical components extracted from Japanese medicinal plants were utilized as data to be operated as ligands. The 3D or three-dimensional crystal structure of the MERS 3CL protease had been retrieved from the PDB databank to be used as the protein target for MERS CoV inhibition. Later, after the protein and ligand preparation, a molecular docking approach was executed to assess the systematic binding affinity, along with the interaction mode between the MERS 3CL protease and enlisted phytochemicals for prompt recognition of potential inhibitors within a short period. Based on binding affinity, the top 25 phytochemicals were re-docked, among which the top 3 compounds were determined as Kihadanin B, Robustaflavone, and 3-beta-O-(*trans*-p-Coumaroyl)maslinic acid with binding energies of −9.8, −9.4, and −9.2 kcal/mol, respectively, for further investigation. Besides, Nafamostat exhibited the highest binding affinity at −8.1 kcal/mol among the selected 10 inhibitors of MERS CoV.

Kihadanin B, a limonoid extracted from the immature peel of Citrus unshiu, Araliopsis soyauxii Engl. plant, Phellodendron amurense bark, and Dictamnus dasycarpus's root bark, has demonstrated various medicinal properties. These include suppressing adipogenesis through the repression of the Akt-FOXO1-PPARγ axis [88], significant cytotoxicity against nine tested cancer cell lines [89], notable antifeedant activity tested against Reticulitermes speratus [90], and cytotoxicity against human cancer cell lines [91]. Robustaflavone, a biflavonoid extracted from N. domestica fruits, Garcinia latissima Miq. leaves, and Rhus succedanea, exhibits several activities, including inflammation inhibition [92], antibacterial activity [93], and potential inhibition of hepatitis B virus replication [94]. 3-beta-O-(*trans*-p-Coumaroyl)maslinic acid, a strictinin isomer isolated from the root of Rosa roxburghii Tratt (Ci Li Gen), demonstrates an antimicrobial mechanism [95].

Then, ADMET predictions for the identified phytochemical hits were conducted using multiple online-based servers to ensure drug-likeness features based on pharmacokinetic estimations. Subsequently, molecular dynamics simulations were executed to confirm binding rigidity and stability, relying on multiple descriptors of the simulation trajectories. A more stable binding was observed for the three hit phytochemicals compared to the inhibitor (Nafamostat), which was further confirmed by the calculation of binding free energy. nHowever, the active site for the 3CL^pro^ from MERS-CoV was obtained from the PDBsum database, which mentioned twenty-one active sites. Among the three hit phytochemicals, Kihadanin B binds to 3CL^pro^ through six non-bond interactions, three of which involve the residues Glu169, Met168, and Cys145 located in the active site of the 3CL^pro^ of MERS-CoV. Robustaflavone is bound by seven non-bond interactions, four of which involve the residues Gln192, Ala171, Cys145, and Met168, located in the 3CL^pro^ active site. Similarly, 3-beta-O-(*trans*-p-Coumaroyl) maslinic acid binds with 3CL^pro^ via six non-bond interactions, two of which involve the residues Glu169 and Met168, situated in the active site of the MERS-CoV 3CL^pro^. As several amino acid residues involved in the non-bond interactions are located within the active site, the three top phytochemicals can be considered for potential use as a drug for antiviral therapy concerning MERS-CoV.

An investigation of docked compounds from the ZINC database against the 3CL^pro^ protein of MERS-CoV uncovered three hit compounds, including ZINC ID: 75121653, 41131653, and 67266079, possessing binding energies of −7.12, −7.1, and −7.08 kcal/mol, respectively [96]. In another prior study, compounds available in the National Cancer Institute (NCI) database were docked against the MERS-CoV 3CL^pro^ protein. They identified five hit compounds, including NSC648199, NSC159375, NSC29007, NSC335985, and NSC337571, with binding energies of −8.7, −8.3, −8.1, −8.1, and −7.8 kcal/mol, respectively. These values were lower than the binding energies uncovered in our current study, which found the top three compounds Kihadanin B, Robustaflavone, and 3-beta-O-(*trans*-p-Coumaroyl)maslinic acid with binding affinities of −9.8, −9.4, and −9.2 kcal/mol, respectively [97].

In another previous study, several flavonoids utilized as MERS-CoV 3C-like protease inhibitors showed favorable binding affinity. However, among them, Helichrysetin, Herbacetin, and isobavachalcone did not bind with the residues of the active site. Only Quercetin 3-β-D-glucoside was found to bind with Gln 169 residues at the active site [21]. In a different analysis, two potent and permeable inhibitors were synthesized through structure-based design to inhibit MERS Coronavirus 3C-like protease. Among them, 10a bound with one residue (Cys148), and 10c bound with five residues at the active site (His41, Phe143, Gln192, Gln167, and Glu169) [98]. Contrastingly, in our findings, all the hit compounds interacted with the amino acid residues at the active site. For instance, Kihadanin B interacted with Glu169, Met168, and Cys145; Robustaflavone interacted with Gln192, Ala171, Met168, and Cys145; and 3-beta-O-(*trans*-p-Coumaroyl) maslinic acid interacted with Glu169 and Met168. Thus, our hit compounds have more potential to block the active site, inhibiting the function of the 3C-like protease, which is crucial for viral protein maturation and replication.

In addition, plant-derived phytochemicals, including Kaempferol (−9.3 kcal/mol), Resveratrol (−9.31 kcal/mol), Quercetin (−11.88 kcal/mol), and Theaflavin (−14.35 kcal/mol), showed the highest binding affinity for the 3CL^pro^ protein of MERS-CoV. However, the relevant study lacks an analysis of the binding mode stability of the docked complex through molecular docking simulation [99]. Conversely, all the hit compounds in our study, including Kihadanin B, Robustaflavone, and 3-beta-O-(*trans*-p-Coumaroyl) maslinic acid, exhibited the structural stability and firmness of the docked complexes over 100 ns of molecular dynamics simulation. After a thorough literature review, it is noted that Kihadanin B exhibits suppression of adipogenesis by reducing lipid accumulation and suppressing the Akt-FOXO1-PPARγ axis in 3T3-L1 adipocytes [88]. Likewise, Robustaflavone manifests antibacterial activity, anti-inflammatory activity, potent inhibition of hepatitis B virus replication in the 2.2.15 cell line, anti-angiogenic and pro-apoptotic impacts, cytotoxic effect on cancer cells, and antioxidant effect [100]. Similarly, 3-beta-O-(*trans*-p-Coumaroyl) maslinic acid is found to be associated with an antimicrobial mechanism [95].

The top three phytoconstituents evaluated in this study may be able to prevent the 3CL^pro^ of MERS-CoV from performing its function, as determined by a combination of computational techniques, including molecular docking and molecular dynamics investigations. These three plausible candidates exhibited higher binding affinity, showed interaction with the active sites of the protein, displayed lower toxic characteristics with maximum drug-likeness properties, and also exhibited the structural stability and firmness of the docked complexes over 100 ns of simulation time. Therefore, these three phytochemicals can be potential candidates as antiviral therapeutics for combating MERS-CoV. Besides, the creation of these new phytoconstituents datasets will allow researchers to carry out investigations against other specific viral and bacterial proteins or other disease-causing molecules. Despite the verification of this study's results using a variety of bioinformatics techniques, further in vitro assessment will be required to confirm the accuracy of the results.

## 5 Conclusion

In this study, 2369 phytoconstituents from Japanese medicinal plants were identified through extensive literature mining. These compounds underwent screening against the 3CL^pro^ of MERS-CoV to identify potential inhibitors of 3CL^pro^. Molecular docking analysis facilitated the selection of the three best compounds based on their binding energy, namely Kihadanin B, Robustaflavone, and 3-beta-O-(*trans*-p-Coumaroyl)maslinic acid. These three hit molecules predominantly interacted with the active site of 3CL^pro^, playing a crucial role in inhibiting the function of the 3CL^pro^ protein. The ADMET profiling results for these three lead molecules confirmed their lower toxicity and maximum drug-likeness properties. The stability and rigidity of these protein-ligand complexes throughout the simulation period were confirmed through a molecular dynamics simulation study. In comparison to the inhibitor Nafamostat, the top three compounds exhibited superior binding energy, stability, and firmness, as further validated by the calculation of binding free energy. This study relies entirely on computational screening and simulation programs; therefore, additional in vitro assessments will be necessary to validate the efficacy of these compounds against MERS-CoV.

## Funding

This research work receives no external funding.

## Data availability

All data generated or analyzed during this study are included in this published article.

## CRediT authorship contribution statement

**Suvro Biswas:** Writing – original draft, Investigation, Data curation, Conceptualization. **Mohasana Akter Mita:** Writing – original draft, Investigation, Formal analysis, Data curation, Conceptualization. **Shamima Afrose:** Writing – original draft, Methodology, Investigation, Formal analysis, Data curation. **Md Robiul Hasan:** Writing – original draft, Methodology, Investigation, Formal analysis, Data curation. **Mst Sharmin Sultana Shimu:** Formal analysis, Data curation. **Shahriar Zaman:** Writing – review & editing, Resources, Project administration. **Md Abu Saleh:** Writing – review & editing, Supervision, Resources, Project administration, Conceptualization.

## Declaration of competing interest

The authors declare that they have no known competing financial interests or personal relationships that could have appeared to influence the work reported in this paper.
