# Supplementary material for: An in silico approach to develop potential therapies against Middle East Respiratory Syndrome Coronavirus (MERS-CoV)
Source: Heliyon. 2024 Feb 9;10(4):e25837. doi: 10.1016/j.heliyon.2024.e25837 (PMC10877303; doi:10.1016/j.heliyon.2024.e25837)
Supplement: Multimedia component 1 [file mmc1.docx]

| **SL. No.** | **Plant Name** | **Compounds** | **CID** |
| --- | --- | --- | --- |
| 1. | *Asarum sieboldii*  (43) | Methylpluviatilol^1^  Pluviatilol^1^  Epipinoresinol^1^  Sesamin^1^  Piperitol^1^  Methyleugenol^1^  (±)-car-3-ene-2,5-dione^1^  -N-isobutyl-2,4,8,10-dodecatetraenamide^1^  Myristicin^2^  Safrole^2^  Asarinol A^3^  Asarinol B^3^  Asarinol C^3^  (+)-Asarinol D^3^  3-Caren-2-one^3^  m-Cymene^3^  p-Cymene^3^  p-Cymen-7-ol^3^  2,3-Dehydro-1,8-cineol^3^  Fenchone^3^  Myrcenol^3^  Phellandral^3^  Sabinyl acetate^3^  Terpinen-4-yl acetate^3^  Calarene^3^  Alpha-Guaiene^3^  Beta-Guaiene^3^  Isoledene^3^  Sativene^3^  3',4'-Dimethoxycinnamaldehyde^3^  3,5-Dimethoxytoluene^3^  2,3,5-Trimethoxytoluene^3^  Alpha-Asarone^3^  Beta-Asarone^3^  Croweacin^3^  Elemicin^3^  (E)-Isocroweacin^3^  Methyl kakuol^3^  3,4-Methylene-dioxypropiophenone^3^  Asarinin^3^  Asatone^3^  Tetradecane^3^  1-Tridecene^3^ | [5320622](https://pubchem.ncbi.nlm.nih.gov/compound/5320622)  [70695727](https://pubchem.ncbi.nlm.nih.gov/compound/70695727)  [637584](https://pubchem.ncbi.nlm.nih.gov/compound/637584)  [72307](https://pubchem.ncbi.nlm.nih.gov/compound/72307)  [10247670](https://pubchem.ncbi.nlm.nih.gov/compound/10247670)  [7127](https://pubchem.ncbi.nlm.nih.gov/compound/7127)  [181910](https://pubchem.ncbi.nlm.nih.gov/compound/181910)  [57404464](https://pubchem.ncbi.nlm.nih.gov/compound/57404464)  [4276](https://pubchem.ncbi.nlm.nih.gov/compound/4276)  [5144](https://pubchem.ncbi.nlm.nih.gov/compound/5144)  [46174001](https://pubchem.ncbi.nlm.nih.gov/compound/46174001)  [46174002](https://pubchem.ncbi.nlm.nih.gov/compound/46174002)  [21631051](https://pubchem.ncbi.nlm.nih.gov/compound/21631051)  [21631052](https://pubchem.ncbi.nlm.nih.gov/compound/21631052)  [13901618](https://pubchem.ncbi.nlm.nih.gov/compound/13901618)  [10812](https://pubchem.ncbi.nlm.nih.gov/compound/10812)  [7463](https://pubchem.ncbi.nlm.nih.gov/compound/7463)  [325](https://pubchem.ncbi.nlm.nih.gov/compound/325)  [523035](https://pubchem.ncbi.nlm.nih.gov/compound/523035)  [14525](https://pubchem.ncbi.nlm.nih.gov/compound/14525)  [10975](https://pubchem.ncbi.nlm.nih.gov/compound/10975)  [89488](https://pubchem.ncbi.nlm.nih.gov/compound/89488)  [94266](https://pubchem.ncbi.nlm.nih.gov/compound/94266)  [20960](https://pubchem.ncbi.nlm.nih.gov/compound/20960)  [28481](https://pubchem.ncbi.nlm.nih.gov/compound/28481)  [5317844](https://pubchem.ncbi.nlm.nih.gov/compound/5317844)  [6949](https://pubchem.ncbi.nlm.nih.gov/compound/6949)  [530426](https://pubchem.ncbi.nlm.nih.gov/compound/530426)  [11830550](https://pubchem.ncbi.nlm.nih.gov/compound/11830550)  [5375268](https://pubchem.ncbi.nlm.nih.gov/compound/5375268)  [77844](https://pubchem.ncbi.nlm.nih.gov/compound/77844)  [170114](https://pubchem.ncbi.nlm.nih.gov/compound/170114)  [636822](https://pubchem.ncbi.nlm.nih.gov/compound/636822)  [5281758](https://pubchem.ncbi.nlm.nih.gov/compound/5281758)  [5316141](https://pubchem.ncbi.nlm.nih.gov/compound/5316141)  [10248](https://pubchem.ncbi.nlm.nih.gov/compound/10248)  [14601153](https://pubchem.ncbi.nlm.nih.gov/compound/14601153)  [13672435](https://pubchem.ncbi.nlm.nih.gov/compound/13672435)  [95682](https://pubchem.ncbi.nlm.nih.gov/compound/95682)  [11869417](https://pubchem.ncbi.nlm.nih.gov/compound/11869417)  [431129](https://pubchem.ncbi.nlm.nih.gov/compound/431129)  [12389](https://pubchem.ncbi.nlm.nih.gov/compound/12389)  [17095](https://pubchem.ncbi.nlm.nih.gov/compound/17095) |
| 2. | *Scutellaria baicalensis*  (30) | Acetophenone^4^  l-phenyl-1^4^  (E)-4-phenyl-3-buten-2- one^4^  palmitic acid^4^  oleic acid^4^  b-carotene^4^  benzyl alcohol^4^  b-sitosterol^4^  Benzoic acid^4^  Lutein^4^  Baicalin^4^  Baicalein^4^  Wogonoside^4^  Wogonin^4^  Tenaxin‐I^4^  Viscidulin III^4^  Viscidulin I^4^  Viscidulin II^4^  Baicalein‐7‐O‐D‐glucoside^4^  Norwogonin^4^  Chrysin^4^  Chrysin‐8‐C‐β‐D‐glucopyranoside^4^  Skullcapflavone II^4^  Salvigenin^4^  Oroxylin A^4^  Oroxylin A‐7‐O‐glucuronide^4^  Eriodictyol^4^  5‐Hydroxy‐7,8‐dimethoxyflavone^4^  Dihydrobaicalin^4^  Dihydrooroxylin A^4^ | [7410](https://pubchem.ncbi.nlm.nih.gov/compound/7410)  [11062753](https://pubchem.ncbi.nlm.nih.gov/compound/11062753)  [129825247](https://pubchem.ncbi.nlm.nih.gov/compound/129825247)  [985](https://pubchem.ncbi.nlm.nih.gov/compound/985)  [445639](https://pubchem.ncbi.nlm.nih.gov/compound/445639)  [5280489](https://pubchem.ncbi.nlm.nih.gov/compound/5280489)  [244](https://pubchem.ncbi.nlm.nih.gov/compound/244)  [222284](https://pubchem.ncbi.nlm.nih.gov/compound/222284)  [243](https://pubchem.ncbi.nlm.nih.gov/compound/243)  [5281243](https://pubchem.ncbi.nlm.nih.gov/compound/5281243)  [64982](https://pubchem.ncbi.nlm.nih.gov/compound/64982)  [5281605](https://pubchem.ncbi.nlm.nih.gov/compound/5281605)  [3084961](https://pubchem.ncbi.nlm.nih.gov/compound/3084961)  [5281703](https://pubchem.ncbi.nlm.nih.gov/compound/5281703)  [159029](https://pubchem.ncbi.nlm.nih.gov/compound/159029)  [5271991](https://pubchem.ncbi.nlm.nih.gov/compound/5271991)  [5320471](https://pubchem.ncbi.nlm.nih.gov/compound/5320471)  [5322059](https://pubchem.ncbi.nlm.nih.gov/compound/5322059)  [5320313](https://pubchem.ncbi.nlm.nih.gov/compound/5320313)  [5281674](https://pubchem.ncbi.nlm.nih.gov/compound/5281674)  [5281607](https://pubchem.ncbi.nlm.nih.gov/compound/5281607)  [44257620](https://pubchem.ncbi.nlm.nih.gov/compound/44257620)  [124211](https://pubchem.ncbi.nlm.nih.gov/compound/124211)  [161271](https://pubchem.ncbi.nlm.nih.gov/compound/161271)  [5320315](https://pubchem.ncbi.nlm.nih.gov/compound/5320315)  [14655551](https://pubchem.ncbi.nlm.nih.gov/compound/14655551)  [440735](https://pubchem.ncbi.nlm.nih.gov/compound/440735)  [188316](https://pubchem.ncbi.nlm.nih.gov/compound/188316)  [14135325](https://pubchem.ncbi.nlm.nih.gov/compound/14135325)  [5316733](https://pubchem.ncbi.nlm.nih.gov/compound/5316733) |
| 3. | *Cinnamomum cassia*  (86) | endo-borneol^5^  (−)-α-terpineol^5^  β-bisabolene^5^  α-bisabolol^5^  curcumene^5^  δ-cadinene^5^  espatulenol^5^  caryophyllene oxide^5^  α-muurolene^5^  α-cadinol^5^  coniferaldehyde^5^  cinnamylalcohol^5^  2-methoxycinnamaldehyde^5^  benzyl benzoate^5^  2-hydroxybenzaldehyde^5^  3-phenylpropanol^5^  Benzaldehyde^5^  phenylethyl alcohol^5^  benzenepropanal^5^ | [1201518](https://pubchem.ncbi.nlm.nih.gov/compound/1201518)  [443162](https://pubchem.ncbi.nlm.nih.gov/compound/443162)  [10104370](https://pubchem.ncbi.nlm.nih.gov/compound/10104370)  [1616126](https://pubchem.ncbi.nlm.nih.gov/compound/1616126)  [92139](https://pubchem.ncbi.nlm.nih.gov/compound/92139)  [12306054](https://pubchem.ncbi.nlm.nih.gov/compound/12306054)  [522266](https://pubchem.ncbi.nlm.nih.gov/compound/522266)  [1742210](https://pubchem.ncbi.nlm.nih.gov/compound/1742210)  [12306049](https://pubchem.ncbi.nlm.nih.gov/compound/12306049)  [6431302](https://pubchem.ncbi.nlm.nih.gov/compound/6431302)  [5280536](https://pubchem.ncbi.nlm.nih.gov/compound/5280536)  [5315892](https://pubchem.ncbi.nlm.nih.gov/compound/5315892)  [641298](https://pubchem.ncbi.nlm.nih.gov/compound/641298)  [2345](https://pubchem.ncbi.nlm.nih.gov/compound/2345)  [6998](https://pubchem.ncbi.nlm.nih.gov/compound/6998)  [31234](https://pubchem.ncbi.nlm.nih.gov/compound/31234)  [240](https://pubchem.ncbi.nlm.nih.gov/compound/240)  [6054](https://pubchem.ncbi.nlm.nih.gov/compound/6054)  [7707](https://pubchem.ncbi.nlm.nih.gov/compound/7707) |
|  |  | 1-terpineol^6^  cis-β-terpineol^6^  caryophyllene^6^  eugenol^6^  cinnamyl acetate^6^  linalool^7^  camphene^7^  β-pinene^7^  camphor^7^  geranyl acetate^7^  copaene^7^  Cedrene^7^  α-calacorene^7^  isoledene^7^  cis-cinnamaldehyde^7^  trans-cinnamaldehyde^7^  ethyl cinnamate^7^  benzene,1,3-dimethyl^7^ | [11468](https://pubchem.ncbi.nlm.nih.gov/compound/11468)  [8748](https://pubchem.ncbi.nlm.nih.gov/compound/8748)  [5281515](https://pubchem.ncbi.nlm.nih.gov/compound/5281515)  [3314](https://pubchem.ncbi.nlm.nih.gov/compound/3314)  [5282110](https://pubchem.ncbi.nlm.nih.gov/compound/5282110)  [6549](https://pubchem.ncbi.nlm.nih.gov/compound/6549)  [6616](https://pubchem.ncbi.nlm.nih.gov/compound/6616)  [440967](https://pubchem.ncbi.nlm.nih.gov/compound/440967)  [2537](https://pubchem.ncbi.nlm.nih.gov/compound/2537)  [1549026](https://pubchem.ncbi.nlm.nih.gov/compound/1549026)  [19725](https://pubchem.ncbi.nlm.nih.gov/compound/19725)  [521207](https://pubchem.ncbi.nlm.nih.gov/compound/521207)  [528708](https://pubchem.ncbi.nlm.nih.gov/compound/528708)  [530426](https://pubchem.ncbi.nlm.nih.gov/compound/530426)  [6428995](https://pubchem.ncbi.nlm.nih.gov/compound/6428995)  [637511](https://pubchem.ncbi.nlm.nih.gov/compound/637511)  [637758](https://pubchem.ncbi.nlm.nih.gov/compound/637758)  [7929](https://pubchem.ncbi.nlm.nih.gov/compound/7929) |
|  |  | styrene^7^  1,3-pentanediol,2,2,4-trimethyl^7^  Decanal^7^  dodecane, 2,6,10-trimethyl^7^  coumarin^7^  cinnzeylanol^8^  anhydrocinnzeylanol^8^  cinnzeylanone^8^  Anhydrocinnzeylanine^8^  cinncassiol A^9^  cinncassiol B^9^  cinncassiol C^9^  cinncassiol E^9^  cinnacasol^9^  perseanol^10^  cinncassiol D1^10^  D1 glucoside^10^  D2 glucoside^10^  D4 glucoside^10^ | [7501](https://pubchem.ncbi.nlm.nih.gov/compound/7501)  [71330332](https://pubchem.ncbi.nlm.nih.gov/compound/71330332)  [8175](https://pubchem.ncbi.nlm.nih.gov/compound/8175)  [6430052](https://pubchem.ncbi.nlm.nih.gov/compound/6430052)  [323](https://pubchem.ncbi.nlm.nih.gov/compound/323)  [44559448](https://pubchem.ncbi.nlm.nih.gov/compound/44559448)  [73099741](https://pubchem.ncbi.nlm.nih.gov/compound/73099741)  [101693755](https://pubchem.ncbi.nlm.nih.gov/compound/101693755)  [131752069](https://pubchem.ncbi.nlm.nih.gov/compound/131752069)  [46173967](https://pubchem.ncbi.nlm.nih.gov/compound/46173967)  [71448932](https://pubchem.ncbi.nlm.nih.gov/compound/71448932)  [75144796](https://pubchem.ncbi.nlm.nih.gov/compound/75144796)  [139031772](https://pubchem.ncbi.nlm.nih.gov/compound/139031772)  [102421920](https://pubchem.ncbi.nlm.nih.gov/compound/102421920)  [21672106](https://pubchem.ncbi.nlm.nih.gov/compound/21672106)  [139031768](https://pubchem.ncbi.nlm.nih.gov/compound/139031768)  [131751606](https://pubchem.ncbi.nlm.nih.gov/compound/131751606)  [131751607](https://pubchem.ncbi.nlm.nih.gov/compound/131751607)  [131751659](https://pubchem.ncbi.nlm.nih.gov/compound/131751659) |
|  |  | germacrene D^11^  cis-2-methoxycinnamic acid^11^  2,2,4,6,6-pentamethylheptane^11^  2,5,9-trimethyldecane^11^  2-ethyl-5-propylphenol^11^  3,4-dimethoxyphenethyl alcohol^11^  2,5-dimethylundecane^11^  2-hydroxycinnamic acid^11^  2-hydroxycinnamaldehyde^12^  4-methoxycinnamaldehyde^12^  cinnamic acid^12^  cinnacasolide A^12^  cinnacasolide B^13^  cinnacasolide C^13^ | [5317570](https://pubchem.ncbi.nlm.nih.gov/compound/5317570)  [1622530](https://pubchem.ncbi.nlm.nih.gov/compound/1622530)  [26058](https://pubchem.ncbi.nlm.nih.gov/compound/26058)  [522020](https://pubchem.ncbi.nlm.nih.gov/compound/522020)  [522454](https://pubchem.ncbi.nlm.nih.gov/compound/522454)  [81911](https://pubchem.ncbi.nlm.nih.gov/compound/81911)  [28452](https://pubchem.ncbi.nlm.nih.gov/compound/28452)  [637540](https://pubchem.ncbi.nlm.nih.gov/compound/637540)  [5318169](https://pubchem.ncbi.nlm.nih.gov/compound/5318169)  [641294](https://pubchem.ncbi.nlm.nih.gov/compound/641294)  [444539](https://pubchem.ncbi.nlm.nih.gov/compound/444539)  [70692598](https://pubchem.ncbi.nlm.nih.gov/compound/70692598)  [70692599](https://pubchem.ncbi.nlm.nih.gov/compound/70692599)  [70694646](https://pubchem.ncbi.nlm.nih.gov/compound/70694646) |
|  |  | rosavin^13^  dihydromelilotoside^13^  methyl dihydromelilotoside^13^  cinnacasside A^14^  cinnacasside C^14^  cinnacasside B^15^  picrasmalignan A^16^  (−)-syringaresinol^16^  (+)-isolariciresinol^16^  (−)-secoisolariciresinol^16^  Lawsonicin^16^  5-methoxylariciresinol^16^  (+)-5,5 -dimethoxylariciresinol^16^  (1→6)-β-d-glucopyranoside^17^  (−)-lyoniresinol 3α-O-β-d-glucopyranoside^17^  5R-methyl-3-heptatriacontyl-2(5H)-furanone^18^ | [9823887](https://pubchem.ncbi.nlm.nih.gov/compound/9823887)  [5316728](https://pubchem.ncbi.nlm.nih.gov/compound/5316728)  [46881368](https://pubchem.ncbi.nlm.nih.gov/compound/46881368)  [25214691](https://pubchem.ncbi.nlm.nih.gov/compound/25214691)  [25214689](https://pubchem.ncbi.nlm.nih.gov/compound/25214689)  [102035893](https://pubchem.ncbi.nlm.nih.gov/compound/102035893)  [122395445](https://pubchem.ncbi.nlm.nih.gov/compound/122395445)  [11604108](https://pubchem.ncbi.nlm.nih.gov/compound/11604108)  [160521](https://pubchem.ncbi.nlm.nih.gov/compound/160521)  [65373](https://pubchem.ncbi.nlm.nih.gov/compound/65373)  [70698092](https://pubchem.ncbi.nlm.nih.gov/compound/70698092)  [44559567](https://pubchem.ncbi.nlm.nih.gov/compound/44559567)  [14157882](https://pubchem.ncbi.nlm.nih.gov/compound/14157882)  [476541](https://pubchem.ncbi.nlm.nih.gov/compound/476541)  [10483388](https://pubchem.ncbi.nlm.nih.gov/compound/10483388)  [129882098](https://pubchem.ncbi.nlm.nih.gov/compound/129882098) |
| 4. | *Glycyrrhiza uralensis*  (75) | Echinatin^19^  Glycyrin^19^  Glycyrol^19^  Isoangustone A^19^  Isoglycyrol^19^  7-O-methylluteone^19^  Glyasperin D^19^  Dehydroglyasperin D^19^  Gancaonin I^19^  Semilicoisoflavone B^19^  Kumatakenin^19^  Licoricone^19^  Angustone A^19^  Licoflavonol^19^  Topazolin^19^  Licoisoflavone A^19^  Wighteone^19^  Allolicoisoflavone B^19^  Lupiwighteone^19^  Licoarylcoumarin^19^  Isolicoflavonol^19^ | [6442675](https://pubchem.ncbi.nlm.nih.gov/compound/6442675)  [480787](https://pubchem.ncbi.nlm.nih.gov/compound/480787)  [5320083](https://pubchem.ncbi.nlm.nih.gov/compound/5320083)  [21591148](https://pubchem.ncbi.nlm.nih.gov/compound/21591148)  [124050](https://pubchem.ncbi.nlm.nih.gov/compound/124050)  [441251](https://pubchem.ncbi.nlm.nih.gov/compound/441251)  [480860](https://pubchem.ncbi.nlm.nih.gov/compound/480860)  [10109594](https://pubchem.ncbi.nlm.nih.gov/compound/10109594)  [480777](https://pubchem.ncbi.nlm.nih.gov/compound/480777)  [5481948](https://pubchem.ncbi.nlm.nih.gov/compound/5481948)  [5318869](https://pubchem.ncbi.nlm.nih.gov/compound/5318869)  [5319013](https://pubchem.ncbi.nlm.nih.gov/compound/5319013)  [15664151](https://pubchem.ncbi.nlm.nih.gov/compound/15664151)  [5481964](https://pubchem.ncbi.nlm.nih.gov/compound/5481964)  [5481965](https://pubchem.ncbi.nlm.nih.gov/compound/5481965)  [5281789](https://pubchem.ncbi.nlm.nih.gov/compound/5281789)  [5281814](https://pubchem.ncbi.nlm.nih.gov/compound/5281814)  [10383349](https://pubchem.ncbi.nlm.nih.gov/compound/10383349)  [5317480](https://pubchem.ncbi.nlm.nih.gov/compound/5317480)  [10090416](https://pubchem.ncbi.nlm.nih.gov/compound/10090416)  [5318585](https://pubchem.ncbi.nlm.nih.gov/compound/5318585) |
|  |  | Formononetin^19^  Genkwanin^19^  Glycycoumarin^19^  Isoglabrone^19^  Glyurallin A^19^  11b-hydroxy-11b,1-dihydromedicarpin^19^  Kaempferol 3-O-methyl ether^19^  Glycyrrhiza-isoflavone C^19^  Isoliquiritigenin^19^  Isoglycycoumarin^19^  Genistein^19^  Luteone^19^  Licocoumarone^19^  Uralenol^19^  Glicoricone^19^  Pratensein^19^  Dehydroglyasperin C^19^  Homobutein^19^  Kaempferol^19^  Gancaonin L^19^ | [5280378](https://pubchem.ncbi.nlm.nih.gov/compound/5280378)  [5281617](https://pubchem.ncbi.nlm.nih.gov/compound/5281617)  [5317756](https://pubchem.ncbi.nlm.nih.gov/compound/5317756)  [102597283](https://pubchem.ncbi.nlm.nih.gov/compound/102597283)  [15818598](https://pubchem.ncbi.nlm.nih.gov/compound/15818598)  [44437741](https://pubchem.ncbi.nlm.nih.gov/compound/44437741)  [5280862](https://pubchem.ncbi.nlm.nih.gov/compound/5280862)  [10546844](https://pubchem.ncbi.nlm.nih.gov/compound/10546844)  [638278](https://pubchem.ncbi.nlm.nih.gov/compound/638278)  [14187587](https://pubchem.ncbi.nlm.nih.gov/compound/14187587)  [5280961](https://pubchem.ncbi.nlm.nih.gov/compound/5280961)  [5281797](https://pubchem.ncbi.nlm.nih.gov/compound/5281797)  [503731](https://pubchem.ncbi.nlm.nih.gov/compound/503731)  [5315126](https://pubchem.ncbi.nlm.nih.gov/compound/5315126)  [10361658](https://pubchem.ncbi.nlm.nih.gov/compound/10361658)  [5281803](https://pubchem.ncbi.nlm.nih.gov/compound/5281803)  [480775](https://pubchem.ncbi.nlm.nih.gov/compound/480775)  [6438092](https://pubchem.ncbi.nlm.nih.gov/compound/6438092)  [5280863](https://pubchem.ncbi.nlm.nih.gov/compound/5280863)  [14604077](https://pubchem.ncbi.nlm.nih.gov/compound/14604077) |
|  |  | Licoisoflavanone^19^  biochanin A^19^  Liquiritigenin^19^  Licoflavone A^19^  Glyasperin C^19^  Daidzein^19^  1-methoxyphaseollin^19^  Isolupalbigenin^19^  6,8-diprenylgenistein^19^  2'-hydroxyisolupalbigenin^19^  Isoderrone^19^  Licoricidin^19^  Glicophenone^19^  Licoisoflavone B^19^  Vicenin-2^19^  Isoviolanthin^19^  Liquiritin apioside^19^  Liquiritin^19^  Ononin^19^  Isoliquiritin^19^  Glycyroside^19^  Sophoraflavone B^19^  Daidzin^19^  Glycyrrhizic acid^20^  lignoceric acid^20^  methoxyficifolinol^20^  docosanol^20^  betulinic acid^20^  docosyl caffeate^20^  gancaonin H^20^  3R-vestitol^20^  7,2',4'-trihydroxy-5-methoxy-3-arylcoumarin^20^  isoliquiritin apioside^20^  hedysarimcoumestan B^20^ | [392443](https://pubchem.ncbi.nlm.nih.gov/compound/392443)  [5280373](https://pubchem.ncbi.nlm.nih.gov/compound/5280373)  [114829](https://pubchem.ncbi.nlm.nih.gov/compound/114829)  [5319000](https://pubchem.ncbi.nlm.nih.gov/compound/5319000)  [480859](https://pubchem.ncbi.nlm.nih.gov/compound/480859)  [5281708](https://pubchem.ncbi.nlm.nih.gov/compound/5281708)  131753054  [26238934](https://pubchem.ncbi.nlm.nih.gov/compound/26238934)  [480783](https://pubchem.ncbi.nlm.nih.gov/compound/480783)  [14237659](https://pubchem.ncbi.nlm.nih.gov/compound/14237659)  [14237660](https://pubchem.ncbi.nlm.nih.gov/compound/14237660)  [480865](https://pubchem.ncbi.nlm.nih.gov/compound/480865)  [10021298](https://pubchem.ncbi.nlm.nih.gov/compound/10021298)  [5481234](https://pubchem.ncbi.nlm.nih.gov/compound/5481234)  [442664](https://pubchem.ncbi.nlm.nih.gov/compound/442664)  [101422758](https://pubchem.ncbi.nlm.nih.gov/compound/101422758)  [10076238](https://pubchem.ncbi.nlm.nih.gov/compound/10076238)  [503737](https://pubchem.ncbi.nlm.nih.gov/compound/503737)  [442813](https://pubchem.ncbi.nlm.nih.gov/compound/442813)  [5318591](https://pubchem.ncbi.nlm.nih.gov/compound/5318591)  [101939210](https://pubchem.ncbi.nlm.nih.gov/compound/101939210)  [5491513](https://pubchem.ncbi.nlm.nih.gov/compound/5491513)  [107971](https://pubchem.ncbi.nlm.nih.gov/compound/107971)  [14982](https://pubchem.ncbi.nlm.nih.gov/compound/14982)  [11197](https://pubchem.ncbi.nlm.nih.gov/compound/11197)  [480872](https://pubchem.ncbi.nlm.nih.gov/compound/480872)  [12620](https://pubchem.ncbi.nlm.nih.gov/compound/12620)  [64971](https://pubchem.ncbi.nlm.nih.gov/compound/64971)  [5316952](https://pubchem.ncbi.nlm.nih.gov/compound/5316952)  [5481949](https://pubchem.ncbi.nlm.nih.gov/compound/5481949)  [439310](https://pubchem.ncbi.nlm.nih.gov/compound/439310)  [25015742](https://pubchem.ncbi.nlm.nih.gov/compound/25015742)  [6442433](https://pubchem.ncbi.nlm.nih.gov/compound/6442433)  [11558452](https://pubchem.ncbi.nlm.nih.gov/compound/11558452) |
| 5. | *Astragalus membranaceus*  (29) | AH-1^21^  astragaloside I^21^  [Astragaloside-II](https://pubchem.ncbi.nlm.nih.gov/compound/71306915)^21^  Astragaloside III^21^  Astragaloside IV^21^  Astragaloside V^21^  Astragaloside VI^21^  Astragaloside VII^21^  Isoastragaloside I^21^  Isoastragaloside II^21^  Azukisaponin V methyl ester^21^  Malonylastragaloside^21^  astramembrannin II^21^  brachyoside B^21^  cyclocanthoside E^21^  astramembranoside A^21^  astramembranoside B^21^  Astragalan^21^  Kaempferol^21^  Isorhamnetin^21^  Rhamnocitrin^21^  kumatakenin^21^  rhamnocitrin-3-glucoside^21^  quercetin-3-glucoside^21^  Formononetin^21^  Calycosin^21^  2′-hydroxy-3′,4′,7-trimethoxyisoflavone^21^  3,9,10-trimethoxypterocarpan^21^  Astragalus polysaccharides (APS)^21^ | [132525571](https://pubchem.ncbi.nlm.nih.gov/compound/132525571)  [13996685](https://pubchem.ncbi.nlm.nih.gov/compound/13996685)  [71306915](https://pubchem.ncbi.nlm.nih.gov/compound/71306915)  [441905](https://pubchem.ncbi.nlm.nih.gov/compound/441905)  [13943297](https://pubchem.ncbi.nlm.nih.gov/compound/13943297)  [71448939](https://pubchem.ncbi.nlm.nih.gov/compound/71448939)  [71448940](https://pubchem.ncbi.nlm.nih.gov/compound/71448940)  [14241100](https://pubchem.ncbi.nlm.nih.gov/compound/14241100)  [60148697](https://pubchem.ncbi.nlm.nih.gov/compound/60148697)  [60148655](https://pubchem.ncbi.nlm.nih.gov/compound/60148655)  [101683319](https://pubchem.ncbi.nlm.nih.gov/compound/101683319)  [50917280](https://pubchem.ncbi.nlm.nih.gov/compound/50917280)  [132492418](https://pubchem.ncbi.nlm.nih.gov/compound/132492418)  [100927164](https://pubchem.ncbi.nlm.nih.gov/compound/100927164)  [21633193](https://pubchem.ncbi.nlm.nih.gov/compound/21633193)  [24795974](https://pubchem.ncbi.nlm.nih.gov/compound/24795974)  [24796110](https://pubchem.ncbi.nlm.nih.gov/compound/24796110)  [103026223](https://pubchem.ncbi.nlm.nih.gov/substance/103026223)  [5280863](https://pubchem.ncbi.nlm.nih.gov/compound/5280863)  [5281654](https://pubchem.ncbi.nlm.nih.gov/compound/5281654)  [5320946](https://pubchem.ncbi.nlm.nih.gov/compound/5320946)  [5318869](https://pubchem.ncbi.nlm.nih.gov/compound/5318869)  [44259544](https://pubchem.ncbi.nlm.nih.gov/compound/44259544)  [5280804](https://pubchem.ncbi.nlm.nih.gov/compound/5280804)  [5280378](https://pubchem.ncbi.nlm.nih.gov/compound/5280378)  [5280448](https://pubchem.ncbi.nlm.nih.gov/compound/5280448)  [44257240](https://pubchem.ncbi.nlm.nih.gov/compound/44257240)  [15689655](https://pubchem.ncbi.nlm.nih.gov/compound/15689655)  [2782115](https://pubchem.ncbi.nlm.nih.gov/compound/2782115) |
| 6. | *Phellodendron amurense*  (66) | Berberine^22^  Berberastine^22^  Bis-[4-(dimethylamino)phenyl]methanone^22^  Evodiamine^22^  Palmatine^22^  Tetrahydropalmatine^22^  Tetrahydroberberine^22^  Phellodendrine^22^  Magnocurarine^22^  Magnoflorine^22^  *Ƴ*-Fagarine^22^  Canthin-6-one^22^  4-methoxy-N-methyl-2-quinolone^22^  Oxypalmatine^22^  Candicine^22^  Lotusine^22^ | [2353](https://pubchem.ncbi.nlm.nih.gov/compound/2353)  [442180](https://pubchem.ncbi.nlm.nih.gov/compound/442180)  [7031](https://pubchem.ncbi.nlm.nih.gov/compound/282874)  [442088](https://pubchem.ncbi.nlm.nih.gov/compound/442088)  [19009](https://pubchem.ncbi.nlm.nih.gov/compound/19009)  [5417](https://pubchem.ncbi.nlm.nih.gov/compound/5417)  [34458](https://pubchem.ncbi.nlm.nih.gov/compound/34458)  [3081405](https://pubchem.ncbi.nlm.nih.gov/compound/3081405)  [53266](https://pubchem.ncbi.nlm.nih.gov/compound/53266)  [73337](https://pubchem.ncbi.nlm.nih.gov/compound/73337)  [107936](https://pubchem.ncbi.nlm.nih.gov/compound/107936)  [97176](https://pubchem.ncbi.nlm.nih.gov/compound/97176)  [182073](https://pubchem.ncbi.nlm.nih.gov/compound/182073)  [10926678](https://pubchem.ncbi.nlm.nih.gov/compound/10926678)  [23135](https://pubchem.ncbi.nlm.nih.gov/compound/23135)  [5274587](https://pubchem.ncbi.nlm.nih.gov/compound/5274587) |
|  |  | Tetrahydrojatrorrhizine^22^  Menisperine^22^  (+) N-methylcorydine^22^  N-methylflindersine^22^  Litcubine^22^  Xanthoplanine^22^  N-methylphoebine^22^  Columbamine^22^  Epiberberine^22^ | [185605](https://pubchem.ncbi.nlm.nih.gov/compound/185605)  [161487](https://pubchem.ncbi.nlm.nih.gov/compound/161487)  [52949574](https://pubchem.ncbi.nlm.nih.gov/compound/52949574)  [72819](https://pubchem.ncbi.nlm.nih.gov/compound/72819)  [85243417](https://pubchem.ncbi.nlm.nih.gov/compound/85243417)  [14262868](https://pubchem.ncbi.nlm.nih.gov/compound/14262868)  [101731569](https://pubchem.ncbi.nlm.nih.gov/compound/101731569)  [72310](https://pubchem.ncbi.nlm.nih.gov/compound/72310)  [160876](https://pubchem.ncbi.nlm.nih.gov/compound/160876) |
|  |  | Pteleine^22^  Noroxyhydrastinine^22^  Chilenine^22^  Rutecarpine^22^  Skimmianine^22^  Tembetarine^22^  Tetramethyl-O-scutellarin^22^  *Ƴ*-hydroxybutenolide^22^  Armepavine^22^  Demethyleneberberine^22^  8-oxoberberine^22^  8-oxoepiberberine^22^  Oxyberberine^22^  Kihadanin B^22^  Niloticin^22^  Obaculactone or limonin^22^  Obacunone or Obacunoic acid^22^ | [159650](https://pubchem.ncbi.nlm.nih.gov/compound/159650)  [89047](https://pubchem.ncbi.nlm.nih.gov/compound/89047)  [11025386](https://pubchem.ncbi.nlm.nih.gov/compound/11025386)  [65752](https://pubchem.ncbi.nlm.nih.gov/compound/65752)  [6760](https://pubchem.ncbi.nlm.nih.gov/compound/6760)  [167718](https://pubchem.ncbi.nlm.nih.gov/compound/167718)  [96118](https://pubchem.ncbi.nlm.nih.gov/compound/96118)  [16221764](https://pubchem.ncbi.nlm.nih.gov/compound/16221764)  [442169](https://pubchem.ncbi.nlm.nih.gov/compound/442169)  [363209](https://pubchem.ncbi.nlm.nih.gov/compound/363209)  [12039004](https://pubchem.ncbi.nlm.nih.gov/compound/12039004)  [12799036](https://pubchem.ncbi.nlm.nih.gov/compound/12799036)  [11066](https://pubchem.ncbi.nlm.nih.gov/compound/11066)  [156766](https://pubchem.ncbi.nlm.nih.gov/compound/156766)  [14021529](https://pubchem.ncbi.nlm.nih.gov/compound/14021529)  [179651](https://pubchem.ncbi.nlm.nih.gov/compound/179651)  [119041](https://pubchem.ncbi.nlm.nih.gov/compound/119041) |
|  |  | Rutaevin^22^  Coniferin^22^  Vanilloloside^22^  Herculin^22^  Ferulic acid^22^  Quinic acid^22^  Neochlorogenic acid^22^  3-O-feruloylquinic acid^22^  Chlorogenic acid^22^  Methyl 3-*O*-feruloylquinate^22^  Methyl 5-O-feruloylquinate^22^  (+/-)-lyoniresinol^22^  Amurensin^22^  Quercetin^22^  Phellamurin^22^  Phellatin^22^  Phellavin^22^  Phellodendroside^22^  Icariside-1^22^  Phellamuretin^22^  Isovaleric acid^22^  7-Dehydrostigmasterol^22^  Syringin^22^  Daucosterol^22^ | [441805](https://pubchem.ncbi.nlm.nih.gov/compound/441805)  [5280372](https://pubchem.ncbi.nlm.nih.gov/compound/5280372)  [44577222](https://pubchem.ncbi.nlm.nih.gov/compound/44577222)  [5318023](https://pubchem.ncbi.nlm.nih.gov/compound/5318023)  [445858](https://pubchem.ncbi.nlm.nih.gov/compound/445858)  [6508](https://pubchem.ncbi.nlm.nih.gov/compound/6508)  [5280633](https://pubchem.ncbi.nlm.nih.gov/compound/5280633)  [9799386](https://pubchem.ncbi.nlm.nih.gov/compound/9799386)  [1794427](https://pubchem.ncbi.nlm.nih.gov/compound/1794427)  [24813764](https://pubchem.ncbi.nlm.nih.gov/compound/24813764)  [102004731](https://pubchem.ncbi.nlm.nih.gov/compound/102004731)  [10483388](https://pubchem.ncbi.nlm.nih.gov/compound/10483388)  [5318156](https://pubchem.ncbi.nlm.nih.gov/compound/5318156)  [5280343](https://pubchem.ncbi.nlm.nih.gov/compound/5280343)  [193876](https://pubchem.ncbi.nlm.nih.gov/compound/193876)  [44258781](https://pubchem.ncbi.nlm.nih.gov/compound/44258781)  [5320517](https://pubchem.ncbi.nlm.nih.gov/compound/5320517)  [101316827](https://pubchem.ncbi.nlm.nih.gov/compound/101316827)  [5745470](https://pubchem.ncbi.nlm.nih.gov/compound/5745470)  [7092536](https://pubchem.ncbi.nlm.nih.gov/compound/7092536)  [10430](https://pubchem.ncbi.nlm.nih.gov/compound/10430)  [12303924](https://pubchem.ncbi.nlm.nih.gov/compound/12303924)  [5316860](https://pubchem.ncbi.nlm.nih.gov/compound/5316860)  [5742590](https://pubchem.ncbi.nlm.nih.gov/compound/5742590) |
| 7. | *Mentha arvensis*  (15) | α-Pinene^23^  Sabinene^23^  β-Myrcene^23^  d-Limonene^23^  (Z)-β-Ocimene^23^  (E)-β-Ocimene^23^  3-Octanol^23^  l-Menthone^23^  Isomenthone^23^  Menthyl acetate^23^  Neomenthol^23^  Caryophyllene^23^  l-Menthol^23^  α-Terpineol^23^  Piperitone^23^ | [6654](https://pubchem.ncbi.nlm.nih.gov/compound/6654)  [18818](https://pubchem.ncbi.nlm.nih.gov/compound/18818)  [31253](https://pubchem.ncbi.nlm.nih.gov/compound/31253)  [440917](https://pubchem.ncbi.nlm.nih.gov/compound/440917)  [5320250](https://pubchem.ncbi.nlm.nih.gov/compound/5320250)  [5281553](https://pubchem.ncbi.nlm.nih.gov/compound/5281553)  [11527](https://pubchem.ncbi.nlm.nih.gov/compound/11527)  [26447](https://pubchem.ncbi.nlm.nih.gov/compound/26447)  [6986](https://pubchem.ncbi.nlm.nih.gov/compound/6986)  [27867](https://pubchem.ncbi.nlm.nih.gov/compound/27867)  [19243](https://pubchem.ncbi.nlm.nih.gov/compound/19243)  [5281515](https://pubchem.ncbi.nlm.nih.gov/compound/5281515)  [16666](https://pubchem.ncbi.nlm.nih.gov/compound/16666)  [17100](https://pubchem.ncbi.nlm.nih.gov/compound/17100)  [6987](https://pubchem.ncbi.nlm.nih.gov/compound/6987) |
| 8. | *Bupleurum falcatum*  (17) | 5-O-caffeoyl quinic acid^24^  chlorogenic acid methyl ester^24^  quercetin^24^  quercitrin^24^  isoquercitrin^24^  rutin^24^  saikosaponin D^24^  saikosaponin A^24^  saikosaponin C^24^  2,2,4-trimethyl-3-penten-1-ol^25^  2,3-dimethyl-3-buten-2-ol^25^  2,3-dimethyl-pentane^25^  9-octadecenoic acid (z)^25^  octadecanoic acid^25^  nonanedioic acid^25^  octanedioic acid^25^  6-O-acetyl-saikosaponin d^25^ | [5280633](https://pubchem.ncbi.nlm.nih.gov/compound/5280633)  [6476139](https://pubchem.ncbi.nlm.nih.gov/compound/6476139)  [5280343](https://pubchem.ncbi.nlm.nih.gov/compound/5280343)  [5280459](https://pubchem.ncbi.nlm.nih.gov/compound/5280459)  [5280804](https://pubchem.ncbi.nlm.nih.gov/compound/5280804)  [5280805](https://pubchem.ncbi.nlm.nih.gov/compound/5280805)  [107793](https://pubchem.ncbi.nlm.nih.gov/compound/107793)  [167928](https://pubchem.ncbi.nlm.nih.gov/compound/167928)  [131801344](https://pubchem.ncbi.nlm.nih.gov/compound/131801344)  [79926](https://pubchem.ncbi.nlm.nih.gov/compound/79926)  [82652](https://pubchem.ncbi.nlm.nih.gov/compound/82652)  [11260](https://pubchem.ncbi.nlm.nih.gov/compound/11260)  [6439696](https://pubchem.ncbi.nlm.nih.gov/compound/6439696)  [5281](https://pubchem.ncbi.nlm.nih.gov/compound/5281)  [2266](https://pubchem.ncbi.nlm.nih.gov/compound/2266)  [10457](https://pubchem.ncbi.nlm.nih.gov/compound/10457)  [21637630](https://pubchem.ncbi.nlm.nih.gov/compound/21637630) |
| 9. | *Paeonia lactiflora*  (43) | Palbinone^26^  13-Methyl tetradecanoic acid^26^  Peonin (peonidin-3,5-diglucoside)^26^  Albiflorin^26^  Benzoylpaeoniflorin^26^  8-Debenzoylpaeoniflorin^26^  Paeonidanin B^26^  Paeonidanin A^26^  Paeonidanin C^26^  Paeoniflorigenone^26^  Paeoniflorin^26^  Paeonilactone A^26^  Paeonilactone B^26^  Paeonilactone C^26^  Pyrethrin I^26^  Pyrethrin II^26^  β-Sitosterol^26^  Casuariin^26^  Casuarictin^26^  5-Desgalloylstachyurin^26^  1-O-galloyl-β-D-glucose^26^  3-O-Galloylquinic acid^26^  4-O-Galloylquinic acid^26^  Pedunculagin^26^  1,2,3,4,6-Pentagalloylglucose^26^  Strictinin^26^  Tellimagrandin I^26^  1,3,6-Trigalloyl-β-D-glucose^26^  1,2,3-Tri-O-galloyl-β-D-glucose^26^  Benzoic acid^26^  Phenol (Carbolic acid)^26^ | [9841735](https://pubchem.ncbi.nlm.nih.gov/compound/9841735)  [151014](https://pubchem.ncbi.nlm.nih.gov/compound/151014)  [44256843](https://pubchem.ncbi.nlm.nih.gov/compound/44256843)  [24868421](https://pubchem.ncbi.nlm.nih.gov/compound/24868421)  [21631106](https://pubchem.ncbi.nlm.nih.gov/compound/21631106)  [71452333](https://pubchem.ncbi.nlm.nih.gov/compound/71452333)  [102417825](https://pubchem.ncbi.nlm.nih.gov/compound/102417825)  [44253993](https://pubchem.ncbi.nlm.nih.gov/compound/44253993)  [46883513](https://pubchem.ncbi.nlm.nih.gov/compound/46883513)  [70698143](https://pubchem.ncbi.nlm.nih.gov/compound/70698143)  [442534](https://pubchem.ncbi.nlm.nih.gov/compound/442534)  [10081437](https://pubchem.ncbi.nlm.nih.gov/compound/10081437)  [10375422](https://pubchem.ncbi.nlm.nih.gov/compound/10375422)  [10471123](https://pubchem.ncbi.nlm.nih.gov/compound/10471123)  [5281045](https://pubchem.ncbi.nlm.nih.gov/compound/5281045)  [5281555](https://pubchem.ncbi.nlm.nih.gov/compound/5281555)  [521199](https://pubchem.ncbi.nlm.nih.gov/compound/521199)  [14035442](https://pubchem.ncbi.nlm.nih.gov/compound/14035442)  [73644](https://pubchem.ncbi.nlm.nih.gov/compound/73644)  [10417809](https://pubchem.ncbi.nlm.nih.gov/compound/10417809)  [124021](https://pubchem.ncbi.nlm.nih.gov/compound/124021)  [442988](https://pubchem.ncbi.nlm.nih.gov/compound/442988)  [475263](https://pubchem.ncbi.nlm.nih.gov/compound/475263)  [442688](https://pubchem.ncbi.nlm.nih.gov/compound/442688)  [65238](https://pubchem.ncbi.nlm.nih.gov/compound/65238)  [73330](https://pubchem.ncbi.nlm.nih.gov/compound/73330)  [442690](https://pubchem.ncbi.nlm.nih.gov/compound/442690)  [452707](https://pubchem.ncbi.nlm.nih.gov/compound/452707)  [13270010](https://pubchem.ncbi.nlm.nih.gov/compound/13270010)  [243](https://pubchem.ncbi.nlm.nih.gov/compound/243)  [996](https://pubchem.ncbi.nlm.nih.gov/compound/996) |
|  |  | p-hydroxybenzoic acid^27^  gallic acid^27^  methylgallate^27^  paeonol^27^  paeonoside^27^  (+)-catechin^27^  Oxypaeoniflorin^27^  Benzoyloxypaeoniflorin^27^  Galloylpaeoniflorin^27^  betulinic acid^27^  hederagenin^27^  oleanolic acid^27^ | [135](https://pubchem.ncbi.nlm.nih.gov/compound/135)  [370](https://pubchem.ncbi.nlm.nih.gov/compound/370)  [7428](https://pubchem.ncbi.nlm.nih.gov/compound/7428)  [11092](https://pubchem.ncbi.nlm.nih.gov/compound/11092)  [442924](https://pubchem.ncbi.nlm.nih.gov/compound/442924)  [9064](https://pubchem.ncbi.nlm.nih.gov/compound/9064)  [21631105](https://pubchem.ncbi.nlm.nih.gov/compound/21631105)  [21631107](https://pubchem.ncbi.nlm.nih.gov/compound/21631107)  [46882879](https://pubchem.ncbi.nlm.nih.gov/compound/46882879)  [64971](https://pubchem.ncbi.nlm.nih.gov/compound/64971)  [73299](https://pubchem.ncbi.nlm.nih.gov/compound/73299)  [10494](https://pubchem.ncbi.nlm.nih.gov/compound/10494) |
| 10. | *Atractylodes lancea*  (28) | Atractylenolide I^28^  Atractylenolide II^28^  Atractylenolide III^28^  Atractylone^28^  Hinesol^28^  β-Eudesmol^28^  Atractylodin^28^  Stigmasterol^28^  coumarin osthol^29^  atractylochromene^29^  Kudtdiol^29^  atractyloside A^29^  atractyloside B^29^  pterocarpol^29^  eudesma-4(14),7(11)-dien-8-one^29^  atractyloside C^29^  atractyloside D^29^  atractyloside E^29^  atractyloside G^29^  atractyloside I^29^  atractyloside F^29^  heterolupeol^29^  Atractylodinol^29^  Acetylatractylodinol^29^  1,3-di-O-caffeoylquinic acid^29^  5-hydroxymethyl furaldehyde^29^  L-phenylalanine^29^  diethyl phthalate^29^ | [5321018](https://pubchem.ncbi.nlm.nih.gov/compound/5321018)  [14448070](https://pubchem.ncbi.nlm.nih.gov/compound/14448070)  [155948](https://pubchem.ncbi.nlm.nih.gov/compound/155948)  [3080635](https://pubchem.ncbi.nlm.nih.gov/compound/3080635)  [10878761](https://pubchem.ncbi.nlm.nih.gov/compound/10878761)  [91714393](https://pubchem.ncbi.nlm.nih.gov/compound/91714393)  [5321047](https://pubchem.ncbi.nlm.nih.gov/compound/5321047)  [5280794](https://pubchem.ncbi.nlm.nih.gov/compound/5280794)  [10228](https://pubchem.ncbi.nlm.nih.gov/compound/10228)  [10244247](https://pubchem.ncbi.nlm.nih.gov/compound/10244247)  [26471785](https://pubchem.ncbi.nlm.nih.gov/compound/26471785)  [71307451](https://pubchem.ncbi.nlm.nih.gov/compound/71307451)  [71448952](https://pubchem.ncbi.nlm.nih.gov/compound/71448952)  [12314741](https://pubchem.ncbi.nlm.nih.gov/compound/12314741)  [13986100](https://pubchem.ncbi.nlm.nih.gov/compound/13986100)  [71448953](https://pubchem.ncbi.nlm.nih.gov/compound/71448953)  [71448954](https://pubchem.ncbi.nlm.nih.gov/compound/71448954)  [71448955](https://pubchem.ncbi.nlm.nih.gov/compound/71448955)  [71448957](https://pubchem.ncbi.nlm.nih.gov/compound/71448957)  [10929902](https://pubchem.ncbi.nlm.nih.gov/compound/10929902)  [71448956](https://pubchem.ncbi.nlm.nih.gov/compound/71448956)  [604983](https://pubchem.ncbi.nlm.nih.gov/compound/604983)  [10012964](https://pubchem.ncbi.nlm.nih.gov/compound/10012964)  [5315531](https://pubchem.ncbi.nlm.nih.gov/compound/5315531)  [6474640](https://pubchem.ncbi.nlm.nih.gov/compound/6474640)  [237332](https://pubchem.ncbi.nlm.nih.gov/compound/237332)  [6140](https://pubchem.ncbi.nlm.nih.gov/compound/6140)  6781 |

**References:**

1. Quang, T. H. *et al.* Anti-inflammatory and PPAR transactivational effects of secondary metabolites from the roots of Asarum sieboldii. *Bioorganic Med. Chem. Lett.* **22**, 2527–2533 (2012).

2. Yi, J. H., Perumalsamy, H., Sankarapandian, K., Choi, B. R. & Ahn, Y. J. Fumigant Toxicity of Phenylpropanoids Identified in Asarum sieboldii Aerial Parts to Lycoriella ingenua (Diptera: Sciaridae) and Coboldia fuscipes (Diptera: Scatopsidae). *J. Econ. Entomol.* **108**, 1208–1214 (2015).

3. Antsyshkina, A. M. *et al.* The genus asarum l.: A phytochemical and ethnopharmacological review. *Syst. Rev. Pharm.* **11**, 472–502 (2020).

4. Zhao, T. *et al.* Scutellaria baicalensis Georgi. (Lamiaceae): a review of its traditional uses, botany, phytochemistry, pharmacology and toxicology. *J. Pharm. Pharmacol.* **71**, 1353–1369 (2019).

5. Zhang, C. *et al.* Cinnamomum cassia Presl: A review of its traditional uses, phytochemistry, pharmacology and toxicology. *Molecules* **24**, (2019).

6. Pannee, C., Chandhanee, I. & Wacharee, L. Antiinflammatory effects of essential oil from the leaves of Cinnamomum cassia and cinnamaldehyde on lipopolysaccharide-stimulated J774A.1 cells. *J. Adv. Pharm. Technol. Res.* **5**, 164–170 (2014).

7. Li, Y. Q., Kong, D. X. & Wu, H. Analysis and evaluation of essential oil components of cinnamon barks using GC-MS and FTIR spectroscopy. *Ind. Crops Prod.* **41**, 269–278 (2013).

8. Yan, Y. M. *et al.* Anti-diabetic nephropathy compounds from Cinnamomum cassia. *J. Ethnopharmacol.* **165**, 141–147 (2015).

9. Pham, V. C. *et al.* Five new diterpenoids from the barks of Cinnamomum cassia (L.) J. Presl. *Phytochem. Lett.* **32**, 23–28 (2019).

10. Zeng, J. *et al.* Diterpenoids with immunosuppressive activities from cinnamomum cassia. *J. Nat. Prod.* **77**, 1948–1954 (2014).

11. Chang, C.-T., Chang, W.-L., Hsu, J.-C., Shih, Y. & Chou, S.-T. Chemical composition and tyrosinase inhibitory activity of Cinnamomum cassia essential oil. *Bot. Stud.* **54**, 10 (2013).

12. Chen, P. Y., Yu, J. W., Lu, F. L., Lin, M. C. & Cheng, H. F. Differentiating parts of Cinnamomum cassia using LC-qTOF-MS in conjunction with principal component analysis. *Biomed. Chromatogr.* **30**, 1449–1457 (2016).

13. Ngoc, T. M. *et al.* Xanthine oxidase inhibitory activity of constituents of Cinnamomum cassia twigs. *Bioorganic Med. Chem. Lett.* **22**, 4625–4628 (2012).

14. Zhou, W., Liang, Z., Li, P., Zhao, Z. & Chen, J. Tissue-specific chemical profiling and quantitative analysis of bioactive components of Cinnamomum cassia by combining laser-microdissection with UPLC-Q/TOF–MS. *Chem. Cent. J.* **12**, 1–9 (2018).

15. Zeng, J. F. *et al.* Two new geranylphenylacetate glycosides from the barks of Cinnamomum cassia. *Nat. Prod. Res.* **31**, 1812–1818 (2017).

16. He, S., Zeng, K. W., Jiang, Y. & Tu, P. F. Nitric oxide inhibitory constituents from the barks of Cinnamomum cassia. *Fitoterapia* **112**, 153–160 (2016).

17. Luo, Q., Wang, S. M., Lu, Q., Luo, J. & Cheng, Y. X. Identification of compounds from the water soluble extract of cinnamomum cassia barks and their inhibitory effects against high-glucose-induced mesangial cells. *Molecules* **18**, 10930–10943 (2013).

18. Li, Z., Cai, Z., Qian, S. & Chen, M. A New Lactone from the Twigs of Cinnamomum cassia. *Chem. Nat. Compd.* **53**, 234–236 (2017).

19. Ji, S. *et al.* Bioactive Constituents of Glycyrrhiza uralensis (Licorice): Discovery of the Effective Components of a Traditional Herbal Medicine. *J. Nat. Prod.* **79**, 281–292 (2016).

20. LIU, Y. *et al.* Studies on chemical constituents on roots of Glycyrrhiza uralensis. *Chinese J. Pharm. Anal.* **31**, 1251–1255 (2011).

21. Agyemang, K. *et al.* Recent advances in astragalus membranaceus anti-diabetic research: Pharmacological effects of its phytochemical constituents. *Evidence-based Complement. Altern. Med.* **2013**, (2013).

22. Sun, Y., Lenon, G. B. & Yang, A. W. H. Phellodendri Cortex: A Phytochemical, Pharmacological, and Pharmacokinetic Review. *Evidence-Based Complement. Altern. Med.* **2019**, 7621929 (2019).

23. Shimotori, Y. *et al.* Enzyme-assisted extraction of bioactive phytochemicals from Japanese peppermint (Mentha arvensis l. cv. ‘hokuto’). *J. Oleo Sci.* **69**, 635–642 (2020).

24. Tung, N. H., Uto, T., Morinaga, O. & Shoyama, Y. Chemical constituents from the aerial parts of Bupleurum falcatum L. and biological evidences. *Nat. Prod. Sci.* **21**, 71–75 (2015).

25. Liu, X.-H., Li, M.-J., Wang, H. & Yang, C. R. Chemical constituents analysis on the seeds of Bupleurum Falcatum L. *Fenxi Huaxue* **28**, 1083–1084 (2000).

26. Parker, S. *et al.* A Pharmacological Review of Bioactive Constituents of Paeonia lactiflora Pallas and Paeonia veitchii Lynch. *Phyther. Res.* 1445–1473 (2016) doi:10.1002/ptr.5653.

27. Koo, Y. K. *et al.* Platelet anti-aggregatory and blood anti-coagulant effects of compounds isolated from Paeonia lactiflora and Paeonia suffruticosa. *Pharmazie* **65**, 624–628 (2010).

28. Jun, X., Fu, P., Lei, Y. & Cheng, P. Pharmacological effects of medicinal components of Atractylodes lancea (Thunb.) DC. *Chinese Med. (United Kingdom)* **13**, 1–10 (2018).

29. Zhang, W. jin *et al.* Atractylodis Rhizoma: A review of its traditional uses, phytochemistry, pharmacology, toxicology and quality control. *J. Ethnopharmacol.* **266**, 113415 (2021).
