# Supplementary material for: An in silico approach to develop potential therapies against Middle East Respiratory Syndrome Coronavirus (MERS-CoV)
Source: Heliyon. 2024 Feb 9;10(4):e25837. doi: 10.1016/j.heliyon.2024.e25837 (PMC10877303; doi:10.1016/j.heliyon.2024.e25837)
Supplement: Multimedia component 2 [file mmc2.docx]

| **SL.**  **No.** | **Plant Name** | **Compounds** | **CID** |
| --- | --- | --- | --- |
| 11. | *Cimicifuga dahurica*  (27) | Geniposide^1^  24-O-acetylhydroshengmanol^1^   prim-O-glucosylcimifugin^1^  acerinol^1^  cimigenol^1^  Cimiracemoside C^2^  Cimigenoside^2^  25-O-Acetylcimigenol^2^  25-Anhydrocimigenol^2^  23-epi-26-Deoxyactein^2^  Actein^2^  Cimicifugic acid A^2^  Cimicifugic acid B^2^  Cimicifugic acid D^2^  Cimicifugamide^2^  Visnagin^2^  2**-**Isoferuloyl piscidic acid^3^  Ferulic acid 4-O-β-d-glucopyranoside^3^  Caffeic acid 3-O-β-d-glucopyranoside^3^  Shomaside A^3^  Caffeic ester glucoside^3^  Caffeic methl ester^3^  Carboxymethyl isoferulate^3^  3, 4-Dimethoxycinnamic acid^3^  2**-**Feruloyl piscidic acid^3^  (E)-3-(3-Methyl-2-butenylidene)-2-indolinone^3^  (Z)-3-(3-Methyl-2-butenylidene)-2-indolinone^3^ | [107848](https://pubchem.ncbi.nlm.nih.gov/compound/107848)  [157168](https://pubchem.ncbi.nlm.nih.gov/compound/157168)  [14034912](https://pubchem.ncbi.nlm.nih.gov/compound/14034912)  [73347277](https://pubchem.ncbi.nlm.nih.gov/compound/73347277)  [16020000](https://pubchem.ncbi.nlm.nih.gov/compound/16020000)  [15541911](https://pubchem.ncbi.nlm.nih.gov/compound/15541911)  [16088242](https://pubchem.ncbi.nlm.nih.gov/compound/16088242)  [46881255](https://pubchem.ncbi.nlm.nih.gov/compound/46881255)  [15967434](https://pubchem.ncbi.nlm.nih.gov/compound/15967434)  [21668683](https://pubchem.ncbi.nlm.nih.gov/compound/21668683)  [10032468](https://pubchem.ncbi.nlm.nih.gov/compound/10032468)  [6449879](https://pubchem.ncbi.nlm.nih.gov/compound/6449879)  [6449880](https://pubchem.ncbi.nlm.nih.gov/compound/6449880)  [11742743](https://pubchem.ncbi.nlm.nih.gov/compound/11742743)  [5318530](https://pubchem.ncbi.nlm.nih.gov/compound/5318530)  [6716](https://pubchem.ncbi.nlm.nih.gov/compound/6716)  6450179  13916049  5281759  46209783  5281761  689075  16729361  717531  10002902  5319526  5249879 |
| 12. | *Cnidium officinale*  (08) | 6-(1-oxopentyl)-salicylic acid methyl ester^4^  Falcarindiol^4^  conifenyl ferulate^4^  ferulic aldehyde^4^  senkyunolide C^4^  (E)-3-methoxy-1-(3-methoxyphenyl)propane^4^  Coniferol^4^  p-hydroxyphenol^4^ | [134612870](https://pubchem.ncbi.nlm.nih.gov/compound/134612870)  [5281148](https://pubchem.ncbi.nlm.nih.gov/compound/5281148)  [6441913](https://pubchem.ncbi.nlm.nih.gov/compound/6441913)  [5280536](https://pubchem.ncbi.nlm.nih.gov/compound/5280536)  [642374](https://pubchem.ncbi.nlm.nih.gov/compound/642374)  [15418925](https://pubchem.ncbi.nlm.nih.gov/compound/15418925)  [1549095](https://pubchem.ncbi.nlm.nih.gov/compound/1549095)  [785](https://pubchem.ncbi.nlm.nih.gov/compound/785) |
| 13. | *Angelica dahurica*  (28) | Isoimperatorin^5^  imperatorin^5^  bergapten^5^  osthenol^5^  xanthotoxin^5^  dehydrogeijerin^5^  phellopterin^5^  7-demethylsuberosin^5^  Alloimperatorin^5^  Xanthotoxol^5^   Isooxypeucedanin^5^  Alloisoimperatorin^5^  5-hydroxy-8-methoxypsoralen^5^  oxypeucedanin methanolate^5^  pabulenol^5^  byakangelicin^5^  marmesin^5^  (+) -decursinol^5^  Heraclenol^5^  oxypeucedanin hydrate^5^  marmesinin^5^  ulopterol^5^  uracil^5^  oxypeucedanin^6^  byakangelicol^6^  isopimpinellin^6^  pimpinellin^6^  scopoletin^6^ | [68081](https://pubchem.ncbi.nlm.nih.gov/compound/68081)  [10212](https://pubchem.ncbi.nlm.nih.gov/compound/10212)  [2355](https://pubchem.ncbi.nlm.nih.gov/compound/2355)  [5320318](https://pubchem.ncbi.nlm.nih.gov/compound/5320318)  [4114](https://pubchem.ncbi.nlm.nih.gov/compound/4114)  [620900](https://pubchem.ncbi.nlm.nih.gov/compound/620900)  [98608](https://pubchem.ncbi.nlm.nih.gov/compound/98608)  [5316525](https://pubchem.ncbi.nlm.nih.gov/compound/5316525)  [69502](https://pubchem.ncbi.nlm.nih.gov/compound/69502)  [65090](https://pubchem.ncbi.nlm.nih.gov/compound/65090)  [625383](https://pubchem.ncbi.nlm.nih.gov/compound/625383)  [5317436](https://pubchem.ncbi.nlm.nih.gov/compound/5317436)  [5385192](https://pubchem.ncbi.nlm.nih.gov/compound/5385192)  [483514](https://pubchem.ncbi.nlm.nih.gov/compound/483514)  [3009225](https://pubchem.ncbi.nlm.nih.gov/compound/3009225)  [10211](https://pubchem.ncbi.nlm.nih.gov/compound/10211)  [334704](https://pubchem.ncbi.nlm.nih.gov/compound/334704)  [442127](https://pubchem.ncbi.nlm.nih.gov/compound/442127)  [73253](https://pubchem.ncbi.nlm.nih.gov/compound/73253)  [17536](https://pubchem.ncbi.nlm.nih.gov/compound/17536)  [216283](https://pubchem.ncbi.nlm.nih.gov/compound/216283)  [176475](https://pubchem.ncbi.nlm.nih.gov/compound/176475)  [1174](https://pubchem.ncbi.nlm.nih.gov/compound/1174)  [160544](https://pubchem.ncbi.nlm.nih.gov/compound/160544)  [3055167](https://pubchem.ncbi.nlm.nih.gov/compound/3055167)  [68079](https://pubchem.ncbi.nlm.nih.gov/compound/68079)  [4825](https://pubchem.ncbi.nlm.nih.gov/compound/4825)  [5280460](https://pubchem.ncbi.nlm.nih.gov/compound/5280460) |
| 14. | *Ephedra sinica*  (54) | 1-Phenylpropane-1,2-dione^7^  (S)-cathinone^7^  (1R,2S)-norephedrine^7^  (2S,4S,5R)-2,4-dimethyl-5-phenyl oxazolidine^7^  (1R,2S)-ephedrine^7^  (1R,2S)-N-methylephedrine^7^  (1S,2S)-norpseudoephedrine^7^  (1S,2S)-pseudoephedrine^7^  (1S,2S)-N-methylpseudoephedrine^7^  proanthocyanidin A4^7^  catechin^8^  epicatechin^8^  gallocatechin^8^  epigallocatechin^8^  Ephedradine A^8^  Ephedradine B^9^  Ephedradine C^9^  Ephedradine D^9^  Feruloylhistamine^9^  D(–)Methylephedrine^9^  Ephedroxane^9^  2, 3, 4-Trimethyl-5-phenyloxazolidine^9^  Hordenine^9^  Maokonine^9^  Tetramethylpyrazine^9^  N-methylbenzylamine^9^  Herbacetin^9^  Herbacetin 7-methyl ether^9^  Pollenitin^9^ | [11363](https://pubchem.ncbi.nlm.nih.gov/compound/11363)  [25203656](https://pubchem.ncbi.nlm.nih.gov/compound/25203656)  [10297](https://pubchem.ncbi.nlm.nih.gov/compound/10297)  [130754154](https://pubchem.ncbi.nlm.nih.gov/compound/130754154)  [6922965](https://pubchem.ncbi.nlm.nih.gov/compound/6922965)  [6918907](https://pubchem.ncbi.nlm.nih.gov/compound/6918907)  [6918945](https://pubchem.ncbi.nlm.nih.gov/compound/6918945)  [7028](https://pubchem.ncbi.nlm.nih.gov/compound/7028)  [7059595](https://pubchem.ncbi.nlm.nih.gov/compound/7059595)  [13556387](https://pubchem.ncbi.nlm.nih.gov/compound/13556387)  [9064](https://pubchem.ncbi.nlm.nih.gov/compound/9064)  [72276](https://pubchem.ncbi.nlm.nih.gov/compound/72276)  [65084](https://pubchem.ncbi.nlm.nih.gov/compound/65084)  [72277](https://pubchem.ncbi.nlm.nih.gov/compound/72277)  [126326](https://pubchem.ncbi.nlm.nih.gov/compound/126326)  [156055](https://pubchem.ncbi.nlm.nih.gov/compound/156055)  [558490](https://pubchem.ncbi.nlm.nih.gov/compound/558490)  [100996000](https://pubchem.ncbi.nlm.nih.gov/compound/100996000)  [10401784](https://pubchem.ncbi.nlm.nih.gov/compound/10401784)  [3041454](https://pubchem.ncbi.nlm.nih.gov/compound/3041454)  [161171](https://pubchem.ncbi.nlm.nih.gov/compound/161171)  [5322112](https://pubchem.ncbi.nlm.nih.gov/compound/5322112)  [68313](https://pubchem.ncbi.nlm.nih.gov/compound/68313)  [54704413](https://pubchem.ncbi.nlm.nih.gov/compound/54704413)  [14296](https://pubchem.ncbi.nlm.nih.gov/compound/14296)  [7669](https://pubchem.ncbi.nlm.nih.gov/compound/7669)  [5280544](https://pubchem.ncbi.nlm.nih.gov/compound/5280544)  [44259958](https://pubchem.ncbi.nlm.nih.gov/compound/44259958)  [44259965](https://pubchem.ncbi.nlm.nih.gov/compound/44259965) |
|  |  | Herbacetin 7-O-glucoside^9^  Kaempferol 3-O-rhamnoside 7-O-glucoside^9^  Herbacetin 7-O-neohesperidoside^9^  Kaempferol-3-O-glucoside-7-O-rhamnoside^9^  Dihydroquercetin^9^  Naringenin^9^  Hesperidin^9^  (–)-epiafzelechin^9^  Afzelechin^9^  Leucocyanidin^9^  Symplocoside^9^  Tricin^9^  Apigenin^9^  3-Methoxyherbacetin^9^  Swertisin^9^  Isovitexin-2″-O-rhamnoside^9^  Leucodelphinidin^9^  Ephedrannin A^9^  Ephedrannin B^9^  Sesquipinsapol B^9^  Ethyl caprylate^9^  Sitosterol^9^  Trans-cinnamic acid^9^  Physcion^9^  Rhein^9^ | [5318021](https://pubchem.ncbi.nlm.nih.gov/compound/5318021)  [554033](https://pubchem.ncbi.nlm.nih.gov/compound/554033)  [102444811](https://pubchem.ncbi.nlm.nih.gov/compound/102444811)  [14035324](https://pubchem.ncbi.nlm.nih.gov/compound/14035324)  [439533](https://pubchem.ncbi.nlm.nih.gov/compound/439533)  [932](https://pubchem.ncbi.nlm.nih.gov/compound/932)  [10621](https://pubchem.ncbi.nlm.nih.gov/compound/10621)  [443639](https://pubchem.ncbi.nlm.nih.gov/compound/443639)  [442154](https://pubchem.ncbi.nlm.nih.gov/compound/442154)  [71629](https://pubchem.ncbi.nlm.nih.gov/compound/71629)  [44257110](https://pubchem.ncbi.nlm.nih.gov/compound/44257110)  [5281702](https://pubchem.ncbi.nlm.nih.gov/compound/5281702)  [5280443](https://pubchem.ncbi.nlm.nih.gov/compound/5280443)  [5319442](https://pubchem.ncbi.nlm.nih.gov/compound/5319442)  [124034](https://pubchem.ncbi.nlm.nih.gov/compound/124034)  [23844078](https://pubchem.ncbi.nlm.nih.gov/compound/23844078)  [3081374](https://pubchem.ncbi.nlm.nih.gov/compound/3081374)  [21676348](https://pubchem.ncbi.nlm.nih.gov/compound/21676348)  [25051177](https://pubchem.ncbi.nlm.nih.gov/compound/25051177)  [101767126](https://pubchem.ncbi.nlm.nih.gov/compound/101767126)  [7799](https://pubchem.ncbi.nlm.nih.gov/compound/7799)  [222284](https://pubchem.ncbi.nlm.nih.gov/compound/222284)  [444539](https://pubchem.ncbi.nlm.nih.gov/compound/444539)  [10639](https://pubchem.ncbi.nlm.nih.gov/compound/10639)  [10168](https://pubchem.ncbi.nlm.nih.gov/compound/10168) |
| 15. | *Panax ginseng*  (26) | Panaxadione^10^  20(S)-protopanaxatriol^10^  3-keto-20(S)-protopanaxatriol^10^  ginsenoside Rg2^10^  ginsenoside Rd^10^  phenethyl alcohol- xylopyranosyl(1→6)glucopyranoside^10^  panaxoside A^11^  [Panaxoside Re](https://pubchem.ncbi.nlm.nih.gov/compound/58774156)^11^  protopanaxadiol^11^  panacon^11^  panaxadiol^11^  panaxol^11^  ginsenoside R0^11^  ginsenoside Rb-1^11^  ginsenoside Rb-2^11^  ginsenoside Rc^11^  ginsenoside Rf^11^  ginsenoside Rg-3^11^  ginsenoside Rh-1^11^  ginsenoside Rh-2^11^  panacene^11^  β-elemene^11^  citric acid^11^  fumaric acid^11^  ketoglutaric acid^11^  choline^11^ | [25233029](https://pubchem.ncbi.nlm.nih.gov/compound/25233029)  [11468733](https://pubchem.ncbi.nlm.nih.gov/compound/11468733)  [44198762](https://pubchem.ncbi.nlm.nih.gov/compound/44198762)  [21599924](https://pubchem.ncbi.nlm.nih.gov/compound/21599924)  [24721561](https://pubchem.ncbi.nlm.nih.gov/compound/24721561)    [131129](https://pubchem.ncbi.nlm.nih.gov/compound/131129)  [441923](https://pubchem.ncbi.nlm.nih.gov/compound/441923)  [58774156](https://pubchem.ncbi.nlm.nih.gov/compound/58774156)  [9920281](https://pubchem.ncbi.nlm.nih.gov/compound/9920281)  [6325774](https://pubchem.ncbi.nlm.nih.gov/compound/6325774)  [73498](https://pubchem.ncbi.nlm.nih.gov/compound/73498)  [328778](https://pubchem.ncbi.nlm.nih.gov/compound/328778)  [11815492](https://pubchem.ncbi.nlm.nih.gov/compound/11815492)  [9898279](https://pubchem.ncbi.nlm.nih.gov/compound/9898279)  [6917976](https://pubchem.ncbi.nlm.nih.gov/compound/6917976)  [12855889](https://pubchem.ncbi.nlm.nih.gov/compound/12855889)  [441922](https://pubchem.ncbi.nlm.nih.gov/compound/441922)  [9918693](https://pubchem.ncbi.nlm.nih.gov/compound/9918693)  [12855920](https://pubchem.ncbi.nlm.nih.gov/compound/12855920)  [119307](https://pubchem.ncbi.nlm.nih.gov/compound/119307)  [181799](https://pubchem.ncbi.nlm.nih.gov/compound/181799)  [6918391](https://pubchem.ncbi.nlm.nih.gov/compound/6918391)  [311](https://pubchem.ncbi.nlm.nih.gov/compound/311)  [444972](https://pubchem.ncbi.nlm.nih.gov/compound/444972)  [51](https://pubchem.ncbi.nlm.nih.gov/compound/51)  [305](https://pubchem.ncbi.nlm.nih.gov/compound/305) |
| 16. | *Zingiber officinale*  (45) | [6]-gingerol^12^  [6]-shogaol^12^  [4]-gingerol^12^  [7]-gingerol^12^  [8]-gingerol^12^  [10]-gingerol^12^  [6]-Paradol^12^  [7]- paradol^12^  [8]- paradol^12^  [10]- paradol^12^  methyl [6]-paradol^12^  [4]- shogaol^12^  [8]- shogaol^12^  [10]- shogaol^12^  [12]-shogaol^12^  [6]-isoshogaol^12^  [6]- gingerdione^12^  [8]- gingerdione^12^  [10]- gingerdione^12^  [12]- gingerdione^12^ | [442793](https://pubchem.ncbi.nlm.nih.gov/compound/442793)  [5281794](https://pubchem.ncbi.nlm.nih.gov/compound/5281794)  [5317596](https://pubchem.ncbi.nlm.nih.gov/compound/5317596)  [11472344](https://pubchem.ncbi.nlm.nih.gov/compound/11472344)  [168114](https://pubchem.ncbi.nlm.nih.gov/compound/168114)  [168115](https://pubchem.ncbi.nlm.nih.gov/compound/168115)  [94378](https://pubchem.ncbi.nlm.nih.gov/compound/94378)  [13733135](https://pubchem.ncbi.nlm.nih.gov/compound/13733135)  [213821](https://pubchem.ncbi.nlm.nih.gov/compound/213821)  [51352076](https://pubchem.ncbi.nlm.nih.gov/compound/51352076)  [85807832](https://pubchem.ncbi.nlm.nih.gov/compound/85807832)  [9794897](https://pubchem.ncbi.nlm.nih.gov/compound/9794897)  [6442560](https://pubchem.ncbi.nlm.nih.gov/compound/6442560)  [6442612](https://pubchem.ncbi.nlm.nih.gov/compound/6442612)  [9975813](https://pubchem.ncbi.nlm.nih.gov/compound/9975813)  [11694761](https://pubchem.ncbi.nlm.nih.gov/compound/11694761)  [162952](https://pubchem.ncbi.nlm.nih.gov/compound/162952)  [14440537](https://pubchem.ncbi.nlm.nih.gov/compound/14440537)  [14440539](https://pubchem.ncbi.nlm.nih.gov/compound/14440539)  [86251913](https://pubchem.ncbi.nlm.nih.gov/compound/86251913) |
|  |  | citral/ geranial^13^  borneol^13^  bisabolene^13^  Gingerdiol^13^  1-dehydrogingerdione^13^  5-acetoxy [6] gingerdiol^13^  Ar-curcumene^13^  β-bisabolene^13^  (-) β-sesquiphellandrene^13^  6-methyl-5-hepten-2-one^13^  α-phellandrene^13^  β-phellandrene^13^  limonene^13^  citronellol^13^  neral^13^  geraniol^13^  bornyl acetate^13^  2 undecanone^13^  citronellyl acetate^13^  α-copaene^13^  geranyl acetate^13^  eucalyptol/ 1,8-cineole^13^  isobornyl formate^14^  α-zingiberene^14^  α farnesene^14^ | [638011](https://pubchem.ncbi.nlm.nih.gov/compound/638011)  [64685](https://pubchem.ncbi.nlm.nih.gov/compound/64685)  [3033866](https://pubchem.ncbi.nlm.nih.gov/compound/3033866)  [11369949](https://pubchem.ncbi.nlm.nih.gov/compound/11369949)  [9796015](https://pubchem.ncbi.nlm.nih.gov/compound/9796015)  [101419545](https://pubchem.ncbi.nlm.nih.gov/compound/101419545)  [92139](https://pubchem.ncbi.nlm.nih.gov/compound/92139)  [10104370](https://pubchem.ncbi.nlm.nih.gov/compound/10104370)  [519764](https://pubchem.ncbi.nlm.nih.gov/compound/519764)  [9862](https://pubchem.ncbi.nlm.nih.gov/compound/9862)  [7460](https://pubchem.ncbi.nlm.nih.gov/compound/7460)  [11142](https://pubchem.ncbi.nlm.nih.gov/compound/11142)  [22311](https://pubchem.ncbi.nlm.nih.gov/compound/22311)  [8842](https://pubchem.ncbi.nlm.nih.gov/compound/8842)  [643779](https://pubchem.ncbi.nlm.nih.gov/compound/643779)  [637566](https://pubchem.ncbi.nlm.nih.gov/compound/637566)  [6448](https://pubchem.ncbi.nlm.nih.gov/compound/6448)  [8163](https://pubchem.ncbi.nlm.nih.gov/compound/8163)  [9017](https://pubchem.ncbi.nlm.nih.gov/compound/9017)  [70678558](https://pubchem.ncbi.nlm.nih.gov/compound/70678558)  [1549026](https://pubchem.ncbi.nlm.nih.gov/compound/1549026)  [2758](https://pubchem.ncbi.nlm.nih.gov/compound/2758)  [23623868](https://pubchem.ncbi.nlm.nih.gov/compound/23623868)  [521253](https://pubchem.ncbi.nlm.nih.gov/compound/521253)  [5281516](https://pubchem.ncbi.nlm.nih.gov/compound/5281516) |
| 17. | *Poria cocos*  (41) | dehydroeburicoic acid^15^  3-epi-dehydrotumulosic acid^15^  pachymic acid^15^  eburicoic acid^15^  trametenolic acid^15^  α-amyrin acetate^15^  adenosine^15^  16α-Hydroxytrametenolic acid^15^  3-O-Acetyl-16α-hydroxytrametenolic acid^16^  3-O-Acetyl-16α-hydroxydehydrotrametenolic acid^16^  Dehydrotrametenonic acid^16^  Dehydroeburiconic acid^16^  Dehydropachymic acid^16^  3-epi-Dehydropachymic acid^16^  Tumulosic acid^16^  Dehydrotumulosic acid^16^  15α-Hydroxydehydrotumulosic acid^16^  Polyporenic acid C^16^ | [15250826](https://pubchem.ncbi.nlm.nih.gov/compound/15250826)  [10005581](https://pubchem.ncbi.nlm.nih.gov/compound/10005581)  [5484385](https://pubchem.ncbi.nlm.nih.gov/compound/5484385)  [73402](https://pubchem.ncbi.nlm.nih.gov/compound/73402)  [12309443](https://pubchem.ncbi.nlm.nih.gov/compound/12309443)  [71597151](https://pubchem.ncbi.nlm.nih.gov/compound/71597151)  [60961](https://pubchem.ncbi.nlm.nih.gov/compound/60961)  [132285301](https://pubchem.ncbi.nlm.nih.gov/compound/132285301)  [15226712](https://pubchem.ncbi.nlm.nih.gov/compound/15226712)  [15226714](https://pubchem.ncbi.nlm.nih.gov/compound/15226714)  [44424826](https://pubchem.ncbi.nlm.nih.gov/compound/44424826)  [11431307](https://pubchem.ncbi.nlm.nih.gov/compound/11431307)  [15226717](https://pubchem.ncbi.nlm.nih.gov/compound/15226717)  [15226716](https://pubchem.ncbi.nlm.nih.gov/compound/15226716)  [12314446](https://pubchem.ncbi.nlm.nih.gov/compound/12314446)  [15225964](https://pubchem.ncbi.nlm.nih.gov/compound/15225964)  [16736459](https://pubchem.ncbi.nlm.nih.gov/compound/16736459)  9805290s |
|  |  | 6α-Hydroxypolyporenic acid C^16^  29-Hydroxypolyporenic acid C^16^  25-Hydroxy-3-epi-tumulosic acid^16^  5α-8α-Peroxydehydrotumulosic acid^16^  Poricoic acid B^16^  16-Deoxyporicoic acid B^16^  Poricoic acid E^16^  Poricoic acid BM^16^  Poricoic acid G^16^  Poricoic acid GM^16^  Poricoic acid A^16^  Poricoic acid C^16^  Poricoic acid D^16^  Poricoic acid F^16^  Poricoic acid H^16^  Poricoic acid CM^16^  Poricoic acid DM^16^  Poricoic acid HM^16^  6,7-Dehydroporicoic acid H^16^  25-Hydroxyporicoic acid C^16^  25-Hydroxyporicoic acid H^16^  26-Hydroxyporicoic acid DM^16^  25-Methoxyporicoic acid A^16^ | [101280198](https://pubchem.ncbi.nlm.nih.gov/compound/101280198)  [139585448](https://pubchem.ncbi.nlm.nih.gov/compound/139585448)  [46882675](https://pubchem.ncbi.nlm.nih.gov/compound/46882675)  [16736654](https://pubchem.ncbi.nlm.nih.gov/compound/16736654)  [5471852](https://pubchem.ncbi.nlm.nih.gov/compound/5471852)  [16736458](https://pubchem.ncbi.nlm.nih.gov/compound/16736458)  [15225966](https://pubchem.ncbi.nlm.nih.gov/compound/15225966)  [15225967](https://pubchem.ncbi.nlm.nih.gov/compound/15225967)  [5471966](https://pubchem.ncbi.nlm.nih.gov/compound/5471966)  [44556812](https://pubchem.ncbi.nlm.nih.gov/compound/44556812)  [5471851](https://pubchem.ncbi.nlm.nih.gov/compound/5471851)  [56668247](https://pubchem.ncbi.nlm.nih.gov/compound/56668247)  [44424827](https://pubchem.ncbi.nlm.nih.gov/compound/44424827)  [101928114](https://pubchem.ncbi.nlm.nih.gov/compound/101928114)  [10918099](https://pubchem.ncbi.nlm.nih.gov/compound/10918099)  [16736060](https://pubchem.ncbi.nlm.nih.gov/compound/16736060)  [44424830](https://pubchem.ncbi.nlm.nih.gov/compound/44424830)  [44556877](https://pubchem.ncbi.nlm.nih.gov/compound/44556877)  [44556878](https://pubchem.ncbi.nlm.nih.gov/compound/44556878)  [44556811](https://pubchem.ncbi.nlm.nih.gov/compound/44556811)  [16736865](https://pubchem.ncbi.nlm.nih.gov/compound/16736865)  [44556810](https://pubchem.ncbi.nlm.nih.gov/compound/44556810)  [46882628](https://pubchem.ncbi.nlm.nih.gov/compound/46882628) |
| 18. | *Paeonia suffruticosa*  (52) | Mudanpioside F^17^  Procyanidin B6^17^  Oxypaeoniflorin^17^  Mudanpioside E^17^  (þ)-Catechin^17^  Apiopaeonoside^17^  Methyl gallate^17^  Paeonolide^17^  1,3,6-Tri-O-galloyl-b-D-glucose^17^  Suffruticoside A^17^  Suffruticoside B^17^  Suffruticoside C^17^  Suffruticoside D^17^  Tetragalloyl glucose^17^  Quercetin 7-O-glucoside^17^  Galloylpaeoniflorin^17^  Albiflorin^17^  1,2,3,4,6-Penta-O-galloyol-beta-D glucose^17^  Mudanpioside H^17^  Hexagalloyl glucose^17^  Galloyloxypaeoniflorin^17^  Benzoyloxypaeoniflorin^17^  Quercetin 3-O-rhamnosylglucoside^17^  Mudanpioside C^17^  Benzoylpaeoniflorin^17^  Mudanpioside B^17^ | [21631108](https://pubchem.ncbi.nlm.nih.gov/compound/21631108)  [474540](https://pubchem.ncbi.nlm.nih.gov/compound/474540)  [21631105](https://pubchem.ncbi.nlm.nih.gov/compound/21631105)  [86278277](https://pubchem.ncbi.nlm.nih.gov/compound/86278277)  [107957](https://pubchem.ncbi.nlm.nih.gov/compound/107957)  [127509](https://pubchem.ncbi.nlm.nih.gov/compound/127509)  [7428](https://pubchem.ncbi.nlm.nih.gov/compound/7428)  [442923](https://pubchem.ncbi.nlm.nih.gov/compound/442923)  [452707](https://pubchem.ncbi.nlm.nih.gov/compound/452707)  [9986231](https://pubchem.ncbi.nlm.nih.gov/compound/9986231)  [10258205](https://pubchem.ncbi.nlm.nih.gov/compound/10258205)  [10258206](https://pubchem.ncbi.nlm.nih.gov/compound/10258206)  [5321547](https://pubchem.ncbi.nlm.nih.gov/compound/5321547)  [102012877](https://pubchem.ncbi.nlm.nih.gov/compound/102012877)  [5381351](https://pubchem.ncbi.nlm.nih.gov/compound/5381351)  [46882879](https://pubchem.ncbi.nlm.nih.gov/compound/46882879)  [24868421](https://pubchem.ncbi.nlm.nih.gov/compound/24868421)  [65238](https://pubchem.ncbi.nlm.nih.gov/compound/65238)  [71457654](https://pubchem.ncbi.nlm.nih.gov/compound/71457654)  [54036807](https://pubchem.ncbi.nlm.nih.gov/compound/54036807)  [3036133](https://pubchem.ncbi.nlm.nih.gov/compound/3036133)  [21631107](https://pubchem.ncbi.nlm.nih.gov/compound/21631107)  [5491657](https://pubchem.ncbi.nlm.nih.gov/compound/5491657)  [21631098](https://pubchem.ncbi.nlm.nih.gov/compound/21631098)  [21631106](https://pubchem.ncbi.nlm.nih.gov/compound/21631106)  [21631102](https://pubchem.ncbi.nlm.nih.gov/compound/21631102) |
|  |  | hederagenin^18^  chrysoeriol^18^  trans-ε-viniferin^18^  trans-resveratrol^18^  stigmasterol^18^  β-daucosterol^18^  quinic acid^19^  galloylquinic acid^19^  kaempferol 3,7-di-O-glucoside^19^  isorhamnetin 3,7-di-O-glucoside^19^  galloyl glucose^19^  hexagalloylglucose^19^  heptagalloylglucose^19^  Paeonisuffrone^19^  Paeonisuffral^20^  Deoxypaeonisuffrone^20^  mudanpioside A^20^  mudanpioside D^20^  α-benzoyloxypaeoniflorin^20^  oxypaeonidanin^20^  9-epi-oxypaeonidanin^20^  trigalloyl-glucose^20^  (−)-Epigallocatechin gallate^20^  uridine^20^   thymidine^20^  paeoniflorigenone^20^ | [73299](https://pubchem.ncbi.nlm.nih.gov/compound/73299)  [5280666](https://pubchem.ncbi.nlm.nih.gov/compound/5280666)  [5281728](https://pubchem.ncbi.nlm.nih.gov/compound/5281728)  [445154](https://pubchem.ncbi.nlm.nih.gov/compound/445154)  [5280794](https://pubchem.ncbi.nlm.nih.gov/compound/5280794)  [5742590](https://pubchem.ncbi.nlm.nih.gov/compound/5742590)  [6508](https://pubchem.ncbi.nlm.nih.gov/compound/6508)  [129650210](https://pubchem.ncbi.nlm.nih.gov/compound/129650210)  [6325460](https://pubchem.ncbi.nlm.nih.gov/compound/6325460)  [5323537](https://pubchem.ncbi.nlm.nih.gov/compound/5323537)  [124021](https://pubchem.ncbi.nlm.nih.gov/compound/124021)  [129630523](https://pubchem.ncbi.nlm.nih.gov/compound/129630523)  [25202629](https://pubchem.ncbi.nlm.nih.gov/compound/25202629)  [10104180](https://pubchem.ncbi.nlm.nih.gov/compound/10104180)  [101050848](https://pubchem.ncbi.nlm.nih.gov/compound/101050848)  [10035257](https://pubchem.ncbi.nlm.nih.gov/compound/10035257)  [21631101](https://pubchem.ncbi.nlm.nih.gov/compound/21631101)  [21631103](https://pubchem.ncbi.nlm.nih.gov/compound/21631103)  [102597515](https://pubchem.ncbi.nlm.nih.gov/compound/102597515)  [71457653](https://pubchem.ncbi.nlm.nih.gov/compound/71457653)  [71450490](https://pubchem.ncbi.nlm.nih.gov/compound/71450490)  [440308](https://pubchem.ncbi.nlm.nih.gov/compound/440308)  [65064](https://pubchem.ncbi.nlm.nih.gov/compound/65064)  [6029](https://pubchem.ncbi.nlm.nih.gov/compound/6029)  [5789](https://pubchem.ncbi.nlm.nih.gov/compound/5789)  [70698143](https://pubchem.ncbi.nlm.nih.gov/compound/70698143) |
| 19. | *Aconitum carmichaelii Debeaux*  (63) | Honokiol^21^  pinoresinol^21^  salicylic acid^21^  p-hydroxy-cinnamic acid^21^  songorine^21^  karakoline^22^  Aconitine^22^  Mesaconitine^22^  Hypaconitine^22^  Talatizamine^22^  Isotalatizidine^22^  Neoline^22^  Fuziline^22^  Isodelphinine^22^  Benzoylmesaconine^22^  Senbusine A^22^  Hokbusine A^22^  Benzoylaconine^22^  Benzoylhypaconine^22^  Neojiangyouaconitine^22^  Aldohypaconitine^22^  Deoxyaconitine^22^  Beiwutine^22^  Aconifine^22^  Aconine^22^  Yunaconitine^22^  Chasmanine^22^  Foresticine^22^  N-deethylaconine^22^  Beiwutinine^22^  Hypaconine^22^  Mesaconine^22^ | [72303](https://pubchem.ncbi.nlm.nih.gov/compound/72303)  [73399](https://pubchem.ncbi.nlm.nih.gov/compound/73399)  [338](https://pubchem.ncbi.nlm.nih.gov/compound/338)  [637542](https://pubchem.ncbi.nlm.nih.gov/compound/637542)  [71456946](https://pubchem.ncbi.nlm.nih.gov/compound/71456946)  [441742](https://pubchem.ncbi.nlm.nih.gov/compound/441742)  [245005](https://pubchem.ncbi.nlm.nih.gov/compound/245005)  [441747](https://pubchem.ncbi.nlm.nih.gov/compound/441747)  [441737](https://pubchem.ncbi.nlm.nih.gov/compound/441737)  [441761](https://pubchem.ncbi.nlm.nih.gov/compound/441761)  [3084020](https://pubchem.ncbi.nlm.nih.gov/compound/3084020)  [120682](https://pubchem.ncbi.nlm.nih.gov/compound/120682)  [14163819](https://pubchem.ncbi.nlm.nih.gov/compound/14163819)  [102146471](https://pubchem.ncbi.nlm.nih.gov/compound/102146471)  [24832659](https://pubchem.ncbi.nlm.nih.gov/compound/24832659)  [158048](https://pubchem.ncbi.nlm.nih.gov/compound/158048)  [24832661](https://pubchem.ncbi.nlm.nih.gov/compound/24832661)  [20055771](https://pubchem.ncbi.nlm.nih.gov/compound/20055771)  [78358526](https://pubchem.ncbi.nlm.nih.gov/compound/78358526)  [78358553](https://pubchem.ncbi.nlm.nih.gov/compound/78358553)  [5317220](https://pubchem.ncbi.nlm.nih.gov/compound/5317220)  [21598997](https://pubchem.ncbi.nlm.nih.gov/compound/21598997)  [21627927](https://pubchem.ncbi.nlm.nih.gov/compound/21627927)  [441705](https://pubchem.ncbi.nlm.nih.gov/compound/441705)  [20054813](https://pubchem.ncbi.nlm.nih.gov/compound/20054813)  [155569](https://pubchem.ncbi.nlm.nih.gov/compound/155569)  [20055812](https://pubchem.ncbi.nlm.nih.gov/compound/20055812)  [154723942](https://pubchem.ncbi.nlm.nih.gov/compound/154723942)  [101552717](https://pubchem.ncbi.nlm.nih.gov/compound/101552717)  [101552718](https://pubchem.ncbi.nlm.nih.gov/compound/101552718)  [101671038](https://pubchem.ncbi.nlm.nih.gov/compound/101671038)  [76189547](https://pubchem.ncbi.nlm.nih.gov/compound/76189547) |
|  |  | Oxonitine^22^  Guiwuline^22^  Penduline^22^  Ignavine^22^  Delgradine^22^  14-O-Anisoylneoline^22^  14-O-Acetyneoline^22^  Foresaconitine^22^  Crassicauline A^22^  Hetisine^22^  Songoramine^22^  12-Epinapelline^22^  Aconicarchamine A^22^  Aconicarchamine B^22^  Napelline^22^  Carmichaeline A^22^  Yokonoside^22^  Higenamine^22^  Salsolinol^22^  Fuzitine^22^  Oleracein E^22^  16beta-Hydroxycardiopetaline^22^  Columbianine^22^  Aconitamide^22^  6”-O-Acetylliquiritin^22^  Liquiritigenin^22^  Isoliquiritigenin^22^  Liquiritin^22^  Gracillin^22^  Glyceryl monopalmitate^22^  Fuzinoside^22^ | [6708531](https://pubchem.ncbi.nlm.nih.gov/compound/6708531)  [70688216](https://pubchem.ncbi.nlm.nih.gov/compound/70688216)  [179472](https://pubchem.ncbi.nlm.nih.gov/compound/179472)  [71448929](https://pubchem.ncbi.nlm.nih.gov/compound/71448929)  [139075130](https://pubchem.ncbi.nlm.nih.gov/compound/139075130)  [12068477](https://pubchem.ncbi.nlm.nih.gov/compound/12068477)  [14312993](https://pubchem.ncbi.nlm.nih.gov/compound/14312993)  [20055981](https://pubchem.ncbi.nlm.nih.gov/compound/20055981)  [157539](https://pubchem.ncbi.nlm.nih.gov/compound/157539)  [431673](https://pubchem.ncbi.nlm.nih.gov/compound/431673)  [14526618](https://pubchem.ncbi.nlm.nih.gov/compound/14526618)  [3133561](https://pubchem.ncbi.nlm.nih.gov/compound/3133561)  [102484827](https://pubchem.ncbi.nlm.nih.gov/compound/102484827)  [102484828](https://pubchem.ncbi.nlm.nih.gov/compound/102484828)  [441749](https://pubchem.ncbi.nlm.nih.gov/compound/441749)  [102144179](https://pubchem.ncbi.nlm.nih.gov/compound/102144179)  [3085046](https://pubchem.ncbi.nlm.nih.gov/compound/3085046)  [114840](https://pubchem.ncbi.nlm.nih.gov/compound/114840)  [91588](https://pubchem.ncbi.nlm.nih.gov/compound/91588)  [72201752](https://pubchem.ncbi.nlm.nih.gov/compound/72201752)  [21574476](https://pubchem.ncbi.nlm.nih.gov/compound/21574476)  [102511299](https://pubchem.ncbi.nlm.nih.gov/compound/102511299)  [101457306](https://pubchem.ncbi.nlm.nih.gov/compound/101457306)  [15944660](https://pubchem.ncbi.nlm.nih.gov/compound/15944660)  [101051311](https://pubchem.ncbi.nlm.nih.gov/compound/101051311)  [114829](https://pubchem.ncbi.nlm.nih.gov/compound/114829)  [638278](https://pubchem.ncbi.nlm.nih.gov/compound/638278)  [503737](https://pubchem.ncbi.nlm.nih.gov/compound/503737)  [159861](https://pubchem.ncbi.nlm.nih.gov/compound/159861)  [3084463](https://pubchem.ncbi.nlm.nih.gov/compound/3084463)  [102339571](https://pubchem.ncbi.nlm.nih.gov/compound/102339571) |
| 20. | *Salvia miltiorrhiza*  (55) | Danshensu^23^  protocatechuic aldehyde^23^  lithospermic acid^23^  tanshinone IIB^23^  dihydrotanshinone I^23^   tanshinone I^23^  miltiradiene^23^  isoquercitrin^23^ | [11600642](https://pubchem.ncbi.nlm.nih.gov/compound/11600642)  [8768](https://pubchem.ncbi.nlm.nih.gov/compound/8768)  [6441498](https://pubchem.ncbi.nlm.nih.gov/compound/6441498)  [9926694](https://pubchem.ncbi.nlm.nih.gov/compound/9926694)  [11425923](https://pubchem.ncbi.nlm.nih.gov/compound/11425923)  [114917](https://pubchem.ncbi.nlm.nih.gov/compound/114917)  [20837867](https://pubchem.ncbi.nlm.nih.gov/compound/20837867)  [5280804](https://pubchem.ncbi.nlm.nih.gov/compound/5280804) |
|  |  | salvianolic acid A^24^  salvianolic acid B^24^  salvianolic acid C^24^  salvianolic acid D^24^  salvianolic acid E^24^  salvianolic acid G^24^  methyl rosmarinate^24^  dimethyl lithospermate^24^  lithospermic acid B^24^  ursolic acid^24^  tigogenin^24^  tanshinone IIA^24^  tanshinone VI^24^  cryptotanshinone^24^  isotanshinone I^24^  isotanshinone II^24^  isotanshinone IIB^24^  isocryptotanshinone^24^  hydroxytanshinone IIA^24^  methyl tanshinonate^24^  danshenxinkun B^24^  danshenxinkun C^24^  danshenxinkun D^24^  dihydroisotanshinone I^24^  neocryptotanshinone^24^  deoxyneocryptotanshinone^24^ | [5281793](https://pubchem.ncbi.nlm.nih.gov/compound/5281793)  [11629084](https://pubchem.ncbi.nlm.nih.gov/compound/11629084)  [13991590](https://pubchem.ncbi.nlm.nih.gov/compound/13991590)  [75412558](https://pubchem.ncbi.nlm.nih.gov/compound/75412558)  [86278266](https://pubchem.ncbi.nlm.nih.gov/compound/86278266)  [11530200](https://pubchem.ncbi.nlm.nih.gov/compound/11530200)  [6479915](https://pubchem.ncbi.nlm.nih.gov/compound/6479915)  [70688393](https://pubchem.ncbi.nlm.nih.gov/compound/70688393)  [6451084](https://pubchem.ncbi.nlm.nih.gov/compound/6451084)  [64945](https://pubchem.ncbi.nlm.nih.gov/compound/64945)  [99516](https://pubchem.ncbi.nlm.nih.gov/compound/99516)  [164676](https://pubchem.ncbi.nlm.nih.gov/compound/164676)  [149138](https://pubchem.ncbi.nlm.nih.gov/compound/149138)  [160254](https://pubchem.ncbi.nlm.nih.gov/compound/160254)  [623940](https://pubchem.ncbi.nlm.nih.gov/compound/623940)  [44425166](https://pubchem.ncbi.nlm.nih.gov/compound/44425166)  [184102](https://pubchem.ncbi.nlm.nih.gov/compound/184102)  [626608](https://pubchem.ncbi.nlm.nih.gov/compound/626608)  [5318349](https://pubchem.ncbi.nlm.nih.gov/compound/5318349)  [14610613](https://pubchem.ncbi.nlm.nih.gov/compound/14610613)  [5320113](https://pubchem.ncbi.nlm.nih.gov/compound/5320113)  [5320114](https://pubchem.ncbi.nlm.nih.gov/compound/5320114)  [127172](https://pubchem.ncbi.nlm.nih.gov/compound/127172)  [89406](https://pubchem.ncbi.nlm.nih.gov/compound/89406)  [389888](https://pubchem.ncbi.nlm.nih.gov/compound/389888)  [15690458](https://pubchem.ncbi.nlm.nih.gov/compound/15690458) |
|  |  | salviol^24^  nortanshinone^24^  Tanshindiol A^24^  Tanshindiol B^24^  Tanshindiol C^24^  Miltirone^24^  1,2-Dihydrotanshinqiunone^24^  Ferruginol^24^  4-methylenemiltirone^24^  Tanshinlactone^24^  danshinspiroketallactone^24^  Epidanshenspiroketallactone^24^  cryptoacetalide^24^  miltiodiol^24^  miltipolone^24^  norsalvioxide^24^  2-isopropyl-8-methylphenanthrene-3,4-dione^24^ | [13966146](https://pubchem.ncbi.nlm.nih.gov/compound/13966146)  [10062187](https://pubchem.ncbi.nlm.nih.gov/compound/10062187)  [16730071](https://pubchem.ncbi.nlm.nih.gov/compound/16730071)  [5321620](https://pubchem.ncbi.nlm.nih.gov/compound/5321620)  [126072](https://pubchem.ncbi.nlm.nih.gov/compound/126072)  [160142](https://pubchem.ncbi.nlm.nih.gov/compound/160142)  [105119](https://pubchem.ncbi.nlm.nih.gov/compound/105119)  [442027](https://pubchem.ncbi.nlm.nih.gov/compound/442027)  [14609851](https://pubchem.ncbi.nlm.nih.gov/compound/14609851)  [5321617](https://pubchem.ncbi.nlm.nih.gov/compound/5321617)  [5316298](https://pubchem.ncbi.nlm.nih.gov/compound/5316298)  [102004791](https://pubchem.ncbi.nlm.nih.gov/compound/102004791)  [46896125](https://pubchem.ncbi.nlm.nih.gov/compound/46896125)  [11011966](https://pubchem.ncbi.nlm.nih.gov/compound/11011966)  [10086184](https://pubchem.ncbi.nlm.nih.gov/compound/10086184)  [14139391](https://pubchem.ncbi.nlm.nih.gov/compound/14139391)  [135872](https://pubchem.ncbi.nlm.nih.gov/compound/135872) |
|  |  | Dihydrotanshinone^25^  Neosalvianen^25^  Salvianen^25^  Salviadione^25^ | [5316743](https://pubchem.ncbi.nlm.nih.gov/compound/5316743)  [11472648](https://pubchem.ncbi.nlm.nih.gov/compound/11472648)  [11186248](https://pubchem.ncbi.nlm.nih.gov/compound/11186248)  [135442608](https://pubchem.ncbi.nlm.nih.gov/compound/135442608) |

**References:**

1. Huyen, C. T. T. *et al.* Chemical Constituents from Cimicifuga dahurica and Their Anti-Proliferative Effects on MCF-7 Breast Cancer Cells. *Molecules* **23**, (2018).

2. Guo, Y. *et al.* Traditional uses, phytochemistry, pharmacology and toxicology of the genus Cimicifuga: A review. *J. Ethnopharmacol.* **209**, 264–282 (2017).

3. Qin, R. L. *et al.* Assessment of phenolics contents and antioxidant properties in Cimicifuga dahurica (Turcz.) Maxim during drying process. *Ind. Crops Prod.* **107**, 288–296 (2017).

4. Tran, H. N. K. *et al.* Anti-inflammatory activity of compounds from the rhizome of Cnidium officinale. *Arch. Pharm. Res.* **41**, 977–985 (2018).

5. Deng, G.-G. *et al.* Chemical constituents from lipophilic parts in roots of Angelica dahurica var. Formosana cv. Chuanbaizhi. *Zhongguo Zhongyao Zazhi* **40**, 2148–2156 (2015).

6. Li, D. & Wu, L. Coumarins from the roots of angelica dahurica cause anti‑allergic inflammation. *Exp. Ther. Med.* **14**, 874–880 (2017).

7. Krizevski, R. *et al.* Composition and stereochemistry of ephedrine alkaloids accumulation in Ephedra sinica Stapf. *Phytochemistry* **71**, 895–903 (2010).

8. Zang, X. *et al.* A-type proanthocyanidins from the stems of Ephedra sinica (Ephedraceae) and their antimicrobial activities. *Molecules* **18**, 5172–5189 (2013).

9. ZHANG, B. M. *et al.* Phytochemistry and pharmacology of genus Ephedra. *Chin. J. Nat. Med.* **16**, 811–828 (2018).

10. Sugimoto, S., Nakamura, S., Matsuda, H., Kitagawa, N. & Yoshikawa, M. Chemical constituents from seeds of panax ginseng: structure of new dammarane-type triterpene ketone, panaxadione, and HPLC comparisons of seeds and flesh. *Chem. Pharm. Bull.* **57**, 283–287 (2009).

11. P.hou, J. The Chemical Constituents of Ginseng Plants. *Am. J. Chin. Med.* **05**, (2012).

12. Ali, B. H., Blunden, G., Tanira, M. O. & Nemmar, A. Some phytochemical, pharmacological and toxicological properties of ginger (Zingiber officinale Roscoe): A review of recent research. *Food Chem. Toxicol.* **46**, 409–420 (2008).

13. Ashraf, K., Sultan, S. & Shah, S. A. A. Phychemistry, Phytochemical, Pharmacological and Molecular Study of Zingiber Officinale Roscoe: a Review. *Int. J. Pharm. Pharm. Sci.* **9**, 8 (2017).

14. Sharifi-Rad, M. *et al.* Plants of the genus zingiber as a source of bioactive phytochemicals: From tradition to pharmacy. *Molecules* **22**, 1–20 (2017).

15. Yang, P. F. *et al.* Phytochemical and chemotaxonomic study of Poria cocos (Schw.) Wolf. *Biochem. Syst. Ecol.* **83**, 54–56 (2019).

16. Ríos, J. L. Chemical constituents and pharmacological properties of poria cocos. *Planta Med.* **77**, 681–691 (2011).

17. Li, S. S. *et al.* Phytochemical variation among the traditional Chinese medicine Mu Dan Pi from Paeonia suffruticosa (tree peony). *Phytochemistry* **146**, 16–24 (2018).

18. He, C. *et al.* Chemical constituents from seeds of Paeonia suffruticosa. *Zhongguo Zhong Yao Za Zhi* **35**, 1428–1431 (2010).

19. Zhou, Y. *et al.* Chemical constituents, antibacterial activity and mechanism of Paeonia suffruticosa Andr. buds extract against Staphylococcus aureus and Escherichia coli O157:H7. *Nat. Prod. Res.* **35**, 1005–1009 (2021).

20. Wang, Z., He, C., Peng, Y., Chen, F. & Xiao, P. Origins, phytochemistry, pharmacology, analytical methods and safety of cortex moutan (paeonia suffruticosa Andrew): A systematic review. *Molecules* **22**, (2017).

21. Zhang, J. *et al.* [Chemical constituents of lateral roots of Aconitum carmichaelii Debx]. *Yao Xue Xue Bao* **49**, 1150–1154 (2014).

22. Zhou, G. *et al.* A review on phytochemistry and pharmacological activities of the processed lateral root of Aconitum carmichaelii Debeaux. *J. Ethnopharmacol.* **160**, 173–193 (2015).

23. Zeng, H. *et al.* Comparative analysis of the major chemical constituents in Salvia miltiorrhiza roots, stems, leaves and flowers during different growth periods by UPLC-TQ-MS/MS and HPLC-ELSD methods. *Molecules* **22**, 1–16 (2017).

24. Wang, B. Q. Salvia miltiorrhiza chemical and pharmacological review of a medicinal plant. *J. Med. Plants Res.* **4**, 2813–2820 (2010).

25. Jassbi, A. R., Zare, S., Firuzi, O. & Xiao, J. Bioactive phytochemicals from shoots and roots of Salvia species. *Phytochem. Rev.* **15**, 829–867 (2016).
