# Supplementary material for: An in silico approach to develop potential therapies against Middle East Respiratory Syndrome Coronavirus (MERS-CoV)
Source: Heliyon. 2024 Feb 9;10(4):e25837. doi: 10.1016/j.heliyon.2024.e25837 (PMC10877303; doi:10.1016/j.heliyon.2024.e25837)
Supplement: Multimedia component 3 [file mmc3.docx]

| **SL.**  **No.** | **Plant Name** | **Compounds** | **CID** |
| --- | --- | --- | --- |
| 21. | *Citrus unshiu*  (25) | Hesperidin^1^  Naringin^1^  Neohesperidin^1^  Naringenin^1^  Hesperetin^1^  *p*-coumaric acid^2^  sinapic acid^2^  protocatechuic acid^2^  *p*-hydroxybenzoic acid^2^  vanillic acid^2^  narirutin^2^ | [10621](https://pubchem.ncbi.nlm.nih.gov/compound/10621)  [442428](https://pubchem.ncbi.nlm.nih.gov/compound/442428)  [442439](https://pubchem.ncbi.nlm.nih.gov/compound/442439)  [932](https://pubchem.ncbi.nlm.nih.gov/compound/932)  [72281](https://pubchem.ncbi.nlm.nih.gov/compound/72281)  [637542](https://pubchem.ncbi.nlm.nih.gov/compound/637542)  [637775](https://pubchem.ncbi.nlm.nih.gov/compound/637775)  [72](https://pubchem.ncbi.nlm.nih.gov/compound/72)  [135](https://pubchem.ncbi.nlm.nih.gov/compound/135)  [8468](https://pubchem.ncbi.nlm.nih.gov/compound/8468)  [442431](https://pubchem.ncbi.nlm.nih.gov/compound/442431) |
|  |  | Nobiletin (3′,4′,5,6,7,8-hexamethoxyflavone)^3^  kaempferol 3-O-rutinoside^3^  limocitrin 3-glucoside^3^  didymin (4′-methoxyl naringenin 7- O-rutinoside)^3^  naringenin 7-Orutinoside^3^  Hesperetin-7-O-glucoside^4^  Prunin^4^  Sinensetin^4^  Tangeretin^4^  γ-terpinene^5^  2-β-pinene^5^  1-methyl-2-isopropylbenzene^5^  L-limonene^5^  β-ocimene^5^ | [72344](https://pubchem.ncbi.nlm.nih.gov/compound/72344)  [5318767](https://pubchem.ncbi.nlm.nih.gov/compound/5318767)  [44259988](https://pubchem.ncbi.nlm.nih.gov/compound/44259988)  [16760075](https://pubchem.ncbi.nlm.nih.gov/compound/16760075)  [85704](https://pubchem.ncbi.nlm.nih.gov/compound/85704)  [20111686](https://pubchem.ncbi.nlm.nih.gov/compound/20111686)  [92794](https://pubchem.ncbi.nlm.nih.gov/compound/92794)  [145659](https://pubchem.ncbi.nlm.nih.gov/compound/145659)  [68077](https://pubchem.ncbi.nlm.nih.gov/compound/68077)  [7461](https://pubchem.ncbi.nlm.nih.gov/compound/7461)  [6654](https://pubchem.ncbi.nlm.nih.gov/compound/6654)  [10703](https://pubchem.ncbi.nlm.nih.gov/compound/10703)  [439250](https://pubchem.ncbi.nlm.nih.gov/compound/439250)  [5281553](https://pubchem.ncbi.nlm.nih.gov/compound/5281553) |
| 22. | *Gardenia jasminoides Ellis*  (70) | geniposidic acid^6^  chlorogenic acid^6^  genipin-1-β-gentiobioside^6^  geniposide^6^  genipin^6^  crocin-1^6^  crocin-2^6^  jasminoside I^6^  gardenoside^6^  gardaloside^6^  5, 7, 3’, 4’, 5’-pentamethoxyflavone^6^  shikimic acid^6^  1, 2, 4-benzenetriol^6^  3, 4-dimethoxy-benzoic acid^6^  dibutyl phthalate^6^  diisobutyl phthalate^6^  α-gardiol^6^  β-gardiol^6^  genameside C^6^  deacetylasperulosidic acid^6^  syringic acid^6^  syringaldehyde^6^  vanillic acid^6^  3-hydroxy-vanillic acid^6^  3, 4, 5-trimethoxy-phenol^6^  4-methoxy-benzaldehyde^6^  7-hydroxy-5-methoxy-chromone^6^  5, 7, 3’-trihydroxy-6, 4’, 5’-trimethoxyflavone^6^ | [443354](https://pubchem.ncbi.nlm.nih.gov/compound/443354)  [1794427](https://pubchem.ncbi.nlm.nih.gov/compound/1794427)  [14179128](https://pubchem.ncbi.nlm.nih.gov/compound/14179128)  [107848](https://pubchem.ncbi.nlm.nih.gov/compound/107848)  [442424](https://pubchem.ncbi.nlm.nih.gov/compound/442424)  [5281233](https://pubchem.ncbi.nlm.nih.gov/compound/5281233)  [9940690](https://pubchem.ncbi.nlm.nih.gov/compound/9940690)  [102596097](https://pubchem.ncbi.nlm.nih.gov/compound/102596097)  [24721095](https://pubchem.ncbi.nlm.nih.gov/compound/24721095)  [11631807](https://pubchem.ncbi.nlm.nih.gov/compound/11631807)  [16044716](https://pubchem.ncbi.nlm.nih.gov/compound/16044716)  [8742](https://pubchem.ncbi.nlm.nih.gov/compound/8742)  [10787](https://pubchem.ncbi.nlm.nih.gov/compound/10787)  [86584921](https://pubchem.ncbi.nlm.nih.gov/compound/86584921)  [3026](https://pubchem.ncbi.nlm.nih.gov/compound/3026)  [6782](https://pubchem.ncbi.nlm.nih.gov/compound/6782)  [101936008](https://pubchem.ncbi.nlm.nih.gov/compound/101936008)  [57340454](https://pubchem.ncbi.nlm.nih.gov/compound/57340454)  [11692460](https://pubchem.ncbi.nlm.nih.gov/compound/11692460)  [12315350](https://pubchem.ncbi.nlm.nih.gov/compound/12315350)  [10742](https://pubchem.ncbi.nlm.nih.gov/compound/10742)  [8655](https://pubchem.ncbi.nlm.nih.gov/compound/8655)  [8468](https://pubchem.ncbi.nlm.nih.gov/compound/8468)  [129848169](https://pubchem.ncbi.nlm.nih.gov/compound/129848169)  [69505](https://pubchem.ncbi.nlm.nih.gov/compound/69505)  [31244](https://pubchem.ncbi.nlm.nih.gov/compound/31244)  [129848159](https://pubchem.ncbi.nlm.nih.gov/compound/129848159)  [5496475](https://pubchem.ncbi.nlm.nih.gov/compound/5496475) |
|  |  | 3beta,23- dihydroxyurs-12-en-28-oic acid^6^  emodin^6^  physcion^6^  stearic acid^6^  Gardenal^6^  6-α-hydroxy geniposide^6^  Feretoside^6^  shanzhiside^6^  lamalbidic acid^6^  picrocrocinic acid^6^  jasminoside A^6^  epijasminoside A^6^  jasminoside R^6^  jasminoside S^6^  jasminoside T^6^ | [14136881](https://pubchem.ncbi.nlm.nih.gov/compound/14136881)  [3220](https://pubchem.ncbi.nlm.nih.gov/compound/3220)  [10639](https://pubchem.ncbi.nlm.nih.gov/compound/10639)  [5281](https://pubchem.ncbi.nlm.nih.gov/compound/5281)  [4763](https://pubchem.ncbi.nlm.nih.gov/compound/4763)  [6325021](https://pubchem.ncbi.nlm.nih.gov/compound/6325021)  [442433](https://pubchem.ncbi.nlm.nih.gov/compound/442433)  [11948668](https://pubchem.ncbi.nlm.nih.gov/compound/11948668)  [101434718](https://pubchem.ncbi.nlm.nih.gov/compound/101434718)  [5320582](https://pubchem.ncbi.nlm.nih.gov/compound/5320582)  [98050833](https://pubchem.ncbi.nlm.nih.gov/compound/98050833)  [76551288](https://pubchem.ncbi.nlm.nih.gov/compound/76551288)  [71552546](https://pubchem.ncbi.nlm.nih.gov/compound/71552546)  [71552547](https://pubchem.ncbi.nlm.nih.gov/compound/71552547)  [71552548](https://pubchem.ncbi.nlm.nih.gov/compound/71552548) |
|  |  | chikusetsusaponin IVa methyl ester^6^  chikusetsusaponin IVa butyl ester^6^  protocatechuic acid^6^  genipin-gentiobioside^6^  7alpha-hydroxy sitosterol^6^  z-3-hexenyl tiglate^6^  trans-beta-ocimene^6^  10-O-succinoylgeniposide^6^  6’-O-acetylgeniposide^6^  10-O-acetylgeniposide^6^  Jasminodiol^6^  jasminoside H^6^  imperatorin^6^  isoimperatorin^6^  crocetin^6^  sudan III^6^  crocin-3^6^ | [637855](https://pubchem.ncbi.nlm.nih.gov/compound/637855)  [44566502](https://pubchem.ncbi.nlm.nih.gov/compound/44566502)  [72](https://pubchem.ncbi.nlm.nih.gov/compound/72)  [3082301](https://pubchem.ncbi.nlm.nih.gov/compound/3082301)  [146158661](https://pubchem.ncbi.nlm.nih.gov/compound/146158661)  [5352469](https://pubchem.ncbi.nlm.nih.gov/compound/5352469)  [5281553](https://pubchem.ncbi.nlm.nih.gov/compound/5281553)  [44255239](https://pubchem.ncbi.nlm.nih.gov/compound/44255239)  [44253991](https://pubchem.ncbi.nlm.nih.gov/compound/44253991)  [6324916](https://pubchem.ncbi.nlm.nih.gov/compound/6324916)  [24896698](https://pubchem.ncbi.nlm.nih.gov/compound/24896698)  [102596096](https://pubchem.ncbi.nlm.nih.gov/compound/102596096)  [10212](https://pubchem.ncbi.nlm.nih.gov/compound/10212)  [68081](https://pubchem.ncbi.nlm.nih.gov/compound/68081)  [5281232](https://pubchem.ncbi.nlm.nih.gov/compound/5281232)  [62331](https://pubchem.ncbi.nlm.nih.gov/compound/62331)  [10461942](https://pubchem.ncbi.nlm.nih.gov/compound/10461942) |
|  |  | methyl 5-O-caffeoyl-3-O-sinapoylquinate^6^  ethyl 5-O-caffeoyl-3-O-sinapoylquinate^6^  methyl 5-O-caffeoyl-4- O-sinapoylquinate^6^  ethyl 5-O-caffeoyl-4-O-sinapoylquinate^6^  methyl 3,5-di-O-caffeoyl-4-O-(3- hydroxy-3-methyl) glutaroylquinate^6^  Ixoroside^6^  8-epiapodantheroside^6^  gardenate A^6^  2-hydroxyethylgardenamide A^6^  Jasminoside F^6^ | [11671431](https://pubchem.ncbi.nlm.nih.gov/compound/11671431)  [11699888](https://pubchem.ncbi.nlm.nih.gov/compound/11699888)  [11635556](https://pubchem.ncbi.nlm.nih.gov/compound/11635556)  [11512664](https://pubchem.ncbi.nlm.nih.gov/compound/11512664)  [11693219](https://pubchem.ncbi.nlm.nih.gov/compound/11693219)  [44566558](https://pubchem.ncbi.nlm.nih.gov/compound/44566558)  [11372643](https://pubchem.ncbi.nlm.nih.gov/compound/11372643)  [10611205](https://pubchem.ncbi.nlm.nih.gov/compound/10611205)  [10825707](https://pubchem.ncbi.nlm.nih.gov/compound/10825707)  [10807517](https://pubchem.ncbi.nlm.nih.gov/compound/10807517) |
| 23. | *Gentiana scabra Bunge*  (15) | β-amyrin^7^  β-amyrin acetate^7^  uvaol^7^  gentiopicroside^8^  6beta-hydroxyswertiajaposide A^8^  Gelidoside^8^  Trifloroside^8^  Scabraside^8^  Durvillonol^9^  masilinic acid^9^  urjinolic acid^9^  3beta-erythrodiol^9^  corosolic acid^9^  pygenic acid C^9^  chiratenol^9^ | [225687](https://pubchem.ncbi.nlm.nih.gov/compound/225687)  [345510](https://pubchem.ncbi.nlm.nih.gov/compound/345510)  [92802](https://pubchem.ncbi.nlm.nih.gov/compound/92802)  [88708](https://pubchem.ncbi.nlm.nih.gov/compound/88708)  [11153922](https://pubchem.ncbi.nlm.nih.gov/compound/11153922)  [46174003](https://pubchem.ncbi.nlm.nih.gov/compound/46174003)  [101688128](https://pubchem.ncbi.nlm.nih.gov/compound/101688128)  [159134](https://pubchem.ncbi.nlm.nih.gov/compound/159134)  [634965](https://pubchem.ncbi.nlm.nih.gov/compound/634965)  [73659](https://pubchem.ncbi.nlm.nih.gov/compound/73659)  [146156259](https://pubchem.ncbi.nlm.nih.gov/compound/146156259)  [101761](https://pubchem.ncbi.nlm.nih.gov/compound/101761)  [6918774](https://pubchem.ncbi.nlm.nih.gov/compound/6918774)  [69049736](https://pubchem.ncbi.nlm.nih.gov/compound/69049736)  [14831162](https://pubchem.ncbi.nlm.nih.gov/compound/14831162) |
| 24. | *Magnoliae Officinalis Cortex*  (105) | Magnolol^10^  Honokiol^10^  O-methylhonokiol^10^  Piperitylmagnolol^10^  Dipiperitylmagnolol^10^  Piperitylhonokiol^10^  Bornylmagnolol^10^  Eudesmagnolol^10^  Eudeshonokiol A^10^  Eudeshonokiol B^10^  Clovanemagnolol^10^  Caryolanemagnolol^10^  Magnolignan A^10^  Magnolignan B^10^  Magnolignan C^10^  Magnolignan D^10^  Magnolignan A−2-O-beta-D-glucopyranoside^10^  Randainal/Magnaldehyde B^10^  Randaiol^10^  Magnatriol B^10^  Magnaldehyde D^10^ | [72300](https://pubchem.ncbi.nlm.nih.gov/compound/72300)  [72303](https://pubchem.ncbi.nlm.nih.gov/compound/72303)  [155160](https://pubchem.ncbi.nlm.nih.gov/compound/155160)  [11732171](https://pubchem.ncbi.nlm.nih.gov/compound/11732171)  [101612410](https://pubchem.ncbi.nlm.nih.gov/compound/101612410)  [13337242](https://pubchem.ncbi.nlm.nih.gov/compound/13337242)  [13337239](https://pubchem.ncbi.nlm.nih.gov/compound/13337239)  [14587417](https://pubchem.ncbi.nlm.nih.gov/compound/14587417)  [14587421](https://pubchem.ncbi.nlm.nih.gov/compound/14587421)  [21726646](https://pubchem.ncbi.nlm.nih.gov/compound/21726646)  [10323149](https://pubchem.ncbi.nlm.nih.gov/compound/10323149)  [21726647](https://pubchem.ncbi.nlm.nih.gov/compound/21726647)  [5319201](https://pubchem.ncbi.nlm.nih.gov/compound/5319201)  [5319202](https://pubchem.ncbi.nlm.nih.gov/compound/5319202)  [5319203](https://pubchem.ncbi.nlm.nih.gov/compound/5319203)  [5319204](https://pubchem.ncbi.nlm.nih.gov/compound/5319204)  [71720154](https://pubchem.ncbi.nlm.nih.gov/compound/71720154)  [5320888](https://pubchem.ncbi.nlm.nih.gov/compound/5320888)  [13337243](https://pubchem.ncbi.nlm.nih.gov/compound/13337243)  [5319191](https://pubchem.ncbi.nlm.nih.gov/compound/5319191)  [5319189](https://pubchem.ncbi.nlm.nih.gov/compound/5319189) |
|  |  | Magnaldehyde E^10^  4′-methoxymagnaldehyde B^10^  4′-methoxymagnaldehyde E^10^  Magnolignan E^10^  Coumanolignan^10^  Magnaldehyde C^10^  4′-methoxymagndialdehyde^10^  Manglieside D^10^  Magnolignan F^10^  Magnolignan I^10^  Isomagnolol^10^  Obovatol^10^  Eudesobovatol A^10^  Eudesobovatol B^10^  Obovatal^10^ | [5319190](https://pubchem.ncbi.nlm.nih.gov/compound/5319190)  [23657448](https://pubchem.ncbi.nlm.nih.gov/compound/23657448)  [23657447](https://pubchem.ncbi.nlm.nih.gov/compound/23657447)  [15714551](https://pubchem.ncbi.nlm.nih.gov/compound/15714551)  [24796112](https://pubchem.ncbi.nlm.nih.gov/compound/24796112)  [5319188](https://pubchem.ncbi.nlm.nih.gov/compound/5319188)  [23634517](https://pubchem.ncbi.nlm.nih.gov/compound/23634517)  [102473624](https://pubchem.ncbi.nlm.nih.gov/compound/102473624)  [5319207](https://pubchem.ncbi.nlm.nih.gov/compound/5319207)  [373768](https://pubchem.ncbi.nlm.nih.gov/compound/373768)  [159137](https://pubchem.ncbi.nlm.nih.gov/compound/159137)  [100771](https://pubchem.ncbi.nlm.nih.gov/compound/100771)  [442837](https://pubchem.ncbi.nlm.nih.gov/compound/442837)  [10346031](https://pubchem.ncbi.nlm.nih.gov/compound/10346031)  [6439677](https://pubchem.ncbi.nlm.nih.gov/compound/6439677) |
|  |  | Magnolignan G^10^  Magnolignan H^10^  Icariside E5^10^  Lariciresinol^10^  (+)-Syringaresinol^10^  Pinoresinol−4-O-beta-D-glucopyranoside^10^  Magnoloside A^10^  Magnoloside B^10^  Magnoloside C^10^  Magnoloside D^10^  Crassifolioside^10^  Acteoside^10^  Tachioside^10^  Syringaldehyde^10^  Sinapic aldehyde^10^  Isosyringinoside^10^  Erigeside C^10^  Coniferaldehyde^10^  Coniferyl alcohol^10^  p-Coumaric acid^10^  Caffeic acid methyl ester^10^  Coniferin^10^  Magnolianone^10^  O-methyleugenol^10^  Chavicol^10^ | [15714552](https://pubchem.ncbi.nlm.nih.gov/compound/15714552)  [373767](https://pubchem.ncbi.nlm.nih.gov/compound/373767)  [91884923](https://pubchem.ncbi.nlm.nih.gov/compound/91884923)  [332427](https://pubchem.ncbi.nlm.nih.gov/compound/332427)  [443023](https://pubchem.ncbi.nlm.nih.gov/compound/443023)  [486614](https://pubchem.ncbi.nlm.nih.gov/compound/486614)  [21629882](https://pubchem.ncbi.nlm.nih.gov/compound/21629882)  [14018784](https://pubchem.ncbi.nlm.nih.gov/compound/14018784)  [14018786](https://pubchem.ncbi.nlm.nih.gov/compound/14018786)  [131676053](https://pubchem.ncbi.nlm.nih.gov/compound/131676053)  [102193658](https://pubchem.ncbi.nlm.nih.gov/compound/102193658)  [5281800](https://pubchem.ncbi.nlm.nih.gov/compound/5281800)  [11962143](https://pubchem.ncbi.nlm.nih.gov/compound/11962143)  [8655](https://pubchem.ncbi.nlm.nih.gov/compound/8655)  [5280802](https://pubchem.ncbi.nlm.nih.gov/compound/5280802)  [57399043](https://pubchem.ncbi.nlm.nih.gov/compound/57399043)  [14132346](https://pubchem.ncbi.nlm.nih.gov/compound/14132346)  [5280536](https://pubchem.ncbi.nlm.nih.gov/compound/5280536)  [1549095](https://pubchem.ncbi.nlm.nih.gov/compound/1549095)  [637542](https://pubchem.ncbi.nlm.nih.gov/compound/637542)  [689075](https://pubchem.ncbi.nlm.nih.gov/compound/689075)  [5280372](https://pubchem.ncbi.nlm.nih.gov/compound/5280372)  [16739270](https://pubchem.ncbi.nlm.nih.gov/compound/16739270)  [7127](https://pubchem.ncbi.nlm.nih.gov/compound/7127)  [68148](https://pubchem.ncbi.nlm.nih.gov/compound/68148) |
|  |  | p-Hydroxybenzaldehyde^10^  Loliolide^10^  Blumenol A^10^  Blumenol B^10^  S-(+)-dehydrovomifoliol^10^  Grasshopper ketone^10^  (S)-tembetarine^10^  Reticuline^10^  Magnoflorine^10^  (+)-Laurifoline^10^  (+)-Menisperine^10^  (+)-Xanthoplanine^10^  Asimilobine^10^  Lysicamine^10^  Roemerine^10^  Anonaine^10^  Anolobine^10^  Liriodenine^10^  Nornantenine^10^  Anaxagoreine^10^  N-nornuciferine^10^  10-demethylcryptaustoline^10^ | [126](https://pubchem.ncbi.nlm.nih.gov/compound/126)  [100332](https://pubchem.ncbi.nlm.nih.gov/compound/100332)  [5280462](https://pubchem.ncbi.nlm.nih.gov/compound/5280462)  [14135402](https://pubchem.ncbi.nlm.nih.gov/compound/14135402)  [688492](https://pubchem.ncbi.nlm.nih.gov/compound/688492)  [13922639](https://pubchem.ncbi.nlm.nih.gov/compound/13922639)  [167718](https://pubchem.ncbi.nlm.nih.gov/compound/167718)  [439653](https://pubchem.ncbi.nlm.nih.gov/compound/439653)  [73337](https://pubchem.ncbi.nlm.nih.gov/compound/73337)  [12305611](https://pubchem.ncbi.nlm.nih.gov/compound/12305611)  [30358](https://pubchem.ncbi.nlm.nih.gov/compound/30358)  [5315336](https://pubchem.ncbi.nlm.nih.gov/compound/5315336)  [160875](https://pubchem.ncbi.nlm.nih.gov/compound/160875)  [122691](https://pubchem.ncbi.nlm.nih.gov/compound/122691)  [119204](https://pubchem.ncbi.nlm.nih.gov/compound/119204)  [160597](https://pubchem.ncbi.nlm.nih.gov/compound/160597)  [164710](https://pubchem.ncbi.nlm.nih.gov/compound/164710)  [10144](https://pubchem.ncbi.nlm.nih.gov/compound/10144)  [3084228](https://pubchem.ncbi.nlm.nih.gov/compound/3084228)  [13891860](https://pubchem.ncbi.nlm.nih.gov/compound/13891860)  [12313579](https://pubchem.ncbi.nlm.nih.gov/compound/12313579)  [100969440](https://pubchem.ncbi.nlm.nih.gov/compound/100969440) |
|  |  | N-methylisosalsoline^10^  N-Feruloylputrescine(trans)^10^  Indole−3-aldehyde^10^  Betaine^10^  Limonene^10^  Caryophyllene^10^  Caryophyllene epoxide^10^  β- eudesmol^10^  γ- eudesmol^10^  Cryptomeridiol^10^  β-sitostenone^10^  Stigmasta− 4,22-dien− 3-one^10^  Hyperoside^10^  Afzelin^10^  Choerospondin^10^  Isorhamnetin− 3-O-β-D-glucoside^10^  Ethyl palmitate (Ethyl hexadecanoate)^10^  Ethyl stearate (Ethyl octadecanoate)^10^  Arachidic acid (Icosanoic acid)^10^  1-hexacosanol (hexacosan−1-ol)^10^  Palmitone (hentriacontan−16-one)^10^  Nonacosylic acid (Nonacosanoic acid)^10^ | [40091](https://pubchem.ncbi.nlm.nih.gov/compound/40091)  [92339985](https://pubchem.ncbi.nlm.nih.gov/compound/92339985)  [10256](https://pubchem.ncbi.nlm.nih.gov/compound/10256)  [247](https://pubchem.ncbi.nlm.nih.gov/compound/247)  [22311](https://pubchem.ncbi.nlm.nih.gov/compound/22311)  [5281515](https://pubchem.ncbi.nlm.nih.gov/compound/5281515)  [14350](https://pubchem.ncbi.nlm.nih.gov/compound/14350)  [91457](https://pubchem.ncbi.nlm.nih.gov/compound/91457)  [6432005](https://pubchem.ncbi.nlm.nih.gov/compound/6432005)  [165258](https://pubchem.ncbi.nlm.nih.gov/compound/165258)  [60123241](https://pubchem.ncbi.nlm.nih.gov/compound/60123241)  [6442194](https://pubchem.ncbi.nlm.nih.gov/compound/6442194)  [5281643](https://pubchem.ncbi.nlm.nih.gov/compound/5281643)  [5316673](https://pubchem.ncbi.nlm.nih.gov/compound/5316673)  [157745](https://pubchem.ncbi.nlm.nih.gov/compound/157745)  [5318645](https://pubchem.ncbi.nlm.nih.gov/compound/5318645)  [12366](https://pubchem.ncbi.nlm.nih.gov/compound/12366)  [8122](https://pubchem.ncbi.nlm.nih.gov/compound/8122)  [10467](https://pubchem.ncbi.nlm.nih.gov/compound/10467)  [68171](https://pubchem.ncbi.nlm.nih.gov/compound/68171)  [94741](https://pubchem.ncbi.nlm.nih.gov/compound/94741)  [20245](https://pubchem.ncbi.nlm.nih.gov/compound/20245) |
| 25. | *Ophiopogon japonicus*  (30) | glycoside C^11^  nolinospiroside F^11^  ophiopogonin B^11^  ophiopogonin D^11^  ophiopogonin A^11^  diosgenin^11^  ophiopogonin C’^11^  ophiopogonin D’^11^  ophiopogonin P^11^  ophiopogonin Q^11^  sprengerinin A^11^  cixiophiopogon A^11^  ophiopojaponin C^11^  ophiopogonin R^11^  ophiopogonin S^11^  prazerigenin A^11^ | [44566500](https://pubchem.ncbi.nlm.nih.gov/compound/44566500)  [86289919](https://pubchem.ncbi.nlm.nih.gov/compound/86289919)  [46173857](https://pubchem.ncbi.nlm.nih.gov/compound/46173857)  [46173859](https://pubchem.ncbi.nlm.nih.gov/compound/46173859)  [46173858](https://pubchem.ncbi.nlm.nih.gov/compound/46173858)  [99474](https://pubchem.ncbi.nlm.nih.gov/compound/99474)  [4483248](https://pubchem.ncbi.nlm.nih.gov/compound/4483248)  [10033524](https://pubchem.ncbi.nlm.nih.gov/compound/10033524)  [102195415](https://pubchem.ncbi.nlm.nih.gov/compound/102195415)  [71522132](https://pubchem.ncbi.nlm.nih.gov/compound/71522132)  [102081298](https://pubchem.ncbi.nlm.nih.gov/compound/102081298)  [102004869](https://pubchem.ncbi.nlm.nih.gov/compound/102004869)  [146160153](https://pubchem.ncbi.nlm.nih.gov/compound/146160153)  [71523896](https://pubchem.ncbi.nlm.nih.gov/compound/71523896)  [71523897](https://pubchem.ncbi.nlm.nih.gov/compound/71523897)  [13833781](https://pubchem.ncbi.nlm.nih.gov/compound/13833781) |
|  |  | methylophiopogonone B^11^  ophiopogonone B^11^  methylophiopogonone A^11^  6-aldehydo-isoophiopogonone A^11^  ophiopogonone A^11^  ophiopogonone C^11^  ophiopogonanone E^11^  methylophiopogonanone B^11^  methylophiopogonanone A^11^  ophiopogonanone A^11^  ophiopogonanone C^11^  Cryptomeridiol^12^  Pennogenin^12^  Sprengerinin C^12^ | [23259413](https://pubchem.ncbi.nlm.nih.gov/compound/23259413)  [14826840](https://pubchem.ncbi.nlm.nih.gov/compound/14826840)  [10065830](https://pubchem.ncbi.nlm.nih.gov/compound/10065830)  [5317207](https://pubchem.ncbi.nlm.nih.gov/compound/5317207)  [10087732](https://pubchem.ncbi.nlm.nih.gov/compound/10087732)  [11142766](https://pubchem.ncbi.nlm.nih.gov/compound/11142766)  [5316797](https://pubchem.ncbi.nlm.nih.gov/compound/5316797)  [46886723](https://pubchem.ncbi.nlm.nih.gov/compound/46886723)  [5319741](https://pubchem.ncbi.nlm.nih.gov/compound/5319741)  [9996586](https://pubchem.ncbi.nlm.nih.gov/compound/9996586)  [10871974](https://pubchem.ncbi.nlm.nih.gov/compound/10871974)  [165258](https://pubchem.ncbi.nlm.nih.gov/compound/165258)  [12314056](https://pubchem.ncbi.nlm.nih.gov/compound/12314056)  [44583957](https://pubchem.ncbi.nlm.nih.gov/compound/44583957) |
| 26. | *Perilla frutescens* L.  (193) | 3-Epicorosolic acid^13^  3-Epimaslinic acid^13^  Augustic acid^13^  Corosolic acid^13^  Hyptadienic acid^13^  Pomolic acid^13^  Tormentic acid^13^  Triacylglycerol^13^  Pentadecanoic acid^13^ | [15917998](https://pubchem.ncbi.nlm.nih.gov/compound/15917998)  [25564831](https://pubchem.ncbi.nlm.nih.gov/compound/25564831)  [15560128](https://pubchem.ncbi.nlm.nih.gov/compound/15560128)  [6918774](https://pubchem.ncbi.nlm.nih.gov/compound/6918774)  [14605533](https://pubchem.ncbi.nlm.nih.gov/compound/14605533)  [382831](https://pubchem.ncbi.nlm.nih.gov/compound/382831)  [73193](https://pubchem.ncbi.nlm.nih.gov/compound/73193)  [11146](https://pubchem.ncbi.nlm.nih.gov/compound/11146)  [13849](https://pubchem.ncbi.nlm.nih.gov/compound/13849) |
|  |  | Stearic acid^13^  β-Cholestanol^13^  Docosanol^13^  Eicosanol^13^  Heneicosanol^13^  Heptacosanol^13^  Hexacosanol^13^  Octacosanol^13^  Tetracosanol^13^  Triacontanol^13^  Tricosanol^13^  β-Tocopherol^13^  γ-Tocopherol^13^  δ-Tocopherol^13^  Rosmarinic acid methyl ester^13^ | [5281](https://pubchem.ncbi.nlm.nih.gov/compound/5281)  [6665](https://pubchem.ncbi.nlm.nih.gov/compound/6665)  [12620](https://pubchem.ncbi.nlm.nih.gov/compound/12620)  [12404](https://pubchem.ncbi.nlm.nih.gov/compound/12404)  [85014](https://pubchem.ncbi.nlm.nih.gov/compound/85014)  [74822](https://pubchem.ncbi.nlm.nih.gov/compound/74822)  [68171](https://pubchem.ncbi.nlm.nih.gov/compound/68171)  [68406](https://pubchem.ncbi.nlm.nih.gov/compound/68406)  [10472](https://pubchem.ncbi.nlm.nih.gov/compound/10472)  [68972](https://pubchem.ncbi.nlm.nih.gov/compound/68972)  [18431](https://pubchem.ncbi.nlm.nih.gov/compound/18431)  [6857447](https://pubchem.ncbi.nlm.nih.gov/compound/6857447)  [92729](https://pubchem.ncbi.nlm.nih.gov/compound/92729)  [92094](https://pubchem.ncbi.nlm.nih.gov/compound/92094)  [3012090](https://pubchem.ncbi.nlm.nih.gov/compound/3012090) |
|  |  | Apigenin 7-O-glucuronide^13^  Apigenin 7-O-diglucuronide^13^  Cimidahurinine^13^  Chrysoeriol^13^  Luteolin 7-O-diglucuronide^13^  Luteolin 7-O-glucoside^13^  Luteolin 7-O-glucuronide^13^  Luteolin-5-O-glucoside^13^  Scutellarein^13^  Chrysontenin^13^  Cyanin^13^  Malonylshisonin^13^  Shisonin^13^  (E,E)-α-Farnesene^13^  (Z)-3-Hexenyl acetate^13^  (Z,E)-α-Farnesene^13^  1,10-Decanediol^13^  1,2-Benzenedicarboxylic acid^13^  1,6-Cyclodecadiene^13^  10-Undecyn-1-ol  1-Cyclohexene-1-methanol^13^  1-Octen-3-ol^13^  2,2-Dimethylpentane^13^  2,4,6-Triisopropylphenol^13^  2,4-Hexadienal^13^ | [5319484](https://pubchem.ncbi.nlm.nih.gov/compound/5319484)  [126843388](https://pubchem.ncbi.nlm.nih.gov/compound/126843388)  [5315870](https://pubchem.ncbi.nlm.nih.gov/compound/5315870)  [5280666](https://pubchem.ncbi.nlm.nih.gov/compound/5280666)  [146036993](https://pubchem.ncbi.nlm.nih.gov/compound/146036993)  [5280637](https://pubchem.ncbi.nlm.nih.gov/compound/5280637)  [5280601](https://pubchem.ncbi.nlm.nih.gov/compound/5280601)  [5317471](https://pubchem.ncbi.nlm.nih.gov/compound/5317471)  [5281697](https://pubchem.ncbi.nlm.nih.gov/compound/5281697)  [197081](https://pubchem.ncbi.nlm.nih.gov/compound/197081)  [441688](https://pubchem.ncbi.nlm.nih.gov/compound/441688)  [11972402](https://pubchem.ncbi.nlm.nih.gov/compound/11972402)  [5282068](https://pubchem.ncbi.nlm.nih.gov/compound/5282068)  [5281516](https://pubchem.ncbi.nlm.nih.gov/compound/5281516)  [5363388](https://pubchem.ncbi.nlm.nih.gov/compound/5363388)  [5362889](https://pubchem.ncbi.nlm.nih.gov/compound/5362889)  [37153](https://pubchem.ncbi.nlm.nih.gov/compound/37153)  [1017](https://pubchem.ncbi.nlm.nih.gov/compound/1017)  [5365639](https://pubchem.ncbi.nlm.nih.gov/compound/5365639)  [76015](https://pubchem.ncbi.nlm.nih.gov/compound/76015)  [317542](https://pubchem.ncbi.nlm.nih.gov/compound/317542)  [18827](https://pubchem.ncbi.nlm.nih.gov/compound/18827)  [11542](https://pubchem.ncbi.nlm.nih.gov/compound/11542)  [82158](https://pubchem.ncbi.nlm.nih.gov/compound/82158)  [637564](https://pubchem.ncbi.nlm.nih.gov/compound/637564) |
|  |  | 2-Acetyl-5-methyl furan^13^  2-Acetylfuran^13^  2-Butylamine^13^  2-Cyclopentenone^13^  2-Ethyladamantane^13^  2-Hexanoylfuran^13^  2-Hexenal^13^  2-Hydroxypyridine^13^  2-Isopropylidene-3-methylhexa-3,5-dienal^13^  2-Methoxy-3-propenyl-phenol^13^  2-Methyl-2-cyclopentenone^13^  2-Methylcyclopentanone^13^  2-Nonyne^13^  3,5-Diethyl-toluene^13^  3-Octanol^13^  4,4-Dimethyl-2-cyclopenten-1-one^13^  4-Tert-pentylphenol^13^  Acetophenone^13^  Acetyl eugenol^13^  a-Cubebene^13^  Alloaromadendrene^13^  All-trans-squalene^13^  Anisole^13^  Apiol^13^  Asarone^13^  a-Terpinyl acetate^13^  Benzene acetaldehyde^13^  Bornyl acetate^13^  Cadina-3,9-diene^13^ | [14514](https://pubchem.ncbi.nlm.nih.gov/compound/14514)  [14505](https://pubchem.ncbi.nlm.nih.gov/compound/14505)  [24874](https://pubchem.ncbi.nlm.nih.gov/compound/24874)  [13588](https://pubchem.ncbi.nlm.nih.gov/compound/13588)  [139758](https://pubchem.ncbi.nlm.nih.gov/compound/139758)  [61738](https://pubchem.ncbi.nlm.nih.gov/compound/61738)  [5281168](https://pubchem.ncbi.nlm.nih.gov/compound/5281168)  [8871](https://pubchem.ncbi.nlm.nih.gov/compound/8871)  [5368460](https://pubchem.ncbi.nlm.nih.gov/compound/5368460)  [74069607](https://pubchem.ncbi.nlm.nih.gov/compound/74069607)  [14266](https://pubchem.ncbi.nlm.nih.gov/compound/14266)  [14265](https://pubchem.ncbi.nlm.nih.gov/compound/14265)  [140536](https://pubchem.ncbi.nlm.nih.gov/compound/140536)  [16302](https://pubchem.ncbi.nlm.nih.gov/compound/16302)  [11527](https://pubchem.ncbi.nlm.nih.gov/compound/11527)  [140955](https://pubchem.ncbi.nlm.nih.gov/compound/140955)  [6643](https://pubchem.ncbi.nlm.nih.gov/compound/6643)  [7410](https://pubchem.ncbi.nlm.nih.gov/compound/7410)  [7136](https://pubchem.ncbi.nlm.nih.gov/compound/7136)  [86609](https://pubchem.ncbi.nlm.nih.gov/compound/86609)  [91354](https://pubchem.ncbi.nlm.nih.gov/compound/91354)  [638072](https://pubchem.ncbi.nlm.nih.gov/compound/638072)  [7519](https://pubchem.ncbi.nlm.nih.gov/compound/7519)  [10659](https://pubchem.ncbi.nlm.nih.gov/compound/10659)  [636822](https://pubchem.ncbi.nlm.nih.gov/compound/636822)  [111037](https://pubchem.ncbi.nlm.nih.gov/compound/111037)  [998](https://pubchem.ncbi.nlm.nih.gov/compound/998)  [6448](https://pubchem.ncbi.nlm.nih.gov/compound/6448)  [10657](https://pubchem.ncbi.nlm.nih.gov/compound/10657) |
|  |  | Calarene^13^  Camphane^13^  Carvone^13^  Caryophyllene^13^  Caryophyllene oxide^13^  cis-Asarone^13^  cis-Geraniol^13^  cis-Lanceol^13^  cis-Nerolidol^13^  cis-Ocimene^13^  cis-Verbenol^13^  Cosmene^13^  Cuminaldehyde^13^  Curlone^13^  Cycloheptane^13^  Cyclohexanone^13^  Decane^13^  Dihydrocarveol^13^  Dihydrocarveol acetate^13^  Dodecane^13^ | [28481](https://pubchem.ncbi.nlm.nih.gov/compound/28481)  [92108](https://pubchem.ncbi.nlm.nih.gov/compound/92108)  [7439](https://pubchem.ncbi.nlm.nih.gov/compound/7439)  [5281515](https://pubchem.ncbi.nlm.nih.gov/compound/5281515)  [1742210](https://pubchem.ncbi.nlm.nih.gov/compound/1742210)  [5281758](https://pubchem.ncbi.nlm.nih.gov/compound/5281758)  [643820](https://pubchem.ncbi.nlm.nih.gov/compound/643820)  [6536796](https://pubchem.ncbi.nlm.nih.gov/compound/6536796)  [5320128](https://pubchem.ncbi.nlm.nih.gov/compound/5320128)  [5320250](https://pubchem.ncbi.nlm.nih.gov/compound/5320250)  [164888](https://pubchem.ncbi.nlm.nih.gov/compound/164888)  [5368451](https://pubchem.ncbi.nlm.nih.gov/compound/5368451)  [326](https://pubchem.ncbi.nlm.nih.gov/compound/326)  [196216](https://pubchem.ncbi.nlm.nih.gov/compound/196216)  [9265](https://pubchem.ncbi.nlm.nih.gov/compound/9265)  [7967](https://pubchem.ncbi.nlm.nih.gov/compound/7967)  [15600](https://pubchem.ncbi.nlm.nih.gov/compound/15600)  [12072](https://pubchem.ncbi.nlm.nih.gov/compound/12072)  [30248](https://pubchem.ncbi.nlm.nih.gov/compound/30248)  [8182](https://pubchem.ncbi.nlm.nih.gov/compound/8182) |
|  |  | Egomaketone^13^  Elemicin^13^  Elixene^13^  Elsholtziaketone^13^  Eremophilene^13^  Eucalyptol^13^  Farnesol^13^  Furfuryl alcohol^13^  Geraniol^13^  Germacrene D^13^  Germacrene D-4-ol^13^  Heneicosane^13^  Hexadecane^13^  Hexahydrofarnesyl acetone^13^  Humulene epoxide II^13^  Isobornyl acetate^13^  Isocaryophyllene^13^  Isoegomaketone^13^  Isoelemicin^13^  Isoeugenol^13^  Isolimonene^13^  Isomenthone^13^  Isopulegone^13^  Limonene oxide^13^  Limonene oxide, trans^13^  Linalool oxide trans^13^  Linalyl oxide cis^13^  Longifolene^13^  Longipinocarvone^13^ | [42978](https://pubchem.ncbi.nlm.nih.gov/compound/42978)  [10248](https://pubchem.ncbi.nlm.nih.gov/compound/10248)  [94254](https://pubchem.ncbi.nlm.nih.gov/compound/94254)  [521240](https://pubchem.ncbi.nlm.nih.gov/compound/521240)  [12309744](https://pubchem.ncbi.nlm.nih.gov/compound/12309744)  [2758](https://pubchem.ncbi.nlm.nih.gov/compound/2758)  [445070](https://pubchem.ncbi.nlm.nih.gov/compound/445070)  [7361](https://pubchem.ncbi.nlm.nih.gov/compound/7361)  [637566](https://pubchem.ncbi.nlm.nih.gov/compound/637566)  [5317570](https://pubchem.ncbi.nlm.nih.gov/compound/5317570)  [5352847](https://pubchem.ncbi.nlm.nih.gov/compound/5352847)  [12403](https://pubchem.ncbi.nlm.nih.gov/compound/12403)  [11006](https://pubchem.ncbi.nlm.nih.gov/compound/11006)  [10408](https://pubchem.ncbi.nlm.nih.gov/compound/10408)  [10704181](https://pubchem.ncbi.nlm.nih.gov/compound/10704181)  [247573](https://pubchem.ncbi.nlm.nih.gov/compound/247573)  [5281522](https://pubchem.ncbi.nlm.nih.gov/compound/5281522)  [5318556](https://pubchem.ncbi.nlm.nih.gov/compound/5318556)  [5318557](https://pubchem.ncbi.nlm.nih.gov/compound/5318557)  [853433](https://pubchem.ncbi.nlm.nih.gov/compound/853433)  [521268](https://pubchem.ncbi.nlm.nih.gov/compound/521268)  [6986](https://pubchem.ncbi.nlm.nih.gov/compound/6986)  [34645](https://pubchem.ncbi.nlm.nih.gov/compound/34645)  [91496](https://pubchem.ncbi.nlm.nih.gov/compound/91496)  [8029780](https://pubchem.ncbi.nlm.nih.gov/compound/8029780)  [6432254](https://pubchem.ncbi.nlm.nih.gov/compound/6432254)  [6428573](https://pubchem.ncbi.nlm.nih.gov/compound/6428573)  [289151](https://pubchem.ncbi.nlm.nih.gov/compound/289151)  [535296](https://pubchem.ncbi.nlm.nih.gov/compound/535296) |
|  |  | Massoia lactone^13^  Menthol^13^  Menthone^13^  Methyl chavicol^13^  Methyl eugenol^13^  Methyl geranate^13^  Methyl isoeugenol^13^  Methyl thymyl ether^13^  M-Mentha-6,8-diene^13^  Naginata ketone^13^  Nerol acetate^13^  n-Heptadecane^13^  Nonacosane^13^  Nonane^13^  n-Tricosane^13^  Octacosane^13^  Patchoulane^13^  p-Cymene^13^  Pentacosane^13^  Perilla ketone^13^  Perillaldehyde^13^  Perillene^13^ | [39914](https://pubchem.ncbi.nlm.nih.gov/compound/39914)  [1254](https://pubchem.ncbi.nlm.nih.gov/compound/1254)  [26447](https://pubchem.ncbi.nlm.nih.gov/compound/26447)  [8815](https://pubchem.ncbi.nlm.nih.gov/compound/8815)  [7127](https://pubchem.ncbi.nlm.nih.gov/compound/7127)  [5365910](https://pubchem.ncbi.nlm.nih.gov/compound/5365910)  [7128](https://pubchem.ncbi.nlm.nih.gov/compound/7128)  [14104](https://pubchem.ncbi.nlm.nih.gov/compound/14104)  [102625](https://pubchem.ncbi.nlm.nih.gov/compound/102625)  [564412](https://pubchem.ncbi.nlm.nih.gov/compound/564412)  [1549025](https://pubchem.ncbi.nlm.nih.gov/compound/1549025)  [12398](https://pubchem.ncbi.nlm.nih.gov/compound/12398)  [12409](https://pubchem.ncbi.nlm.nih.gov/compound/12409)  [8141](https://pubchem.ncbi.nlm.nih.gov/compound/8141)  [12534](https://pubchem.ncbi.nlm.nih.gov/compound/12534)  [12408](https://pubchem.ncbi.nlm.nih.gov/compound/12408)  [29408](https://pubchem.ncbi.nlm.nih.gov/compound/29408)  [7463](https://pubchem.ncbi.nlm.nih.gov/compound/7463)  [12406](https://pubchem.ncbi.nlm.nih.gov/compound/12406)  [68381](https://pubchem.ncbi.nlm.nih.gov/compound/68381)  [16441](https://pubchem.ncbi.nlm.nih.gov/compound/16441)  [68316](https://pubchem.ncbi.nlm.nih.gov/compound/68316) |
|  |  | Perillic acid^13^  Perillyl alcohol^13^  Piperitenone^13^  p-Menth-1-en-4-ol^13^  p-Menth-1-en-8-ol^13^  p-Mentha-3,8-diene^13^  Pulegone^13^  Phytol^13^  Sabinene^13^  Santolina triene^13^  Spathulenol^13^  Terpinolene^13^  Thujyl alcohol^13^  trans-Nerolidol^13^  trans-Shisool^13^  Triacontane^13^  Tridecane^13^  Valencene^13^  Valeric acid, pent-2-en-4-ynyl ester^13^  Viridiflorene^13^  Viridiflorol^13^  α-Bulnesene^13^  α-Cadinol^13^  α-Caryophyllene^13^  α-Citral^13^ | [1256](https://pubchem.ncbi.nlm.nih.gov/compound/1256)  [10819](https://pubchem.ncbi.nlm.nih.gov/compound/10819)  [381152](https://pubchem.ncbi.nlm.nih.gov/compound/381152)  [11230](https://pubchem.ncbi.nlm.nih.gov/compound/11230)  [17100](https://pubchem.ncbi.nlm.nih.gov/compound/17100)  [521851](https://pubchem.ncbi.nlm.nih.gov/compound/521851)  [442495](https://pubchem.ncbi.nlm.nih.gov/compound/442495)  [5280435](https://pubchem.ncbi.nlm.nih.gov/compound/5280435)  [18818](https://pubchem.ncbi.nlm.nih.gov/compound/18818)  [519872](https://pubchem.ncbi.nlm.nih.gov/compound/519872)  [92231](https://pubchem.ncbi.nlm.nih.gov/compound/92231)  [11463](https://pubchem.ncbi.nlm.nih.gov/compound/11463)  [10550](https://pubchem.ncbi.nlm.nih.gov/compound/10550)  [5284507](https://pubchem.ncbi.nlm.nih.gov/compound/5284507)  [519954](https://pubchem.ncbi.nlm.nih.gov/compound/519954)  [12535](https://pubchem.ncbi.nlm.nih.gov/compound/12535)  [12388](https://pubchem.ncbi.nlm.nih.gov/compound/12388)  [9855795](https://pubchem.ncbi.nlm.nih.gov/compound/9855795)  [5353036](https://pubchem.ncbi.nlm.nih.gov/compound/5353036)  [10910653](https://pubchem.ncbi.nlm.nih.gov/compound/10910653)  [11996452](https://pubchem.ncbi.nlm.nih.gov/compound/11996452)  [520826](https://pubchem.ncbi.nlm.nih.gov/compound/520826)  [10398656](https://pubchem.ncbi.nlm.nih.gov/compound/10398656)  [5281520](https://pubchem.ncbi.nlm.nih.gov/compound/5281520)  [638011](https://pubchem.ncbi.nlm.nih.gov/compound/638011) |
|  |  | α-Copaene^13^  α-Fenchene^13^  α-Patchoulene^13^  α-Santalol^13^  β-Cubebene^13^  β-Bourbonene^13^  β-Cyclocitral^13^  β-Elemene^13^  β-Farnesene^13^  β-Guaiene^13^  β-Gurjunene^13^  β-Ionone^13^  β-Pinene^13^  β-Selinene^13^  β-Phellandrene^13^  β-Terpinene^13^  γ-Pyronene^13^  δ-Cadinene^13^  δ-Elemene^13^ | [70678558](https://pubchem.ncbi.nlm.nih.gov/compound/70678558)  [12309839](https://pubchem.ncbi.nlm.nih.gov/compound/12309839)  [521710](https://pubchem.ncbi.nlm.nih.gov/compound/521710)  [5281531](https://pubchem.ncbi.nlm.nih.gov/compound/5281531)  [93081](https://pubchem.ncbi.nlm.nih.gov/compound/93081)  [324224](https://pubchem.ncbi.nlm.nih.gov/compound/324224)  [9895](https://pubchem.ncbi.nlm.nih.gov/compound/9895)  [6918391](https://pubchem.ncbi.nlm.nih.gov/compound/6918391)  [5281517](https://pubchem.ncbi.nlm.nih.gov/compound/5281517)  [6949](https://pubchem.ncbi.nlm.nih.gov/compound/6949)  [6450812](https://pubchem.ncbi.nlm.nih.gov/compound/6450812)  [638014](https://pubchem.ncbi.nlm.nih.gov/compound/638014)  [14896](https://pubchem.ncbi.nlm.nih.gov/compound/14896)  [442393](https://pubchem.ncbi.nlm.nih.gov/compound/442393)  [11142](https://pubchem.ncbi.nlm.nih.gov/compound/11142)  [66841](https://pubchem.ncbi.nlm.nih.gov/compound/66841)  [578237](https://pubchem.ncbi.nlm.nih.gov/compound/578237)  [441005](https://pubchem.ncbi.nlm.nih.gov/compound/441005)  [12309449](https://pubchem.ncbi.nlm.nih.gov/compound/12309449) |
| 27. | *Aralia cordata*  (9) | Methyl-α-D-fructofuranoside^14^  Methyl-β-D-fructofuranoside^14^  3,5-Di-O-caffeoylquinic acid^14^  Pimaric acid^14^  (-)-Kaur-16-en-19-oic acid^14^  Falcarindiol^14^  17-Hydroxy-ent-kaur-15-en-19-oic acid^14^  Alpha-mono palmitin^14^  Continentalic acid^15^ | [6325664](https://pubchem.ncbi.nlm.nih.gov/compound/6325664)  [128889](https://pubchem.ncbi.nlm.nih.gov/compound/128889)  [6474310](https://pubchem.ncbi.nlm.nih.gov/compound/6474310)  [220338](https://pubchem.ncbi.nlm.nih.gov/compound/220338)  [73062](https://pubchem.ncbi.nlm.nih.gov/compound/73062)  [5281148](https://pubchem.ncbi.nlm.nih.gov/compound/5281148)  [169654](https://pubchem.ncbi.nlm.nih.gov/compound/169654)  [14900](https://pubchem.ncbi.nlm.nih.gov/compound/14900)  [10086296](https://pubchem.ncbi.nlm.nih.gov/compound/10086296) |
| 28. | *Anemarrhena asphodeloides*  (39) | Neomangiferin^16^  Mangiferin^16^  Isomangiferin^16^  Vitexin^16^  Isosakuranetin^16^  Macrostemonoside J^16^  Asparagoside G^16^  Tomatoside A^16^  Timosaponin BII^16^  Officinalisinin-I^16^  Timosaponin D^16^  Filicinoside-A^16^  Timosaponin F^16^  Anemarrhenasaponin I^16^  Anemarrhenasaponin II^16^  Anemarrhenasaponin III^16^  F-gitonin^16^  Timosaponin AIV^16^  Timosaponin AIII^16^  Desgalactotigonin^16^  Timosaponin AI^16^  cis-hinokiresinol^16^  monomethyl-cis-hinokiresinol^16^  Foliamangiferoside A^16^  2,6,4′-trihydroxy-4- methoxybenzophenone^16^  Phytosphingosine^16^  Dimethisterone^16^ | [6918448](https://pubchem.ncbi.nlm.nih.gov/compound/6918448)  [5281647](https://pubchem.ncbi.nlm.nih.gov/compound/5281647)  [5318597](https://pubchem.ncbi.nlm.nih.gov/compound/5318597)  [5280441](https://pubchem.ncbi.nlm.nih.gov/compound/5280441)  [160481](https://pubchem.ncbi.nlm.nih.gov/compound/160481)  [101669618](https://pubchem.ncbi.nlm.nih.gov/compound/101669618)  [3042722](https://pubchem.ncbi.nlm.nih.gov/compound/3042722)  [426059](https://pubchem.ncbi.nlm.nih.gov/compound/426059)  [44575945](https://pubchem.ncbi.nlm.nih.gov/compound/44575945)  [441889](https://pubchem.ncbi.nlm.nih.gov/compound/441889)  [132545772](https://pubchem.ncbi.nlm.nih.gov/compound/132545772)  [190854](https://pubchem.ncbi.nlm.nih.gov/compound/190854)  [101005526](https://pubchem.ncbi.nlm.nih.gov/compound/101005526)  [101672279](https://pubchem.ncbi.nlm.nih.gov/compound/101672279)  [101672380](https://pubchem.ncbi.nlm.nih.gov/compound/101672380)  [101672280](https://pubchem.ncbi.nlm.nih.gov/compound/101672280)  [44559009](https://pubchem.ncbi.nlm.nih.gov/compound/44559009)  [154572708](https://pubchem.ncbi.nlm.nih.gov/compound/154572708)  [15953793](https://pubchem.ncbi.nlm.nih.gov/compound/15953793)  [162401](https://pubchem.ncbi.nlm.nih.gov/compound/162401)  [71767755](https://pubchem.ncbi.nlm.nih.gov/compound/71767755)  [5281830](https://pubchem.ncbi.nlm.nih.gov/compound/5281830)  [5319736](https://pubchem.ncbi.nlm.nih.gov/compound/5319736)  [46206548](https://pubchem.ncbi.nlm.nih.gov/compound/46206548)  [10467773](https://pubchem.ncbi.nlm.nih.gov/compound/10467773)  [122121](https://pubchem.ncbi.nlm.nih.gov/compound/122121)  [6607](https://pubchem.ncbi.nlm.nih.gov/compound/6607) |
|  |  | Macrostemonoside F^17^  Platycodin D^17^  Platycoside A^17^  Platycodin D2^17^  Polygalacin D2^17^  Platycodin D3^17^  trans-Hinokiresinol^17^  Isosarsasapogenin^18^  Markogenin^18^  Neogitogenin^18^  Sarsasapogenin^18^  Diosgenin^18^ | [192523](https://pubchem.ncbi.nlm.nih.gov/compound/192523)  [162859](https://pubchem.ncbi.nlm.nih.gov/compound/162859)  [50900942](https://pubchem.ncbi.nlm.nih.gov/compound/50900942)  [53317652](https://pubchem.ncbi.nlm.nih.gov/compound/53317652)  [53325781](https://pubchem.ncbi.nlm.nih.gov/compound/53325781)  [70698293](https://pubchem.ncbi.nlm.nih.gov/compound/70698293)  [12310493](https://pubchem.ncbi.nlm.nih.gov/compound/12310493)  [91439](https://pubchem.ncbi.nlm.nih.gov/compound/91439)  [12304414](https://pubchem.ncbi.nlm.nih.gov/compound/12304414)  [12304409](https://pubchem.ncbi.nlm.nih.gov/compound/12304409)  [92095](https://pubchem.ncbi.nlm.nih.gov/compound/92095)  [99474](https://pubchem.ncbi.nlm.nih.gov/compound/99474) |
| 29. | *Arctium lappa*  (8) | Arctigenin^19^  Arctiin^19^  Trachelogenin^19^  Lappaol F^19^  Diarctigenin^19^  Methyl palmitate^20^  Methyl linoleate^20^  Methyl oleate^20^ | [64981](https://pubchem.ncbi.nlm.nih.gov/compound/64981)  [100528](https://pubchem.ncbi.nlm.nih.gov/compound/100528)  [452855](https://pubchem.ncbi.nlm.nih.gov/compound/452855)  [73425459](https://pubchem.ncbi.nlm.nih.gov/compound/73425459)  [16215736](https://pubchem.ncbi.nlm.nih.gov/compound/16215736)  [8181](https://pubchem.ncbi.nlm.nih.gov/compound/8181)  [5284421](https://pubchem.ncbi.nlm.nih.gov/compound/5284421)  [5364509](https://pubchem.ncbi.nlm.nih.gov/compound/5364509) |
| 30. | *Artemisia princeps*  (21) | Hexanal^21^  Benzeneacetaldehyde^21^  cis-Sabinene hydrate^21^  trans-Sabinene hydrate^21^  cis-Verbenol^21^  trans-Verbenol^21^  cis-Chrysanthenol^21^  Verbenone^21^  Chrysanthenyl acetate^21^  Lavandulyl acetate^21^  Silphiperfol-5-ene^21^  Germacrene D^21^  Zingiberene^21^  Germacrene D 4-ol^21^  Neophytadiene^21^  Hexadecanoic acid^21^  Phytol^21^  Nonacosane^21^  Hentriacontane^21^  Dotriacontane^21^  Tritriacontane^21^ | [6184](https://pubchem.ncbi.nlm.nih.gov/compound/6184)  [998](https://pubchem.ncbi.nlm.nih.gov/compound/998)  [101629835](https://pubchem.ncbi.nlm.nih.gov/compound/101629835)  [12315151](https://pubchem.ncbi.nlm.nih.gov/compound/12315151)  [164888](https://pubchem.ncbi.nlm.nih.gov/compound/164888)  [89664](https://pubchem.ncbi.nlm.nih.gov/compound/89664)  [527032](https://pubchem.ncbi.nlm.nih.gov/compound/527032)  [29025](https://pubchem.ncbi.nlm.nih.gov/compound/29025)  [162747](https://pubchem.ncbi.nlm.nih.gov/compound/162747)  [30247](https://pubchem.ncbi.nlm.nih.gov/compound/30247)  [91747334](https://pubchem.ncbi.nlm.nih.gov/compound/91747334)  [5317570](https://pubchem.ncbi.nlm.nih.gov/compound/5317570)  [92776](https://pubchem.ncbi.nlm.nih.gov/compound/92776)  [5352847](https://pubchem.ncbi.nlm.nih.gov/compound/5352847)  [10446](https://pubchem.ncbi.nlm.nih.gov/compound/10446)  [985](https://pubchem.ncbi.nlm.nih.gov/compound/985)  [5280435](https://pubchem.ncbi.nlm.nih.gov/compound/5280435)  [12409](https://pubchem.ncbi.nlm.nih.gov/compound/12409)  [12410](https://pubchem.ncbi.nlm.nih.gov/compound/12410)  [11008](https://pubchem.ncbi.nlm.nih.gov/compound/11008)  [12411](https://pubchem.ncbi.nlm.nih.gov/compound/12411) |

**References:**

1. Yoo, K. M., Hwang, I. K., Park, J. H. & Moon, B. K. Major phytochemical composition of 3 native korean citrus varieties and bioactive activity on V79-4 cells induced by oxidative stress. *J. Food Sci.* **74**, C462–C468 (2009).

2. Ma, Y. Q. *et al.* Phenolic compounds and antioxidant activity of extracts from ultrasonic treatment of satsuma mandarin (Citrus unshiu Marc.) peels. *J. Agric. Food Chem.* **56**, 5682–5690 (2008).

3. Eom, H. J. *et al.* Flavonoids and a Limonoid from the Fruits of Citrus unshiu and Their Biological Activity. *J. Agric. Food Chem.* **64**, 7171–7178 (2016).

4. Kim, D. S. & Lim, S. Bin. Semi-continuous subcritical water extraction of flavonoids from citrus unshiu peel: Their antioxidant and enzyme inhibitory activities. *Antioxidants* **9**, (2020).

5. Kim, M. J. *et al.* Chemical composition and anti-inflammation activity of essential oils from Citrus unshiu flower. *Nat. Prod. Commun.* **9**, 727–730 (2014).

6. Phatak, R. S. Phytochemistry, pharmacological activities and intellectual property landscape of gardenia Jasminoides Ellis: A review. *Pharmacogn. J.* **7**, 254–265 (2015).

7. Jing-ying, Z., Shi-sheng, W., Wei-jie, Z., Qi-ling, S. & Qing-wei, M. Chemical Study on Aerial Parts of Gentiana scabra. *Nat. Prod. Res. Dev.* **21**, 556–585 (2009).

8. He, Y. M. *et al.* The anti-inflammatory secoiridoid glycosides from Gentianae Scabrae Radix: The root and rhizome of Gentiana scabra. *J. Nat. Med.* **69**, 303–312 (2015).

9. Li, W. *et al.* Triterpenoids isolated from the rhizomes and roots of Gentiana scabra and their inhibition of indoleamine 2,3-dioxygenase. *Arch. Pharm. Res.* **38**, 2124–2130 (2015).

10. Luo, H. *et al.* A review of the phytochemistry and pharmacological activities of Magnoliae officinalis cortex. *J. Ethnopharmacol.* **236**, 412–442 (2019).

11. Chen, M. H., Chen, X. J., Wang, M., Lin, L. G. & Wang, Y. T. Ophiopogon japonicus - A phytochemical, ethnomedicinal and pharmacological review. *J. Ethnopharmacol.* **181**, 193–213 (2016).

12. Ling, Y. *et al.* Rapid screening and identification of chemical constituents from ophiopogon japonicus by high-performance liquid chromatography coupled to electrospray ionization and quadrupole time-of-flight mass spectrometry. *J. Chromatogr. Sci.* **58**, 641–650 (2020).

13. Ahmed, H. M. Ethnomedicinal, phytochemical and pharmacological investigations of Perilla frutescens (L.) Britt. *Molecules* **24**, (2019).

14. Hyun, S. K., Jung, H. A., Min, B. S., Jung, J. H. & Choi, J. S. Isolation of phenolics, nucleosides, saccharides and an alkaloid from the root of Aralia cordata. *Nat. Prod. Sci.* **16**, 20–25 (2010).

15. Lee, M. K. *et al.* Quantitative determination of diterpenoids from the roots of Aralia cordata. *Nat. Prod. Sci.* **15**, 50–54 (2009).

16. Ji, D., Huang, Z. yan, Fei, C. hao, Xue, W. wei & Lu, T. lin. Comprehensive profiling and characterization of chemical constituents of rhizome of Anemarrhena asphodeloides Bge. *J. Chromatogr. B Anal. Technol. Biomed. Life Sci.* **1060**, 355–366 (2017).

17. Wang, Z. *et al.* Anti-inflammatory activities of compounds isolated from the rhizome of anemarrhena asphodeloides. *Molecules* **23**, 1–15 (2018).

18. Wang, Y. *et al.* The genus Anemarrhena Bunge: A review on ethnopharmacology, phytochemistry and pharmacology. *J. Ethnopharmacol.* **153**, 42–60 (2014).

19. Chan, Y. S. *et al.* A review of the pharmacological effects of Arctium lappa (burdock). *Inflammopharmacology* **19**, 245–254 (2011).

20. Arctium, L., Iyazawa, M. M., Agi, N. Y. & Aguchi, K. T. Inhibitory Compounds of a-Glucosidase Activity. *Jounal Oleo Sci.* **54**, 589–594 (2005).

21. Nishidono, Y., Chiyomatsu, T., Sanuki, K., Tezuka, Y. & Tanaka, K. Analysis of Seasonal Variations of the Volatile Constituents in Artemisia princeps (Japanese Mugwort) Leaves by Metabolomic Approach. *Nat. Prod. Commun.* **14**, (2019).
