# Supplementary material for: An in silico approach to develop potential therapies against Middle East Respiratory Syndrome Coronavirus (MERS-CoV)
Source: Heliyon. 2024 Feb 9;10(4):e25837. doi: 10.1016/j.heliyon.2024.e25837 (PMC10877303; doi:10.1016/j.heliyon.2024.e25837)
Supplement: Multimedia component 4 [file mmc4.docx]

| **SL. No.** | **Plant Name** | **Compounds** | **CID** |
| --- | --- | --- | --- |
| 31. | *Pinellia ternate*  (74) | Isolariciresinol^1^  isolariciresinol 9-O-beta-D-glucopyranoside^1^  pinoresinol-4-O-beta-D glucoside^1^  dehydrodiconiferyl alcohol 4-O-b-D-glucopyranoside^1^  neo-olivil^1^  medioresinol^1^  americanol A^1^  pinoresinol^1^  burselignan^1^  L-Ephedrine^2^  Inosine^2^  Cytidine^2^  Pedatisectine B^2^  8-Octadecenoic acid^2^  Pentadecanoic acid^2^  9-Hexadecenoic acid^2^  Hexadecanoic acid^2^  Heptadecanoic acid^2^  7-Hexadecenoic acid^2^  Octadecanoic acid^2^  9-Oxo-nonanoic acid^2^ | [160521](https://pubchem.ncbi.nlm.nih.gov/compound/160521)  [85210374](https://pubchem.ncbi.nlm.nih.gov/compound/85210374)  [486614](https://pubchem.ncbi.nlm.nih.gov/compound/486614)  [5316442](https://pubchem.ncbi.nlm.nih.gov/compound/5316442)  [9976812](https://pubchem.ncbi.nlm.nih.gov/compound/9976812)  [181681](https://pubchem.ncbi.nlm.nih.gov/compound/181681)  [637304](https://pubchem.ncbi.nlm.nih.gov/compound/637304)  [73399](https://pubchem.ncbi.nlm.nih.gov/compound/73399)  [11631864](https://pubchem.ncbi.nlm.nih.gov/compound/11631864)  [9294](https://pubchem.ncbi.nlm.nih.gov/compound/9294)  [135398641](https://pubchem.ncbi.nlm.nih.gov/compound/135398641)  [6175](https://pubchem.ncbi.nlm.nih.gov/compound/6175)  [190](https://pubchem.ncbi.nlm.nih.gov/compound/190)  [5282758](https://pubchem.ncbi.nlm.nih.gov/compound/5282758)  [13849](https://pubchem.ncbi.nlm.nih.gov/compound/13849)  [5282745](https://pubchem.ncbi.nlm.nih.gov/compound/5282745)  [985](https://pubchem.ncbi.nlm.nih.gov/compound/985)  [10465](https://pubchem.ncbi.nlm.nih.gov/compound/10465)  [543268](https://pubchem.ncbi.nlm.nih.gov/compound/543268)  [5281](https://pubchem.ncbi.nlm.nih.gov/compound/5281)  [75704](https://pubchem.ncbi.nlm.nih.gov/compound/75704) |
|  |  | 11-Eicosenoic acid^2^  Eicosanoic acid^2^  10,13-Eicosadienoic acid^2^  Docosanoic acid^2^  Pinellic acid^2^  Succinic acid^2^  3-Methyleicosane^2^  3-Decyne^2^  2-Methyldecane^2^  Octadecane^2^  2,6,10-Trimethyltetradecane^2^  2,5-Dimethyltetradecane^2^  Vinylcyclohexane^2^  1-Octene^2^  6-Methyl-2-heptanone^2^  3-Nonanone^2^  Cis-4-decenal^2^  2-Undecanone^2^  9-Heptadecanol^2^  Anethole^2^  Citronellal^2^  Aromandendrene^2^  Farnesane^2^  β-Patchoulene^2^  α-Elemol^2^  1-Methyl-4-(1-methylethenyl)-cyclohexene^2^  Dibutyl phthalate^2^  2,6-Di-tert-butyl-4-methylphenol^2^  2-Pentylfuran^2^  Furfural^2^ | [5282768](https://pubchem.ncbi.nlm.nih.gov/compound/5282768)  [10467](https://pubchem.ncbi.nlm.nih.gov/compound/10467)  [72444876](https://pubchem.ncbi.nlm.nih.gov/compound/72444876)  [8215](https://pubchem.ncbi.nlm.nih.gov/compound/8215)  [9858729](https://pubchem.ncbi.nlm.nih.gov/compound/9858729)  [1110](https://pubchem.ncbi.nlm.nih.gov/compound/1110)  [98417](https://pubchem.ncbi.nlm.nih.gov/compound/98417)  [75425](https://pubchem.ncbi.nlm.nih.gov/compound/75425)  [23415](https://pubchem.ncbi.nlm.nih.gov/compound/23415)  [11635](https://pubchem.ncbi.nlm.nih.gov/compound/11635)  [85785](https://pubchem.ncbi.nlm.nih.gov/compound/85785)  [41836](https://pubchem.ncbi.nlm.nih.gov/compound/41836)  [12757](https://pubchem.ncbi.nlm.nih.gov/compound/12757)  [8125](https://pubchem.ncbi.nlm.nih.gov/compound/8125)  [13572](https://pubchem.ncbi.nlm.nih.gov/compound/13572)  [61235](https://pubchem.ncbi.nlm.nih.gov/compound/61235)  [5362620](https://pubchem.ncbi.nlm.nih.gov/compound/5362620)  [8163](https://pubchem.ncbi.nlm.nih.gov/compound/8163)  [136435](https://pubchem.ncbi.nlm.nih.gov/compound/136435)  [637563](https://pubchem.ncbi.nlm.nih.gov/compound/637563)  [7794](https://pubchem.ncbi.nlm.nih.gov/compound/7794)  [91354](https://pubchem.ncbi.nlm.nih.gov/compound/91354)  [19773](https://pubchem.ncbi.nlm.nih.gov/compound/19773)  [101731](https://pubchem.ncbi.nlm.nih.gov/compound/101731)  [92138](https://pubchem.ncbi.nlm.nih.gov/compound/92138)  [6453304](https://pubchem.ncbi.nlm.nih.gov/compound/6453304)  [3026](https://pubchem.ncbi.nlm.nih.gov/compound/3026)  [31404](https://pubchem.ncbi.nlm.nih.gov/compound/31404)  [19602](https://pubchem.ncbi.nlm.nih.gov/compound/19602)  [7362](https://pubchem.ncbi.nlm.nih.gov/compound/7362) |
|  |  | 2,4-Dimethyl furan^2^  Anisic acid^2^  Pulegone^2^  Isopulegol^2^  3-Nonyne^2^  2-Methylnonane^2^  3,4-Dihydroxycinnamyl alcohol^2^  Sachaliside 1^2^  Stigmast-4-en-3-one^2^  Cycloartenol^2^  Protocatechuic aldehyde^2^  Shogaol^2^  Gingerol^2^  Erythritol^2^  Melissane^2^  Nonacosane^2^  Homogentisic acid^2^  Benzene-1,4-diol^2^  Benzene-1,2-diol^2^  Monogalactosyldiacylglycerol^2^  Soyacerebroside I^2^  Soyacerebroside II^2^  N-acetylglutamate^2^ | [19462](https://pubchem.ncbi.nlm.nih.gov/compound/19462)  [7478](https://pubchem.ncbi.nlm.nih.gov/compound/7478)  [442495](https://pubchem.ncbi.nlm.nih.gov/compound/442495)  [170833](https://pubchem.ncbi.nlm.nih.gov/compound/170833)  [140649](https://pubchem.ncbi.nlm.nih.gov/compound/140649)  [13379](https://pubchem.ncbi.nlm.nih.gov/compound/13379)  [5282096](https://pubchem.ncbi.nlm.nih.gov/compound/5282096)  [14048613](https://pubchem.ncbi.nlm.nih.gov/compound/14048613)  [5484202](https://pubchem.ncbi.nlm.nih.gov/compound/5484202)  [92110](https://pubchem.ncbi.nlm.nih.gov/compound/92110)  [8768](https://pubchem.ncbi.nlm.nih.gov/compound/8768)  [5281794](https://pubchem.ncbi.nlm.nih.gov/compound/5281794)  [442793](https://pubchem.ncbi.nlm.nih.gov/compound/442793)  [222285](https://pubchem.ncbi.nlm.nih.gov/compound/222285)  [182192](https://pubchem.ncbi.nlm.nih.gov/compound/182192)  [12409](https://pubchem.ncbi.nlm.nih.gov/compound/12409)  [780](https://pubchem.ncbi.nlm.nih.gov/compound/780)  [785](https://pubchem.ncbi.nlm.nih.gov/compound/785)  [289](https://pubchem.ncbi.nlm.nih.gov/compound/289)  [90657729](https://pubchem.ncbi.nlm.nih.gov/compound/90657729)  [11104507](https://pubchem.ncbi.nlm.nih.gov/compound/11104507)  [15599558](https://pubchem.ncbi.nlm.nih.gov/compound/15599558)  [70914](https://pubchem.ncbi.nlm.nih.gov/compound/70914) |
| 32. | *Platycodon grandifloras*  (68) | Platycoside A^3^  Platycoside B^3^  Platycoside C^3^  Platycoside D^3^  Platycoside E^3^  Platycoside G1^3^  Platycoside F^3^  Platycoside G2^3^  Platycoside G3^3^  Platycoside H^3^  Platycoside I^3^  Platycoside J^3^  Platycoside K^3^  Platycoside L^3^  Platycoside M-1^3^  Platycoside M-2^3^  Platycoside M-3^3^  Platycodin A^3^  Platycodin C^3^  Platycodin D^3^  Deapioplatycodin D^3^  Platycodin D2^3^  Platycodin J^3^  Platycodin K^3^  Platycodin L^3^ | [50900942](https://pubchem.ncbi.nlm.nih.gov/compound/50900942)  [101937508](https://pubchem.ncbi.nlm.nih.gov/compound/101937508)  [101937509](https://pubchem.ncbi.nlm.nih.gov/compound/101937509)  [100974704](https://pubchem.ncbi.nlm.nih.gov/compound/100974704)  [70698202](https://pubchem.ncbi.nlm.nih.gov/compound/70698202)  [70698289](https://pubchem.ncbi.nlm.nih.gov/compound/70698289)  [101048500](https://pubchem.ncbi.nlm.nih.gov/compound/101048500)  [102217526](https://pubchem.ncbi.nlm.nih.gov/compound/102217526)  [70698263](https://pubchem.ncbi.nlm.nih.gov/compound/70698263)  [11665339](https://pubchem.ncbi.nlm.nih.gov/compound/11665339)  [11622299](https://pubchem.ncbi.nlm.nih.gov/compound/11622299)  [11528185](https://pubchem.ncbi.nlm.nih.gov/compound/11528185)  [102004765](https://pubchem.ncbi.nlm.nih.gov/compound/102004765)  [11556931](https://pubchem.ncbi.nlm.nih.gov/compound/11556931)  [101403595](https://pubchem.ncbi.nlm.nih.gov/compound/101403595)  [101403596](https://pubchem.ncbi.nlm.nih.gov/compound/101403596)  [101403597](https://pubchem.ncbi.nlm.nih.gov/compound/101403597)  [46173910](https://pubchem.ncbi.nlm.nih.gov/compound/46173910)  [46173919](https://pubchem.ncbi.nlm.nih.gov/compound/46173919)  [162859](https://pubchem.ncbi.nlm.nih.gov/compound/162859)  [70698266](https://pubchem.ncbi.nlm.nih.gov/compound/70698266)  [53317652](https://pubchem.ncbi.nlm.nih.gov/compound/53317652)  [102052427](https://pubchem.ncbi.nlm.nih.gov/compound/102052427)  [102052428](https://pubchem.ncbi.nlm.nih.gov/compound/102052428)  [102052429](https://pubchem.ncbi.nlm.nih.gov/compound/102052429) |
|  |  | 3-O-β-D-glucopyranosylplatycodigenin^3^  Polygalacin D^3^  20-O-acetylPolygalacin D^3^  30-O-acetylPolygalacin D^3^  Polygalacin D2^3^  30-O-acetylPolygalacin D2^3^  Platyconic acid A^3^  Platyconic acid B lactone^3^  Deapioplatyconic acid B lactone^3^  Platycogenic acid A^3^  Platyconic acid B^3^  Platyconic acid C^3^  Platyconic acid D^3^  Platyconic acid E^3^  Platycogenic acid B^3^  Platycogenic acid C^3^  Platyconin^3^ | [102185205](https://pubchem.ncbi.nlm.nih.gov/compound/102185205)  [46173909](https://pubchem.ncbi.nlm.nih.gov/compound/46173909)  [53321792](https://pubchem.ncbi.nlm.nih.gov/compound/53321792)  [53321793](https://pubchem.ncbi.nlm.nih.gov/compound/53321793)  [53325781](https://pubchem.ncbi.nlm.nih.gov/compound/53325781)  [53321794](https://pubchem.ncbi.nlm.nih.gov/compound/53321794)  [70698300](https://pubchem.ncbi.nlm.nih.gov/compound/70698300)  [50900852](https://pubchem.ncbi.nlm.nih.gov/compound/50900852)  [50900941](https://pubchem.ncbi.nlm.nih.gov/compound/50900941)  [20056221](https://pubchem.ncbi.nlm.nih.gov/compound/20056221)  [101495504](https://pubchem.ncbi.nlm.nih.gov/compound/101495504)  [102052424](https://pubchem.ncbi.nlm.nih.gov/compound/102052424)  [102052425](https://pubchem.ncbi.nlm.nih.gov/compound/102052425)  [102052426](https://pubchem.ncbi.nlm.nih.gov/compound/102052426)  [69570716](https://pubchem.ncbi.nlm.nih.gov/compound/69570716)  [69569686](https://pubchem.ncbi.nlm.nih.gov/compound/69569686)  [90659256](https://pubchem.ncbi.nlm.nih.gov/compound/90659256) |
|  |  | (2R, 3R) taxifolin^3^  quercetin-7-O-glucoside^3^  quercetin-7-O-rutinoside^3^  luteolin-7-O-glucoside^3^  apigenin-7-O-glucoside^3^  p-hydroxybenzoic acid^3^  2,3-dihydroxybenzoic acid^3^  2-hydroxy4-methoxybenzoic acid^3^  homovanillic acid^3^  spinasterol^3^  δ-7-stigmastenone-3^3^  Apigenin^3^  Luteolin^3^  Flavoplatycoside^3^  3,4-dimethoxycinnamic acid^3^  Isoferulic acid^3^  m-coumaric acid^3^  p-coumaric acid^4^  α-resorcylic acid^4^  Chlorogenic acid^4^  Lobetyolin^4^  Lobetyolinin^4^  Iobetyol^4^  Betulin^4^  β-sitosterol^4^  Grandoside^4^ | [439533](https://pubchem.ncbi.nlm.nih.gov/compound/439533)  [5282160](https://pubchem.ncbi.nlm.nih.gov/compound/5282160)  [101764560](https://pubchem.ncbi.nlm.nih.gov/compound/101764560)  [5280637](https://pubchem.ncbi.nlm.nih.gov/compound/5280637)  [12304093](https://pubchem.ncbi.nlm.nih.gov/compound/12304093)  [135](https://pubchem.ncbi.nlm.nih.gov/compound/135)  [19](https://pubchem.ncbi.nlm.nih.gov/compound/19)  [75231](https://pubchem.ncbi.nlm.nih.gov/compound/75231)  [1738](https://pubchem.ncbi.nlm.nih.gov/compound/1738)  [5281331](https://pubchem.ncbi.nlm.nih.gov/compound/5281331)  [5748344](https://pubchem.ncbi.nlm.nih.gov/compound/5748344)  [5280443](https://pubchem.ncbi.nlm.nih.gov/compound/5280443)  [5280445](https://pubchem.ncbi.nlm.nih.gov/compound/5280445)  [10416329](https://pubchem.ncbi.nlm.nih.gov/compound/10416329)  [717531](https://pubchem.ncbi.nlm.nih.gov/compound/717531)  [736186](https://pubchem.ncbi.nlm.nih.gov/compound/736186)  [637541](https://pubchem.ncbi.nlm.nih.gov/compound/637541)  [637542](https://pubchem.ncbi.nlm.nih.gov/compound/637542)  [7424](https://pubchem.ncbi.nlm.nih.gov/compound/7424)  [1794427](https://pubchem.ncbi.nlm.nih.gov/compound/1794427)  [53486204](https://pubchem.ncbi.nlm.nih.gov/compound/53486204)  [5459227](https://pubchem.ncbi.nlm.nih.gov/compound/5459227)  [5807986](https://pubchem.ncbi.nlm.nih.gov/compound/5807986)  [72326](https://pubchem.ncbi.nlm.nih.gov/compound/72326)  [521199](https://pubchem.ncbi.nlm.nih.gov/compound/521199)  [10341593](https://pubchem.ncbi.nlm.nih.gov/compound/10341593) |
| 33. | *Rehmannia glutinosa*  (45) | Lariciresinol^5^  hierochin D^5^  yemuoside 1^5^  darendoside B^5^  decaffeoyl acteoside^5^  jionoside B_1_^5^  [catalpol](https://www.sciencedirect.com/topics/pharmacology-toxicology-and-pharmaceutical-science/catalpol)^5^  ajugol^5^  6-O-vanilloylajugol^5^  Rehmapicroside^5^  Rehmapicrogenin^6^  hydroferulic acid^6^  aucubin^6^  isoacteoside^6^  acteoside^6^  ethyl β-D-fructofuranoside^6^  eleutheroside C^6^  mannitol^6^  raffinose^6^  stachyose^6^  glutinolic acid^6^  paulownin^6^  monopalmitin^6^  pinellic acid^6^  jio-cerebroside^6^  aeginetic acid 5-O-beta-D-quinovoside^7^  aeginetoyl ajugol 5''-O-beta-D-quinovoside^7^  rehmaionoside A^7^  dihydroxy-beta-ionone^7^  rehmaglutin D^7^  ajugoside^7^  martynoside^8^  leucosceptoside A^8^  5-hydroxymethyl furfural^8^  Tyrosol^8^  Isomartynoside^8^  purpureaside C^8^  jionoside A1^8^  ferulic acid methyl ester^9^  coniferin^9^  leonoside F^9^  echinacoside^9^  diincarvilone A^9^  pterolactam^9^  3-indolecarboxylic acid^9^ | [332427](https://pubchem.ncbi.nlm.nih.gov/compound/332427)  [85382912](https://pubchem.ncbi.nlm.nih.gov/compound/85382912)  [195850](https://pubchem.ncbi.nlm.nih.gov/compound/195850)  [21668724](https://pubchem.ncbi.nlm.nih.gov/compound/21668724)  [13889681](https://pubchem.ncbi.nlm.nih.gov/compound/13889681)  [5281782](https://pubchem.ncbi.nlm.nih.gov/compound/5281782)  [91520](https://pubchem.ncbi.nlm.nih.gov/compound/91520)  [6325127](https://pubchem.ncbi.nlm.nih.gov/compound/6325127)  [14396664](https://pubchem.ncbi.nlm.nih.gov/compound/14396664)  [21637711](https://pubchem.ncbi.nlm.nih.gov/compound/21637711)  [15693863](https://pubchem.ncbi.nlm.nih.gov/compound/15693863)  [14340](https://pubchem.ncbi.nlm.nih.gov/compound/14340)  [91458](https://pubchem.ncbi.nlm.nih.gov/compound/91458)  [6476333](https://pubchem.ncbi.nlm.nih.gov/compound/6476333)  [5281800](https://pubchem.ncbi.nlm.nih.gov/compound/5281800)  [138113708](https://pubchem.ncbi.nlm.nih.gov/compound/138113708)  [9859136](https://pubchem.ncbi.nlm.nih.gov/compound/9859136)  [6251](https://pubchem.ncbi.nlm.nih.gov/compound/6251)  [439242](https://pubchem.ncbi.nlm.nih.gov/compound/439242)  [439531](https://pubchem.ncbi.nlm.nih.gov/compound/439531)  [53248310](https://pubchem.ncbi.nlm.nih.gov/compound/53248310)  [3084131](https://pubchem.ncbi.nlm.nih.gov/compound/3084131)  [14900](https://pubchem.ncbi.nlm.nih.gov/compound/14900)  [9858729](https://pubchem.ncbi.nlm.nih.gov/compound/9858729)  [10169092](https://pubchem.ncbi.nlm.nih.gov/compound/10169092)  [53248311](https://pubchem.ncbi.nlm.nih.gov/compound/53248311)  [101790066](https://pubchem.ncbi.nlm.nih.gov/compound/101790066)  [10023290](https://pubchem.ncbi.nlm.nih.gov/compound/10023290)  [15693868](https://pubchem.ncbi.nlm.nih.gov/compound/15693868)  [5320906](https://pubchem.ncbi.nlm.nih.gov/compound/5320906)  [9865184](https://pubchem.ncbi.nlm.nih.gov/compound/9865184)  [5319292](https://pubchem.ncbi.nlm.nih.gov/compound/5319292)  [10394343](https://pubchem.ncbi.nlm.nih.gov/compound/10394343)  [237332](https://pubchem.ncbi.nlm.nih.gov/compound/237332)  [10393](https://pubchem.ncbi.nlm.nih.gov/compound/10393)  [91895373](https://pubchem.ncbi.nlm.nih.gov/compound/91895373)  [11953944](https://pubchem.ncbi.nlm.nih.gov/compound/11953944)  [6325450](https://pubchem.ncbi.nlm.nih.gov/compound/6325450)  [5357283](https://pubchem.ncbi.nlm.nih.gov/compound/5357283)  [5280372](https://pubchem.ncbi.nlm.nih.gov/compound/5280372)  [57325811](https://pubchem.ncbi.nlm.nih.gov/compound/57325811)  [5281771](https://pubchem.ncbi.nlm.nih.gov/compound/5281771)  [60155233](https://pubchem.ncbi.nlm.nih.gov/compound/60155233)  [181561](https://pubchem.ncbi.nlm.nih.gov/compound/181561)  [69867](https://pubchem.ncbi.nlm.nih.gov/compound/69867) |
| 34. | *Saposhnikovia divaricate*  (44) | Ledebouriellol^10^  Hamaudol^10^  sec-O-glucosylhamaudol^10^  Divaricatol^10^  Cimifugin^10^  prim -O – glucosylcimifugin^10^  5-O-methylvisamminol^10^  4'-O-β-D-glucosyl-5-O-methylvisamminol^10^  Norcimifugin^10^  undulatoside A^10^  wogonin^10^  bergapten^10^  byakangelicin^10^  deltoin^10^  imperatorin^10^  isoimperatorin^10^  isobergapten^10^  marmesin^10^  nodakenetin^10^  oxypeucedanin hydrate^10^  phellopterin^10^  psoralen^10^  xanthotoxin^10^  5-hydroxy-8-methoxypsoralen^10^  Nodakenin^10^  Xanthoarnol^10^  Fraxidin^10^  Isofraxidin^10^  Scopoletin^10^  Umbelliferone^10^  Anomalin^10^  Decursinol^10^  decursinol angelate^10^  praeruptorin B^10^  praeruptorin F^10^  cis-3', 4'-disenecioylkhellactone^10^  (–)-cis-khellactone^10^  Panaxynol^10^  Falcarindiol^10^  (9Z)-1-methoxy9-heptadecene-4,6-diyn-3-ol^10^  Marmesinin^10^  tectochrysin^10^  glyceryl monooleate^10^  8'-epicleomiscosin A^10^ | [5318962](https://pubchem.ncbi.nlm.nih.gov/compound/5318962)  [164722](https://pubchem.ncbi.nlm.nih.gov/compound/164722)  [10478277](https://pubchem.ncbi.nlm.nih.gov/compound/10478277)  [9974111](https://pubchem.ncbi.nlm.nih.gov/compound/9974111)  [441960](https://pubchem.ncbi.nlm.nih.gov/compound/441960)  [14034912](https://pubchem.ncbi.nlm.nih.gov/compound/14034912)  [441970](https://pubchem.ncbi.nlm.nih.gov/compound/441970)  [21670038](https://pubchem.ncbi.nlm.nih.gov/compound/21670038)  [46240156](https://pubchem.ncbi.nlm.nih.gov/compound/46240156)  [5321494](https://pubchem.ncbi.nlm.nih.gov/compound/5321494)  [5281703](https://pubchem.ncbi.nlm.nih.gov/compound/5281703)  [2355](https://pubchem.ncbi.nlm.nih.gov/compound/2355)  [10211](https://pubchem.ncbi.nlm.nih.gov/compound/10211)  [906525](https://pubchem.ncbi.nlm.nih.gov/compound/906525)  [10212](https://pubchem.ncbi.nlm.nih.gov/compound/10212)  [68081](https://pubchem.ncbi.nlm.nih.gov/compound/68081)  [68082](https://pubchem.ncbi.nlm.nih.gov/compound/68082)  [334704](https://pubchem.ncbi.nlm.nih.gov/compound/334704)  [26305](https://pubchem.ncbi.nlm.nih.gov/compound/26305)  [17536](https://pubchem.ncbi.nlm.nih.gov/compound/17536)  [98608](https://pubchem.ncbi.nlm.nih.gov/compound/98608)  [6199](https://pubchem.ncbi.nlm.nih.gov/compound/6199)  [4114](https://pubchem.ncbi.nlm.nih.gov/compound/4114)  [5385192](https://pubchem.ncbi.nlm.nih.gov/compound/5385192)  [73191](https://pubchem.ncbi.nlm.nih.gov/compound/73191)  [11482406](https://pubchem.ncbi.nlm.nih.gov/compound/11482406)  [3083616](https://pubchem.ncbi.nlm.nih.gov/compound/3083616)  [5318565](https://pubchem.ncbi.nlm.nih.gov/compound/5318565)  [5280460](https://pubchem.ncbi.nlm.nih.gov/compound/5280460)  [5281426](https://pubchem.ncbi.nlm.nih.gov/compound/5281426)  [10251869](https://pubchem.ncbi.nlm.nih.gov/compound/10251869)  [442127](https://pubchem.ncbi.nlm.nih.gov/compound/442127)  [776123](https://pubchem.ncbi.nlm.nih.gov/compound/776123)  [5319259](https://pubchem.ncbi.nlm.nih.gov/compound/5319259)  [51668830](https://pubchem.ncbi.nlm.nih.gov/compound/51668830)  [1119128](https://pubchem.ncbi.nlm.nih.gov/compound/1119128)  [455821](https://pubchem.ncbi.nlm.nih.gov/compound/455821)  [5281149](https://pubchem.ncbi.nlm.nih.gov/compound/5281149)  [5281148](https://pubchem.ncbi.nlm.nih.gov/compound/5281148)  [146026404](https://pubchem.ncbi.nlm.nih.gov/compound/146026404)  [216283](https://pubchem.ncbi.nlm.nih.gov/compound/216283)  [5281954](https://pubchem.ncbi.nlm.nih.gov/compound/5281954)  [5283468](https://pubchem.ncbi.nlm.nih.gov/compound/5283468)  [11200016](https://pubchem.ncbi.nlm.nih.gov/compound/11200016) |
| 35. | *Glycyrrhiza uralensis*  (29) | lignoceric acid^11^  licorisoflavan A^11^  methoxyficifolinol^11^  docosanol^11^  betulinic acid^11^  licoricidin^11^  kumatakenin^11^  docosyl caffeate^11^  neoglycyrol^11^  gancaonin H^11^  isoglycyrol^11^  liquiritigenin^11^  lupiwighteone^11^  3R-vestitol^11^  Echinatin^11^  Glycycoumarin^11^  Licoarylcoumarin^11^  7,2',4'-trihydroxy-5-methoxy-3-arylcoumarin^11^  Glicoricone^11^  Isoliquiritigenin^11^  Formononetin^11^  Licoricone^11^  Glycyrin^11^  formononetin-7-O-β-D-glucoside^11^  liquiritin^11^  isoliquiritin apioside^11^  glycyrrhizic acid^11^  liquiritin apioside^11^  hedysarimcoumestan B^11^ | [11197](https://pubchem.ncbi.nlm.nih.gov/compound/11197)  [196831](https://pubchem.ncbi.nlm.nih.gov/compound/196831)  [480872](https://pubchem.ncbi.nlm.nih.gov/compound/480872)  [12620](https://pubchem.ncbi.nlm.nih.gov/compound/12620)  [64971](https://pubchem.ncbi.nlm.nih.gov/compound/64971)  [480865](https://pubchem.ncbi.nlm.nih.gov/compound/480865)  [5318869](https://pubchem.ncbi.nlm.nih.gov/compound/5318869)  [5316952](https://pubchem.ncbi.nlm.nih.gov/compound/5316952)  [5320083](https://pubchem.ncbi.nlm.nih.gov/compound/5320083)  [5481949](https://pubchem.ncbi.nlm.nih.gov/compound/5481949)  [124050](https://pubchem.ncbi.nlm.nih.gov/compound/124050)  [114829](https://pubchem.ncbi.nlm.nih.gov/compound/114829)  [5317480](https://pubchem.ncbi.nlm.nih.gov/compound/5317480)  [439310](https://pubchem.ncbi.nlm.nih.gov/compound/439310)  [6442675](https://pubchem.ncbi.nlm.nih.gov/compound/6442675)  [5317756](https://pubchem.ncbi.nlm.nih.gov/compound/5317756)  [10090416](https://pubchem.ncbi.nlm.nih.gov/compound/10090416)  [25015742](https://pubchem.ncbi.nlm.nih.gov/compound/25015742)  [10361658](https://pubchem.ncbi.nlm.nih.gov/compound/10361658)  [638278](https://pubchem.ncbi.nlm.nih.gov/compound/638278)  [5280378](https://pubchem.ncbi.nlm.nih.gov/compound/5280378)  [5319013](https://pubchem.ncbi.nlm.nih.gov/compound/5319013)  [480787](https://pubchem.ncbi.nlm.nih.gov/compound/480787)  [101350244](https://pubchem.ncbi.nlm.nih.gov/compound/101350244)  [503737](https://pubchem.ncbi.nlm.nih.gov/compound/503737)  [6442433](https://pubchem.ncbi.nlm.nih.gov/compound/6442433)  [14982](https://pubchem.ncbi.nlm.nih.gov/compound/14982)  [10076238](https://pubchem.ncbi.nlm.nih.gov/compound/10076238)  [11558452](https://pubchem.ncbi.nlm.nih.gov/compound/11558452) |
| 36. | *Ziziphus jujuba*  (66) | Mauritine A^12^  Mucronine D^12^  Amphibine H^12^  Nummularine A^12^  Nummularine B^12^  sativanine-B^12^  frangulanine^12^  sativanine F^12^  coclaurine^12^  isoboldine^12^  norisoboldine^12^  asimilobine^12^  sanjoinenine^12^  amphibine-D^12^  sanjoinine-B^12^  sanjoinine-D^12^  sanjoinine-F^12^  Jubanine-A^12^  Jubanine-B^12^  Scutianine-C^12^  Scutianine-D^12^  Jubanine-C^12^  Zizyphine-A^12^ | [11353668](https://pubchem.ncbi.nlm.nih.gov/compound/11353668)  [5373023](https://pubchem.ncbi.nlm.nih.gov/compound/5373023)  [51029223](https://pubchem.ncbi.nlm.nih.gov/compound/51029223)  [131750852](https://pubchem.ncbi.nlm.nih.gov/compound/131750852)  [51017057](https://pubchem.ncbi.nlm.nih.gov/compound/51017057)  [5281595](https://pubchem.ncbi.nlm.nih.gov/compound/5281595)  [5281587](https://pubchem.ncbi.nlm.nih.gov/compound/5281587)  [102156891](https://pubchem.ncbi.nlm.nih.gov/compound/102156891)  [160487](https://pubchem.ncbi.nlm.nih.gov/compound/160487)  [133323](https://pubchem.ncbi.nlm.nih.gov/compound/133323)  [14539911](https://pubchem.ncbi.nlm.nih.gov/compound/14539911)  [160875](https://pubchem.ncbi.nlm.nih.gov/compound/160875)  [14729078](https://pubchem.ncbi.nlm.nih.gov/compound/14729078)  [5318120](https://pubchem.ncbi.nlm.nih.gov/compound/5318120)  [14729076](https://pubchem.ncbi.nlm.nih.gov/compound/14729076)  [44566617](https://pubchem.ncbi.nlm.nih.gov/compound/44566617)  [14729081](https://pubchem.ncbi.nlm.nih.gov/compound/14729081)  [101316794](https://pubchem.ncbi.nlm.nih.gov/compound/101316794)  [101316795](https://pubchem.ncbi.nlm.nih.gov/compound/101316795)  [45268911](https://pubchem.ncbi.nlm.nih.gov/compound/45268911)  [45267179](https://pubchem.ncbi.nlm.nih.gov/compound/45267179)  [131752168](https://pubchem.ncbi.nlm.nih.gov/compound/131752168)  [6324833](https://pubchem.ncbi.nlm.nih.gov/compound/6324833) |
|  |  | 6'''- sinapoylspinosin^12^  6'''-feruloylspinosin^12^  jujuboside A^12^  jujuboside B^12^  jujuboside A1^12^  jujuboside B1^12^  jujuboside C^12^  acetyljujuboside B^12^  protojujuboside A^12^  protojujuboside B^12^  protojujuboside B1^12^  Puerarin^12^  6"'-feruloylisospinosin^12^  Isospinosin^12^  Kaempferol 3-O-robinobioside^12^  Kaempferol 3-O-rutinoside^12^  colubrinic acid^12^  alphitolic acid^12^  zizyberenalic acid^12^  betulinic acid^12^  3-beta-O-(trans-p-coumaroyl)maslinic acid^13^  pomonic acid^13^  2-oxopomolic acid^13^  benthamic acid^13^  terminic acid^13^  traumatic acid^13^  (Z)-4-oxotetradec-5-enoic acid^13^  Magnoflorine^13^ | [101616450](https://pubchem.ncbi.nlm.nih.gov/compound/101616450)  [21597353](https://pubchem.ncbi.nlm.nih.gov/compound/21597353)  [51346169](https://pubchem.ncbi.nlm.nih.gov/compound/51346169)  [24721031](https://pubchem.ncbi.nlm.nih.gov/compound/24721031)  [177396](https://pubchem.ncbi.nlm.nih.gov/compound/177396)  [73156987](https://pubchem.ncbi.nlm.nih.gov/compound/73156987)  [71448946](https://pubchem.ncbi.nlm.nih.gov/compound/71448946)  [73093046](https://pubchem.ncbi.nlm.nih.gov/compound/73093046)  [71448943](https://pubchem.ncbi.nlm.nih.gov/compound/71448943)  [71448944](https://pubchem.ncbi.nlm.nih.gov/compound/71448944)  [71448945](https://pubchem.ncbi.nlm.nih.gov/compound/71448945)  [5281807](https://pubchem.ncbi.nlm.nih.gov/compound/5281807)  [10795344](https://pubchem.ncbi.nlm.nih.gov/compound/10795344)  [11801844](https://pubchem.ncbi.nlm.nih.gov/compound/11801844)  [15944778](https://pubchem.ncbi.nlm.nih.gov/compound/15944778)  [5318767](https://pubchem.ncbi.nlm.nih.gov/compound/5318767)  [21672700](https://pubchem.ncbi.nlm.nih.gov/compound/21672700)  [12305768](https://pubchem.ncbi.nlm.nih.gov/compound/12305768)  [15958448](https://pubchem.ncbi.nlm.nih.gov/compound/15958448)  [64971](https://pubchem.ncbi.nlm.nih.gov/compound/64971)  [14335962](https://pubchem.ncbi.nlm.nih.gov/compound/14335962)  [12314449](https://pubchem.ncbi.nlm.nih.gov/compound/12314449)  [44593379](https://pubchem.ncbi.nlm.nih.gov/compound/44593379)  [3838010](https://pubchem.ncbi.nlm.nih.gov/compound/3838010)  [132568257](https://pubchem.ncbi.nlm.nih.gov/compound/132568257)  [5283028](https://pubchem.ncbi.nlm.nih.gov/compound/5283028)  [132568256](https://pubchem.ncbi.nlm.nih.gov/compound/132568256)  [73337](https://pubchem.ncbi.nlm.nih.gov/compound/73337) |
|  |  | Franganine^14^  Spinosyn^14^  3beta,6beta-stigmast-4-en-3,6-diol^14^  Maslinic acid^14^  Ceanothic acid^14^  Vitexin^14^  Oleamide^14^  Ent-epicatechinoceanothic acid A^14^  Lupeol^14^  2-alpha-hydroxyursolic acid^14^  Ziziphin^14^  Squalene^14^  epiceanothic acid^14^  ceanothenic acid^14^  ursonic acid^14^ | [45270592](https://pubchem.ncbi.nlm.nih.gov/compound/45270592)  [155692](https://pubchem.ncbi.nlm.nih.gov/compound/155692)  [146156493](https://pubchem.ncbi.nlm.nih.gov/compound/146156493)  [73659](https://pubchem.ncbi.nlm.nih.gov/compound/73659)  [161352](https://pubchem.ncbi.nlm.nih.gov/compound/161352)  [5280441](https://pubchem.ncbi.nlm.nih.gov/compound/5280441)  [5283387](https://pubchem.ncbi.nlm.nih.gov/compound/5283387)  [132967447](https://pubchem.ncbi.nlm.nih.gov/compound/132967447)  [259846](https://pubchem.ncbi.nlm.nih.gov/compound/259846)  [6918774](https://pubchem.ncbi.nlm.nih.gov/compound/6918774)  [441957](https://pubchem.ncbi.nlm.nih.gov/compound/441957)  [638072](https://pubchem.ncbi.nlm.nih.gov/compound/638072)  [23631167](https://pubchem.ncbi.nlm.nih.gov/compound/23631167)  [71451218](https://pubchem.ncbi.nlm.nih.gov/compound/71451218)  [9890209](https://pubchem.ncbi.nlm.nih.gov/compound/9890209) |
| 37. | *Pueraria lobata*  (49) | apigenin triacetate^15^  daidzein diacetate^15^  daidzein dimethyl ether^15^  daidzin^15^  puerarin^15^  daidzein^15^  formononetin^15^  puerarol^15^  3'-Hydroxypuerarin^15^  3'-Methoxydaidzein^15^  [3'-](https://pubchem.ncbi.nlm.nih.gov/compound/10527347)methoxydaidzin^15^  6''-O-D-Xylosylpuerarin^15^  biochanin A^15^  4'-Methoxypuerarin^15^  3'-Methoxypuerarin^15^  mirificin^15^  ononin^15^  puerarin-4'-O-glucoside^15^  irisolidone^15^  kaikasaponin I^15^ | [18721](https://pubchem.ncbi.nlm.nih.gov/compound/18721)  [10359753](https://pubchem.ncbi.nlm.nih.gov/compound/10359753)  [136419](https://pubchem.ncbi.nlm.nih.gov/compound/136419)  [107971](https://pubchem.ncbi.nlm.nih.gov/compound/107971)  [5281807](https://pubchem.ncbi.nlm.nih.gov/compound/5281807)  [5281708](https://pubchem.ncbi.nlm.nih.gov/compound/5281708)  [5280378](https://pubchem.ncbi.nlm.nih.gov/compound/5280378)  [44257531](https://pubchem.ncbi.nlm.nih.gov/compound/44257531)  [5748205](https://pubchem.ncbi.nlm.nih.gov/compound/5748205)  [5319422](https://pubchem.ncbi.nlm.nih.gov/compound/5319422)  [10527347](https://pubchem.ncbi.nlm.nih.gov/compound/10527347)  [101776119](https://pubchem.ncbi.nlm.nih.gov/compound/101776119)  [5280373](https://pubchem.ncbi.nlm.nih.gov/compound/5280373)  [5319486](https://pubchem.ncbi.nlm.nih.gov/compound/5319486)  [5319485](https://pubchem.ncbi.nlm.nih.gov/compound/5319485)  [21676217](https://pubchem.ncbi.nlm.nih.gov/compound/21676217)  [442813](https://pubchem.ncbi.nlm.nih.gov/compound/442813)  [44257212](https://pubchem.ncbi.nlm.nih.gov/compound/44257212)  [5281781](https://pubchem.ncbi.nlm.nih.gov/compound/5281781)  [102120183](https://pubchem.ncbi.nlm.nih.gov/compound/102120183) |
|  |  | soyasaponin I^15^  azukisaponin I^15^  baptisiasaponin I^15^  genistein^15^  genistin^15^  glycitein^15^  glycitin^15^  kaikasaponin II^15^  kaikasaponin III^15^  kakkalide^15^  kakkasaponin I^15^  kakkasaponin II^15^  kakkasaponin III^15^  luteolin^15^  phaseoside IV^16^  soyasaponin IV^16^  tectorigenin^16^  tectorigenin-7-O-xylosylglucoside^16^  nicotiflorin^16^  robinin^16^  beta-sitosterol palmitate^16^  lupeone^16^  diisobutyl phthalate^16^  bis(2-ethylhexyl) phthalate^16^  sophoracoumestan A^16^  coumestrol^16^  allantion^16^  Sissotorin^16^  (6S,9R)-roseoside^16^ | [122097](https://pubchem.ncbi.nlm.nih.gov/compound/122097)  [14103656](https://pubchem.ncbi.nlm.nih.gov/compound/14103656)  [102317160](https://pubchem.ncbi.nlm.nih.gov/compound/102317160)  [5280961](https://pubchem.ncbi.nlm.nih.gov/compound/5280961)  [5281377](https://pubchem.ncbi.nlm.nih.gov/compound/5281377)  [5317750](https://pubchem.ncbi.nlm.nih.gov/compound/5317750)  [187808](https://pubchem.ncbi.nlm.nih.gov/compound/187808)  [101538997](https://pubchem.ncbi.nlm.nih.gov/compound/101538997)  [188384](https://pubchem.ncbi.nlm.nih.gov/compound/188384)  [5490351](https://pubchem.ncbi.nlm.nih.gov/compound/5490351)  [100945993](https://pubchem.ncbi.nlm.nih.gov/compound/100945993)  [102500447](https://pubchem.ncbi.nlm.nih.gov/compound/102500447)  [102500448](https://pubchem.ncbi.nlm.nih.gov/compound/102500448)  [5280445](https://pubchem.ncbi.nlm.nih.gov/compound/5280445)  [101679107](https://pubchem.ncbi.nlm.nih.gov/compound/101679107)  [24721354](https://pubchem.ncbi.nlm.nih.gov/compound/24721354)  [5281811](https://pubchem.ncbi.nlm.nih.gov/compound/5281811)  [100968221](https://pubchem.ncbi.nlm.nih.gov/compound/100968221)  [5318767](https://pubchem.ncbi.nlm.nih.gov/compound/5318767)  [5281693](https://pubchem.ncbi.nlm.nih.gov/compound/5281693)  [13747834](https://pubchem.ncbi.nlm.nih.gov/compound/13747834)  [92158](https://pubchem.ncbi.nlm.nih.gov/compound/92158)  [6782](https://pubchem.ncbi.nlm.nih.gov/compound/6782)  [8343](https://pubchem.ncbi.nlm.nih.gov/compound/8343)  [14630492](https://pubchem.ncbi.nlm.nih.gov/compound/14630492)  [5281707](https://pubchem.ncbi.nlm.nih.gov/compound/5281707)  [439714](https://pubchem.ncbi.nlm.nih.gov/compound/439714)  [73407546](https://pubchem.ncbi.nlm.nih.gov/compound/73407546)  [9930064](https://pubchem.ncbi.nlm.nih.gov/compound/9930064) |
| 38. | *Coix lacryma-jobi*  (15) | Coixenolide^17^  γ-Tocopherol^17^  Docosanol^17^  4-Ketopinoresinol^17^  p-Hydroxy benzaldehyde^17^  Tangeretin^17^  Naringenin^17^  Nobiletin^17^  Neolignan^17^  5-Amino-1-(quinolin-8-yl)-1,2,3-triazole-4-carboxamide^18^  Pyridine^18^  Propenamide^18^  N-methoxy-N-methyl-3,4-dihydro-2H-thiopyran6-carboxamide^18^  1,3-dioctanoin^18^  tetradecanoic acid^18^ | [46173943](https://pubchem.ncbi.nlm.nih.gov/compound/46173943)  [14985](https://pubchem.ncbi.nlm.nih.gov/compound/14985)  [12620](https://pubchem.ncbi.nlm.nih.gov/compound/12620)  [44578390](https://pubchem.ncbi.nlm.nih.gov/compound/44578390)  [126](https://pubchem.ncbi.nlm.nih.gov/compound/126)  [68077](https://pubchem.ncbi.nlm.nih.gov/compound/68077)  [932](https://pubchem.ncbi.nlm.nih.gov/compound/932)  [72344](https://pubchem.ncbi.nlm.nih.gov/compound/72344)  [261166](https://pubchem.ncbi.nlm.nih.gov/compound/261166)  [26435789](https://pubchem.ncbi.nlm.nih.gov/compound/26435789)    [1049](https://pubchem.ncbi.nlm.nih.gov/compound/1049)  [6579](https://pubchem.ncbi.nlm.nih.gov/compound/6579)  [24813996](https://pubchem.ncbi.nlm.nih.gov/compound/24813996)  [150911](https://pubchem.ncbi.nlm.nih.gov/compound/150911)  [11005](https://pubchem.ncbi.nlm.nih.gov/compound/11005) |
| 39. | *Rheum palmatum*  (22) | aloe-emodin^19^  chrysophanol^19^  emodin^19^  emodin-6-O-beta-D-glucopyranoside^19^  physcion^19^  physcion-8-O-beta-D-glucopyranoside^19^  rhein^19^  rhein-8-O-β-D-glucopyranoside^19^  palmidin A^19^  palmidin B^19^  palmidin C^19^  rheinoside A^19^  rheinoside C^19^  sennidin C^19^  sennoside A^19^  sennoside B^19^  sennoside C^19^  sennoside D^19^  rhapontigenin^19^  gallic acid 3-O-β-D-glucopyranoside^19^  gallic acid 4-O-β-D-glucopyranoside^19^  coumaroyl-O-galloyl-glucose^19^ | [10207](https://pubchem.ncbi.nlm.nih.gov/compound/10207)  [10208](https://pubchem.ncbi.nlm.nih.gov/compound/10208)  [3220](https://pubchem.ncbi.nlm.nih.gov/compound/3220)  [5317038](https://pubchem.ncbi.nlm.nih.gov/compound/5317038)  [10639](https://pubchem.ncbi.nlm.nih.gov/compound/10639)  [5319323](https://pubchem.ncbi.nlm.nih.gov/compound/5319323)  [10168](https://pubchem.ncbi.nlm.nih.gov/compound/10168)  [146156489](https://pubchem.ncbi.nlm.nih.gov/compound/146156489)  [5320384](https://pubchem.ncbi.nlm.nih.gov/compound/5320384)  [5320385](https://pubchem.ncbi.nlm.nih.gov/compound/5320385)  [5320386](https://pubchem.ncbi.nlm.nih.gov/compound/5320386)  [13888123](https://pubchem.ncbi.nlm.nih.gov/compound/13888123)  [13888128](https://pubchem.ncbi.nlm.nih.gov/compound/13888128)  [5321255](https://pubchem.ncbi.nlm.nih.gov/compound/5321255)  [73111](https://pubchem.ncbi.nlm.nih.gov/compound/73111)  [91440](https://pubchem.ncbi.nlm.nih.gov/compound/91440)  [46173829](https://pubchem.ncbi.nlm.nih.gov/compound/46173829)  [46173830](https://pubchem.ncbi.nlm.nih.gov/compound/46173830)  [5320954](https://pubchem.ncbi.nlm.nih.gov/compound/5320954)  [101683334](https://pubchem.ncbi.nlm.nih.gov/compound/101683334)  [10088114](https://pubchem.ncbi.nlm.nih.gov/compound/10088114)  [146170723](https://pubchem.ncbi.nlm.nih.gov/compound/146170723) |
| 40. | *Uncaria rhynchophylla*  (27) | Hirsutine^20^  Hirsuteine^20^  epi-allo-corynantheine^20^  corynantheidine^20^  dihydrocorynantheine^20^  akuammigine^20^  yohimbine^20^  angustine^20^  angustoline^20^  angustidine^20^  corynoxeine^20^  18,19-dehydrocorynoxinic acid^20^  Isorhynchophylline^20^  18,19-dehydrocorynoxinic acid B^20^  cadambine^20^  3 Alpha-dihydrocadambine^20^  vincoside lactam^20^  strictosidine^20^  strictosamide^20^  6beta-hydroxyursolic acid^20^  uncarinic acid C^20^  uncarinic acid B^20^  uncarinic acid E^20^  trifolin^20^  hyperin^20^  cleomiscosin B^20^  cleomiscosin D^20^ | [3037884](https://pubchem.ncbi.nlm.nih.gov/compound/3037884)  [3037151](https://pubchem.ncbi.nlm.nih.gov/compound/3037151)  [101603230](https://pubchem.ncbi.nlm.nih.gov/compound/101603230)  [3000341](https://pubchem.ncbi.nlm.nih.gov/compound/3000341)  [3039336](https://pubchem.ncbi.nlm.nih.gov/compound/3039336)  [1268096](https://pubchem.ncbi.nlm.nih.gov/compound/1268096)  [8969](https://pubchem.ncbi.nlm.nih.gov/compound/8969)  [441983](https://pubchem.ncbi.nlm.nih.gov/compound/441983)  [3084765](https://pubchem.ncbi.nlm.nih.gov/compound/3084765)  [3084770](https://pubchem.ncbi.nlm.nih.gov/compound/3084770)  [44568160](https://pubchem.ncbi.nlm.nih.gov/compound/44568160)  [24970642](https://pubchem.ncbi.nlm.nih.gov/compound/24970642)  [3037048](https://pubchem.ncbi.nlm.nih.gov/compound/3037048)  [24970641](https://pubchem.ncbi.nlm.nih.gov/compound/24970641)  [398038](https://pubchem.ncbi.nlm.nih.gov/compound/398038)  [162138](https://pubchem.ncbi.nlm.nih.gov/compound/162138)  [44567645](https://pubchem.ncbi.nlm.nih.gov/compound/44567645)  [161336](https://pubchem.ncbi.nlm.nih.gov/compound/161336)  [10345799](https://pubchem.ncbi.nlm.nih.gov/compound/10345799)  [20055642](https://pubchem.ncbi.nlm.nih.gov/compound/20055642)  [44583694](https://pubchem.ncbi.nlm.nih.gov/compound/44583694)  [100967916](https://pubchem.ncbi.nlm.nih.gov/compound/100967916)  [10746421](https://pubchem.ncbi.nlm.nih.gov/compound/10746421)  [5282149](https://pubchem.ncbi.nlm.nih.gov/compound/5282149)  [5281643](https://pubchem.ncbi.nlm.nih.gov/compound/5281643)  [156875](https://pubchem.ncbi.nlm.nih.gov/compound/156875)  [13965876](https://pubchem.ncbi.nlm.nih.gov/compound/13965876) |

**References:**

1. Wu, Y. Y. *et al.* Chemical constituents from the tubers of Pinellia ternata (Araceae) and their chemotaxonomic interest. *Biochem. Syst. Ecol.* **62**, 236–240 (2015).

2. Ji, X., Huang, B., Wang, G. & Zhang, C. The ethnobotanical, phytochemical and pharmacological profile of the genus Pinellia. *Fitoterapia* **93**, 1–17 (2014).

3. Zhang, L. *et al.* Platycodon grandiflorus - An Ethnopharmacological, phytochemical and pharmacological review. *J. Ethnopharmacol.* **164**, 147–161 (2015).

4. Ji, M. *et al.* foods The Pharmacological E ff ects and Health Benefits of Platycodon grandiflorus — A Medicine Food Homology Species.

5. Feng, W. *et al.* A new megastigmane from fresh roots of Rehmannia glutinosa. *Acta Pharm. Sin. B* **3**, 333–336 (2013).

6. Lee, S. Y., Yean, M. H., Kim, J. S., Lee, J. & Kang, S. S. Phytochemical Studies on Rehmanniae Radix. **42**, 127–137 (2011).

7. Anh, N. T. H., Sung, T. V, Franke, K. & Wessjohann, L. A. Phytochemical studies of Rehmannia glutinosa rhizomes. **58**, 593–595 (2003).

8. Li, X. *et al.* [Chemical constituents from Rehmannia glutinosa]. *Zhongguo Zhong Yao Za Zhi* **36**, 3125–3129 (2011).

9. Feng, W. S. *et al.* Study on chemical constituents of immunosuppressive parts from the roots of Rehmannia glutinosa. *Chinese Pharm. J.* **49**, 1496–1502 (2014).

10. Ethnopharmacological, A. & Review, P. Saposhnikovia divaricata — An Ethnopharmacological, Phytochemical and Pharmacological Review. **26**, 873–880 (2020).

11. LIU, Y. *et al.* Studies on chemical constituents on roots of Glycyrrhiza uralensis. *Chinese J. Pharm. Anal.* **31**, 1251–1255 (2011).

12. Journal, I. & Sciences, L. Available online at http // www.ijrdpl.com Review Article ZIZIPHUS JUJUBA : A PHYTOPHARMACOLOGICAL REVIEW Preeti * and Shalini Tripathi. **3**, 959–966 (2014).

13. Bai, L. *et al.* Chemical characterization of the main bioactive constituents from fruits of Ziziphus jujuba. *Food Funct.* **7**, 2870–2877 (2016).

14. Lv, S. L. Y., Yu, Z. T., Xu, Z. H., Cui, D. Z. C. & Sun, H. L. H. *Ziziphus jujuba Mill ., a plant used as medicinal food : a review of its phytochemistry , pharmacology , quality control and future research*. *Phytochemistry Reviews* vol. 1 (Springer Netherlands, 2020).

15. Sanjappa, W., Flavonoid, P., Tungmunnithum, D., Intharuksa, A. & Sasaki, Y. Traditional Uses and Potential Biological Activities. (2020).

16. Li, G., Zhang, Q. & Wang, Y. [Chemical constituents from roots of Pueraria lobata]. *Zhongguo Zhong Yao Za Zhi* **35**, 3156–3160 (2010).

17. Devaraj, R. D., Jeepipalli, S. P. K. & Xu, B. Phytochemistry and health promoting effects of Job’s tears (Coix lacryma-jobi) - A critical review. *Food Biosci.* 100537 (2020) doi:10.1016/j.fbio.2020.100537.

18. Setya, D. *et al.* Phytochemical Screening and Antibacterial Activity Coix lacryma-jobi Oil. **2818**, 100–106 (2020).

19. He, J. *et al.* Chemistry, pharmacology and processing method of rhubarb (﻿Rheum﻿ species): a review. 42–50 (2019) doi:10.31665/JFB.2019.8205.

20. Zhang, Q., Zhao, J. J., Xu, J., Feng, F. & Qu, W. Medicinal uses , phytochemistry and pharmacology of the genus Uncaria. *J. Ethnopharmacol.* 1–33 (2015) doi:10.1016/j.jep.2015.06.011.
