# Supplementary material for: An in silico approach to develop potential therapies against Middle East Respiratory Syndrome Coronavirus (MERS-CoV)
Source: Heliyon. 2024 Feb 9;10(4):e25837. doi: 10.1016/j.heliyon.2024.e25837 (PMC10877303; doi:10.1016/j.heliyon.2024.e25837)
Supplement: Multimedia component 5 [file mmc5.docx]

| **SL.**  **No.** | **Plant Name** | **Compounds** | **CID** |
| --- | --- | --- | --- |
| 41. | *Forsythia suspensa*  (90) | (+)-pinoresinol^1^  (+)-phillygenin^1^  Phillyrin^1^  (+) epipinoresinol^1^  8- hydroxypinoresinol^1^  benzenebutanoic acid^1^  Arctigenin^1^  (−) dimethylmatairesinol^1^  Arctiin^1^  (–)- matairesinoside^1^  Olivil^1^  (+) lariciresinol^1^  forsythialan A^1^  forsythialan B^1^  (–)-egenine^1^  (–)-bicuculline^1^  Rutaecarpine^1^  p-hydroxyphenylacetic acid^1^  suspenolic acid^1^  Trans-nerolidol^1^  2,4-di-tert-butylphenol^1^  estragole^1^  p-tyrosol^1^  hydroxytyrosol^1^  Sasanquin^1^  tannic acid^1^  esculetin^1^ | [73399](https://pubchem.ncbi.nlm.nih.gov/compound/73399)  [4166098](https://pubchem.ncbi.nlm.nih.gov/compound/4166098)  [101712](https://pubchem.ncbi.nlm.nih.gov/compound/101712)  [637584](https://pubchem.ncbi.nlm.nih.gov/compound/637584)  [3010930](https://pubchem.ncbi.nlm.nih.gov/compound/3010930)  [4775](https://pubchem.ncbi.nlm.nih.gov/compound/4775)  [64981](https://pubchem.ncbi.nlm.nih.gov/compound/64981)  [1286](https://pubchem.ncbi.nlm.nih.gov/compound/1286)  [100528](https://pubchem.ncbi.nlm.nih.gov/compound/100528)  [486612](https://pubchem.ncbi.nlm.nih.gov/compound/486612)  [5273570](https://pubchem.ncbi.nlm.nih.gov/compound/5273570)  [332427](https://pubchem.ncbi.nlm.nih.gov/compound/332427)  [44453412](https://pubchem.ncbi.nlm.nih.gov/compound/44453412)  [44453332](https://pubchem.ncbi.nlm.nih.gov/compound/44453332)  [189685](https://pubchem.ncbi.nlm.nih.gov/compound/189685)  [185838](https://pubchem.ncbi.nlm.nih.gov/compound/185838)  [65752](https://pubchem.ncbi.nlm.nih.gov/compound/65752)  [127](https://pubchem.ncbi.nlm.nih.gov/compound/127)  [10774324](https://pubchem.ncbi.nlm.nih.gov/compound/10774324)  [5284507](https://pubchem.ncbi.nlm.nih.gov/compound/5284507)  [7311](https://pubchem.ncbi.nlm.nih.gov/compound/7311)  [8815](https://pubchem.ncbi.nlm.nih.gov/compound/8815)  [10393](https://pubchem.ncbi.nlm.nih.gov/compound/10393)  [82755](https://pubchem.ncbi.nlm.nih.gov/compound/82755)  [5317538](https://pubchem.ncbi.nlm.nih.gov/compound/5317538)  [16129778](https://pubchem.ncbi.nlm.nih.gov/compound/16129778)  [5281416](https://pubchem.ncbi.nlm.nih.gov/compound/5281416) |
|  |  | cedrusin^1^  glochidioboside^1^  icariside E4^1^  Isolariciresinol^1^  (+)-isoolivil^1^  Forsythiaside (forsythoside A)^1^  forsythoside D^1^  forsythoside E^1^  salidroside^1^  forsythoside I^1^  calceolarioside A^1^  Suspensaside A^1^  R-suspensaside^1^  S-suspensaside^1^  forsythoside B^1^  forsythoside F^1^  forsythoside G^1^  forsythoside H^1^  calceolarioside B^1^  plantainoside A^1^  plantainoside B^1^  calceolarioside C^1^  Wogonin-7-O-glucoside^1^  Hesperidin^1^  Hyperin^1^  taraxasterol acetate^1^  nigaichigoside F1^1^  quadranoside IV^1^  esculentic acid^1^ | [11210164](https://pubchem.ncbi.nlm.nih.gov/compound/11210164)  [24982202](https://pubchem.ncbi.nlm.nih.gov/compound/24982202)  [21589939](https://pubchem.ncbi.nlm.nih.gov/compound/21589939)  [160521](https://pubchem.ncbi.nlm.nih.gov/compound/160521)  [5316262](https://pubchem.ncbi.nlm.nih.gov/compound/5316262)  [5281773](https://pubchem.ncbi.nlm.nih.gov/compound/5281773)  [24721571](https://pubchem.ncbi.nlm.nih.gov/compound/24721571)  [69634125](https://pubchem.ncbi.nlm.nih.gov/compound/69634125)  [159278](https://pubchem.ncbi.nlm.nih.gov/compound/159278)  [23958169](https://pubchem.ncbi.nlm.nih.gov/compound/23958169)  [5273566](https://pubchem.ncbi.nlm.nih.gov/compound/5273566)  [132550846](https://pubchem.ncbi.nlm.nih.gov/compound/132550846)  [102228838](https://pubchem.ncbi.nlm.nih.gov/compound/102228838)  [102228839](https://pubchem.ncbi.nlm.nih.gov/compound/102228839)  [23928102](https://pubchem.ncbi.nlm.nih.gov/compound/23928102)  [6442994](https://pubchem.ncbi.nlm.nih.gov/compound/6442994)  [101231533](https://pubchem.ncbi.nlm.nih.gov/compound/101231533)  [129449684](https://pubchem.ncbi.nlm.nih.gov/compound/129449684)  [5273567](https://pubchem.ncbi.nlm.nih.gov/compound/5273567)  [5320625](https://pubchem.ncbi.nlm.nih.gov/compound/5320625)  [9847922](https://pubchem.ncbi.nlm.nih.gov/compound/9847922)  [45360240](https://pubchem.ncbi.nlm.nih.gov/compound/45360240)  [51136398](https://pubchem.ncbi.nlm.nih.gov/compound/51136398)  [10621](https://pubchem.ncbi.nlm.nih.gov/compound/10621)  [5281643](https://pubchem.ncbi.nlm.nih.gov/compound/5281643)  [13889352](https://pubchem.ncbi.nlm.nih.gov/compound/13889352)  [16118969](https://pubchem.ncbi.nlm.nih.gov/compound/16118969)  [10372074](https://pubchem.ncbi.nlm.nih.gov/compound/10372074)  [9898760](https://pubchem.ncbi.nlm.nih.gov/compound/9898760) |
|  |  | corosolic acid^1^  onjisaponin F^1^  arjunglucoside I^1^  arjunglucoside II^1^  hovenic acid^1^  alphitolic acid^1^  isobauerenyl acetate^1^  Ocotillone^1^  garcinielliptone Q^1^  agatholic acid^1^  3- oxoanticopalic acid^1^  haplopappic acid^1^  dehydropinifolic acid^1^  adoxosidic acid^1^  Alpha-thujene^1^  sabinene^1^  γ-terpinene^1^  terpinolene^1^  (+)-carene^1^  α-ocimene^1^  terpinen-4-ol^1^  p-cymen-8-ol^1^  trans-carveol^1^  (-)-myrtenal^1^  cornoside^1^  rengyolone^1^  forsythenside A^1^  forsythenside B^1^  Rengyoside B^1^  (6S,9R)- roseoside^1^  Rengyol^1^  Rengyoxide^1^  rengyoside A^1^  rengynic acid^1^ | [6918774](https://pubchem.ncbi.nlm.nih.gov/compound/6918774)  [10701737](https://pubchem.ncbi.nlm.nih.gov/compound/10701737)  [14658050](https://pubchem.ncbi.nlm.nih.gov/compound/14658050)  [52951052](https://pubchem.ncbi.nlm.nih.gov/compound/52951052)  [70698002](https://pubchem.ncbi.nlm.nih.gov/compound/70698002)  [12305768](https://pubchem.ncbi.nlm.nih.gov/compound/12305768)  [181096](https://pubchem.ncbi.nlm.nih.gov/compound/181096)  [12313665](https://pubchem.ncbi.nlm.nih.gov/compound/12313665)  [102452579](https://pubchem.ncbi.nlm.nih.gov/compound/102452579)  [101306703](https://pubchem.ncbi.nlm.nih.gov/compound/101306703)  [13858184](https://pubchem.ncbi.nlm.nih.gov/compound/13858184)  [102117108](https://pubchem.ncbi.nlm.nih.gov/compound/102117108)  [22568711](https://pubchem.ncbi.nlm.nih.gov/compound/22568711)  [13892717](https://pubchem.ncbi.nlm.nih.gov/compound/13892717)  [17868](https://pubchem.ncbi.nlm.nih.gov/compound/17868)  [18818](https://pubchem.ncbi.nlm.nih.gov/compound/18818)  [7461](https://pubchem.ncbi.nlm.nih.gov/compound/7461)  [11463](https://pubchem.ncbi.nlm.nih.gov/compound/11463)  [442461](https://pubchem.ncbi.nlm.nih.gov/compound/442461)  [5320249](https://pubchem.ncbi.nlm.nih.gov/compound/5320249)  [11230](https://pubchem.ncbi.nlm.nih.gov/compound/11230)  [14529](https://pubchem.ncbi.nlm.nih.gov/compound/14529)  [94221](https://pubchem.ncbi.nlm.nih.gov/compound/94221)  [1201529](https://pubchem.ncbi.nlm.nih.gov/compound/1201529)  [11809239](https://pubchem.ncbi.nlm.nih.gov/compound/11809239)  [10725564](https://pubchem.ncbi.nlm.nih.gov/compound/10725564)  [11797786](https://pubchem.ncbi.nlm.nih.gov/compound/11797786)  [10576163](https://pubchem.ncbi.nlm.nih.gov/compound/10576163)  [11045420](https://pubchem.ncbi.nlm.nih.gov/compound/11045420)  [129316932](https://pubchem.ncbi.nlm.nih.gov/compound/129316932)  [363707](https://pubchem.ncbi.nlm.nih.gov/compound/363707)  [14353410](https://pubchem.ncbi.nlm.nih.gov/compound/14353410)  [10958239](https://pubchem.ncbi.nlm.nih.gov/compound/10958239)  [54033324](https://pubchem.ncbi.nlm.nih.gov/compound/54033324) |
| 42. | *Alisma orientale*  (60) | Alisol A^2^  Alisol B^2^  Alisol C^2^  Alisol E (epi-alisol A)^2^  Alisol F^2^  Alisol G ( 25-Anhydro-alisol A)^2^  Alisol O^2^  Alisol P^2^  Alisol A 23-acetate^2^  Alisol B 23-acetate^2^  Alisol C 23-acetate^2^  Alisol E 23-acetate^2^  Alisol J 23-acetate^2^  Alisol K 23-acetate^2^  Alisol L 23-acetate^2^  Alisol M 23-acetate^2^  Alisol N 23-acetate^2^  Alisol A 24-acetate^2^  Alismaketone A 23-acetate^2^  Alismaketone B 23-acetate^2^  Alismaketone C 23-acetate^2^  Alismalactone 23-acetate^2^  Alisolide^2^  11-Deoxy-alisol B 23-acetate^2^  11-Deoxy-alisol C 23-acetate^2^ | [15558616](https://pubchem.ncbi.nlm.nih.gov/compound/15558616)  [15558620](https://pubchem.ncbi.nlm.nih.gov/compound/15558620)  [46173914](https://pubchem.ncbi.nlm.nih.gov/compound/46173914)  [101297679](https://pubchem.ncbi.nlm.nih.gov/compound/101297679)  [76310822](https://pubchem.ncbi.nlm.nih.gov/compound/76310822)  [76314443](https://pubchem.ncbi.nlm.nih.gov/compound/76314443)  [102004738](https://pubchem.ncbi.nlm.nih.gov/compound/102004738)  [101840124](https://pubchem.ncbi.nlm.nih.gov/compound/101840124)  [70690607](https://pubchem.ncbi.nlm.nih.gov/compound/70690607)  [14036811](https://pubchem.ncbi.nlm.nih.gov/compound/14036811)  [14036813](https://pubchem.ncbi.nlm.nih.gov/compound/14036813)  [101661272](https://pubchem.ncbi.nlm.nih.gov/compound/101661272)  [100936564](https://pubchem.ncbi.nlm.nih.gov/compound/100936564)  [100936565](https://pubchem.ncbi.nlm.nih.gov/compound/100936565)  [100936566](https://pubchem.ncbi.nlm.nih.gov/compound/100936566)  [70688546](https://pubchem.ncbi.nlm.nih.gov/compound/70688546)  [100936567](https://pubchem.ncbi.nlm.nih.gov/compound/100936567)  [76336194](https://pubchem.ncbi.nlm.nih.gov/compound/76336194)  [11800363](https://pubchem.ncbi.nlm.nih.gov/compound/11800363)  [100941986](https://pubchem.ncbi.nlm.nih.gov/compound/100941986)  [100941987](https://pubchem.ncbi.nlm.nih.gov/compound/100941987)  [101701562](https://pubchem.ncbi.nlm.nih.gov/compound/101701562)  [101840122](https://pubchem.ncbi.nlm.nih.gov/compound/101840122)  [101661275](https://pubchem.ncbi.nlm.nih.gov/compound/101661275)  [101664412](https://pubchem.ncbi.nlm.nih.gov/compound/101664412) |
|  |  | 13β,17β-Epoxy-alisol B^2^  25-Anhydro-alisol A 11-acetate^2^  25-Anhydro-alisol F^2^  16-Oxo-alisol A^2^  16,23-Oxido-alisol B^2^  25-O-methyl-alisol A^2^  Orientalol E^2^  Orientalol F^2^  Orientanone^2^  Sulfoorientalol B^2^  Sulfoorientalol C^2^  Sulfoorientalol D^2^  Alismorientol A^2^  Alismorientol B^2^  Alismol^2^  Alismoxide^2^  Clovandiol^2^  Germacrene D^2^  Germacrene C^2^ | [101664542](https://pubchem.ncbi.nlm.nih.gov/compound/101664542)  [5318945](https://pubchem.ncbi.nlm.nih.gov/compound/5318945)  [102004739](https://pubchem.ncbi.nlm.nih.gov/compound/102004739)  [9983614](https://pubchem.ncbi.nlm.nih.gov/compound/9983614)  [9847547](https://pubchem.ncbi.nlm.nih.gov/compound/9847547)  [102004721](https://pubchem.ncbi.nlm.nih.gov/compound/102004721)  [637282](https://pubchem.ncbi.nlm.nih.gov/compound/637282)  [11020886](https://pubchem.ncbi.nlm.nih.gov/compound/11020886)  [11727780](https://pubchem.ncbi.nlm.nih.gov/compound/11727780)  [5321551](https://pubchem.ncbi.nlm.nih.gov/compound/5321551)  [5321552](https://pubchem.ncbi.nlm.nih.gov/compound/5321552)  [146033487](https://pubchem.ncbi.nlm.nih.gov/compound/146033487)  [16742799](https://pubchem.ncbi.nlm.nih.gov/compound/16742799)  [16742798](https://pubchem.ncbi.nlm.nih.gov/compound/16742798)  [5318916](https://pubchem.ncbi.nlm.nih.gov/compound/5318916)  [10988340](https://pubchem.ncbi.nlm.nih.gov/compound/10988340)  [76319362](https://pubchem.ncbi.nlm.nih.gov/compound/76319362)  [5317570](https://pubchem.ncbi.nlm.nih.gov/compound/5317570)  [25244915](https://pubchem.ncbi.nlm.nih.gov/compound/25244915) |
|  |  | Oplopanone^2^  Oriediterpenol^2^  Oriediterpenoside^2^  Robustaflavone^2^  Amentoflavone^2^  Calycosin^2^  7-Hydroxy-coumarin^2^  Emodin^2^  Falcalindiol^2^  Isoimperatorin^2^  Seselin^2^  Dulcitol^2^  Stachyose^2^  Verbascose^2^  Manninotriose^2^  Verbascotetraose^2^ | [10466745](https://pubchem.ncbi.nlm.nih.gov/compound/10466745)  [5320304](https://pubchem.ncbi.nlm.nih.gov/compound/5320304)  [6325764](https://pubchem.ncbi.nlm.nih.gov/compound/6325764)  [5281694](https://pubchem.ncbi.nlm.nih.gov/compound/5281694)  [5281600](https://pubchem.ncbi.nlm.nih.gov/compound/5281600)  [5280448](https://pubchem.ncbi.nlm.nih.gov/compound/5280448)  [5281426](https://pubchem.ncbi.nlm.nih.gov/compound/5281426)  [3220](https://pubchem.ncbi.nlm.nih.gov/compound/3220)  [6436239](https://pubchem.ncbi.nlm.nih.gov/compound/6436239)  [68081](https://pubchem.ncbi.nlm.nih.gov/compound/68081)  [68229](https://pubchem.ncbi.nlm.nih.gov/compound/68229)  [11850](https://pubchem.ncbi.nlm.nih.gov/compound/11850)  [439531](https://pubchem.ncbi.nlm.nih.gov/compound/439531)  [441434](https://pubchem.ncbi.nlm.nih.gov/compound/441434)  [5461026](https://pubchem.ncbi.nlm.nih.gov/compound/5461026)  [102273024](https://pubchem.ncbi.nlm.nih.gov/compound/102273024) |
| 43. | *Amomum xanthioides*  (43) | (+)-5-endo-hydroxycamphor^3^  protocatechuic acid methyl ester^3^  betulabuside A ^3^  hedychiol A^4^  pygmol^4^  dihydroyashabushiketol^4^  Tricyclene^5^  o-Cymene^5^  1,8-Cineole^5^  (E)-β-Ocimene^5^  α-Terpinolene^5^  Camphor^5^  2,6-Dimethyl-2,4,6-octatriene^5^  Terpinen-4-ol^5^  Fenchyl acetate^5^  Bornyl acetate^5^  Bicycloelemene^5^  Dodecamethyl-cyclohexasiloxane^5^  α-Cubebene^5^  β-Cubebene^5^ | [9543191](https://pubchem.ncbi.nlm.nih.gov/compound/9543191)  [287064](https://pubchem.ncbi.nlm.nih.gov/compound/287064)  [14484636](https://pubchem.ncbi.nlm.nih.gov/compound/14484636)  [10105633](https://pubchem.ncbi.nlm.nih.gov/compound/10105633)  [14167388](https://pubchem.ncbi.nlm.nih.gov/compound/14167388)  [10265808](https://pubchem.ncbi.nlm.nih.gov/compound/10265808)  [79035](https://pubchem.ncbi.nlm.nih.gov/compound/79035)  [10703](https://pubchem.ncbi.nlm.nih.gov/compound/10703)  [2758](https://pubchem.ncbi.nlm.nih.gov/compound/2758)  [5281553](https://pubchem.ncbi.nlm.nih.gov/compound/5281553)  [11463](https://pubchem.ncbi.nlm.nih.gov/compound/11463)  [2537](https://pubchem.ncbi.nlm.nih.gov/compound/2537)  [5368821](https://pubchem.ncbi.nlm.nih.gov/compound/5368821)  [11230](https://pubchem.ncbi.nlm.nih.gov/compound/11230)  [107217](https://pubchem.ncbi.nlm.nih.gov/compound/107217)  [6448](https://pubchem.ncbi.nlm.nih.gov/compound/6448)  [56842786](https://pubchem.ncbi.nlm.nih.gov/compound/56842786)  [10911](https://pubchem.ncbi.nlm.nih.gov/compound/10911)  [442359](https://pubchem.ncbi.nlm.nih.gov/compound/442359)  [93081](https://pubchem.ncbi.nlm.nih.gov/compound/93081) |
|  |  | Alpha-Gurjunene^5^  Beta-Gurjunene^5^  Aromadendrene^5^  γ-Gurjunene^5^  Germacrene D^5^  α-Amorphene^5^  Beta-Selinene^5^  Epi-bicyclosesquiphellandrene^5^  Cadina-1,4-diene^5^  Bicyclogermacrene^5^  Endo-1-bourbonanol^5^  β-Maaliene^5^  Nerolidol^5^  Spathulenol^5^  Caryophyllene oxide^5^  Guaiol^5^  Beta-Himachalene^5^  τ-Muurolol^5^  Bulnesol^5^  Farnesol^5^  Farnesyl acetate^5^  Benzyl benzoate^5^  Benzyl salicylate^5^ | [15560276](https://pubchem.ncbi.nlm.nih.gov/compound/15560276)  [6450812](https://pubchem.ncbi.nlm.nih.gov/compound/6450812)  [91354](https://pubchem.ncbi.nlm.nih.gov/compound/91354)  [15560285](https://pubchem.ncbi.nlm.nih.gov/compound/15560285)  [5317570](https://pubchem.ncbi.nlm.nih.gov/compound/5317570)  [12306052](https://pubchem.ncbi.nlm.nih.gov/compound/12306052)  [442393](https://pubchem.ncbi.nlm.nih.gov/compound/442393)  [91747125](https://pubchem.ncbi.nlm.nih.gov/compound/91747125)  [6427091](https://pubchem.ncbi.nlm.nih.gov/compound/6427091)  [13894537](https://pubchem.ncbi.nlm.nih.gov/compound/13894537)  [12301996](https://pubchem.ncbi.nlm.nih.gov/compound/12301996)  [101596917](https://pubchem.ncbi.nlm.nih.gov/compound/101596917)  [5284507](https://pubchem.ncbi.nlm.nih.gov/compound/5284507)  [92231](https://pubchem.ncbi.nlm.nih.gov/compound/92231)  [1742210](https://pubchem.ncbi.nlm.nih.gov/compound/1742210)  [227829](https://pubchem.ncbi.nlm.nih.gov/compound/227829)  [11586487](https://pubchem.ncbi.nlm.nih.gov/compound/11586487)  [51394521](https://pubchem.ncbi.nlm.nih.gov/compound/51394521)  [90785](https://pubchem.ncbi.nlm.nih.gov/compound/90785)  [445070](https://pubchem.ncbi.nlm.nih.gov/compound/445070)  [94403](https://pubchem.ncbi.nlm.nih.gov/compound/94403)  [2345](https://pubchem.ncbi.nlm.nih.gov/compound/2345)  [8363](https://pubchem.ncbi.nlm.nih.gov/compound/8363) |
| 44. | *Areca catechu*  (34) | Arecoline^6^  Arecaidine^6^  Arecolidine^6^  methyl nicotinate^6^  ethyl nicotinate^6^  ethyl N-methylpiperidine-3-carboxylate^6^  isoguvacine^6^  homoarecoline^6^  isorhamnetin^6^  chrysoeriol^6^  liquiritigenin^6^  jacareubin^6^  procyanidin A1^6^  procyanidin B1^6^  procyanidin B2^6^  arecatannin A1^6^  arecatannin B1^6^  arecatannin C1^6^  arecatannin A2^6^  arecatannin A3^6^  arecatannin B2^6^  ursonic acid^6^  Arborinol^6^  arborinol methyl ether^6^  fernenol^6^  arundoin^6^  cycloartenol^6^  chrysophanol^6^  physcion^6^  p-hydroxybenzoic acid^6^  epoxyconiferyl alcohol^6^  isovanillic acid^6^  de-O-methyllasiodiplodin^6^  cyclo-(Leu-Tyr)^6^ | [2230](https://pubchem.ncbi.nlm.nih.gov/compound/2230)  [10355](https://pubchem.ncbi.nlm.nih.gov/compound/10355)  [5319882](https://pubchem.ncbi.nlm.nih.gov/compound/5319882)  [7151](https://pubchem.ncbi.nlm.nih.gov/compound/7151)  [69188](https://pubchem.ncbi.nlm.nih.gov/compound/69188)  [97981](https://pubchem.ncbi.nlm.nih.gov/compound/97981)  [3765](https://pubchem.ncbi.nlm.nih.gov/compound/3765)  [34167](https://pubchem.ncbi.nlm.nih.gov/compound/34167)  [5281654](https://pubchem.ncbi.nlm.nih.gov/compound/5281654)  [5280666](https://pubchem.ncbi.nlm.nih.gov/compound/5280666)  [114829](https://pubchem.ncbi.nlm.nih.gov/compound/114829)  [5281644](https://pubchem.ncbi.nlm.nih.gov/compound/5281644)  [9872976](https://pubchem.ncbi.nlm.nih.gov/compound/9872976)  [11250133](https://pubchem.ncbi.nlm.nih.gov/compound/11250133)  [122738](https://pubchem.ncbi.nlm.nih.gov/compound/122738)  [13752000](https://pubchem.ncbi.nlm.nih.gov/compound/13752000)  [14237657](https://pubchem.ncbi.nlm.nih.gov/compound/14237657)  [9876038](https://pubchem.ncbi.nlm.nih.gov/compound/9876038)  [16142155](https://pubchem.ncbi.nlm.nih.gov/compound/16142155)  [16201011](https://pubchem.ncbi.nlm.nih.gov/compound/16201011)  [71448962](https://pubchem.ncbi.nlm.nih.gov/compound/71448962)  [9890209](https://pubchem.ncbi.nlm.nih.gov/compound/9890209)  [12305177](https://pubchem.ncbi.nlm.nih.gov/compound/12305177)  [101600057](https://pubchem.ncbi.nlm.nih.gov/compound/101600057)  [12305178](https://pubchem.ncbi.nlm.nih.gov/compound/12305178)  [12308619](https://pubchem.ncbi.nlm.nih.gov/compound/12308619)  [92110](https://pubchem.ncbi.nlm.nih.gov/compound/92110)  [10208](https://pubchem.ncbi.nlm.nih.gov/compound/10208)  [10639](https://pubchem.ncbi.nlm.nih.gov/compound/10639)  [135](https://pubchem.ncbi.nlm.nih.gov/compound/135)  [57403796](https://pubchem.ncbi.nlm.nih.gov/compound/57403796)  [12575](https://pubchem.ncbi.nlm.nih.gov/compound/12575)  [14562693](https://pubchem.ncbi.nlm.nih.gov/compound/14562693)  [15550385](https://pubchem.ncbi.nlm.nih.gov/compound/15550385) |
| 45. | *Cannabis sativa*  (89) | Cannabisol^7^  Cannabigerol^7^  α-cadinyl-cannabigerolate^7^  carmagerol^7^  Sesquicannabigerol^7^  Cannabichromene^7^  Cannabidiol^7^  cannabidiolic acid^7^  cannabidiol monomethyl ether^7^  (-)-cannabidivarin^7^  cannabidivarinic acid^7^  Cannabinodiol^7^  cannabielsoic acid A^7^  cannabielsoin^7^  cannabielsoic acid B^7^  Cannabicyclol^7^  cannabicyclolic acid^7^  8-hydroxycannabinolic acid A^7^  8-hydroxycannabinol^7^ | [102487751](https://pubchem.ncbi.nlm.nih.gov/compound/102487751)  [5315659](https://pubchem.ncbi.nlm.nih.gov/compound/5315659)  [24862530](https://pubchem.ncbi.nlm.nih.gov/compound/24862530)  [44586785](https://pubchem.ncbi.nlm.nih.gov/compound/44586785)  [54669855](https://pubchem.ncbi.nlm.nih.gov/compound/54669855)  [30219](https://pubchem.ncbi.nlm.nih.gov/compound/30219)  [644019](https://pubchem.ncbi.nlm.nih.gov/compound/644019)  [160570](https://pubchem.ncbi.nlm.nih.gov/compound/160570)  [164905](https://pubchem.ncbi.nlm.nih.gov/compound/164905)  [11601669](https://pubchem.ncbi.nlm.nih.gov/compound/11601669)  [59444387](https://pubchem.ncbi.nlm.nih.gov/compound/59444387)  [11551346](https://pubchem.ncbi.nlm.nih.gov/compound/11551346)  [59444405](https://pubchem.ncbi.nlm.nih.gov/compound/59444405)  [162113](https://pubchem.ncbi.nlm.nih.gov/compound/162113)  [59444401](https://pubchem.ncbi.nlm.nih.gov/compound/59444401)  [30607](https://pubchem.ncbi.nlm.nih.gov/compound/30607)  [71437560](https://pubchem.ncbi.nlm.nih.gov/compound/71437560)  [44139742](https://pubchem.ncbi.nlm.nih.gov/compound/44139742)  [44241652](https://pubchem.ncbi.nlm.nih.gov/compound/44241652) |
|  |  | Dehydrocannabifuran^7^  Cannabifuran^7^  cannabicitran^7^  cannabicoumaronone^7^  cannabiripsol^7^  Cannabimovone^7^  cannflavin C^7^  chrysoeriol^7^  6-prenylapigenin^7^  Docosanoic acid methyl ester^7^  acetyl stigmasterol^7^  α-spinasterol^7^  quebrachitol^7^  Isocannabispiradienone^7^  Cannabinol^7^  Tetrahydrocannabivarin^7^  tetrahydrocannabinolic acid^7^  cannabinolic acid^7^  cannabigerolic acid^7^ | [59444381](https://pubchem.ncbi.nlm.nih.gov/compound/59444381)  [9966466](https://pubchem.ncbi.nlm.nih.gov/compound/9966466)  [186149](https://pubchem.ncbi.nlm.nih.gov/compound/186149)  [625303](https://pubchem.ncbi.nlm.nih.gov/compound/625303)  [192007](https://pubchem.ncbi.nlm.nih.gov/compound/192007)  [46217279](https://pubchem.ncbi.nlm.nih.gov/compound/46217279)  [25141335](https://pubchem.ncbi.nlm.nih.gov/compound/25141335)  [5280666](https://pubchem.ncbi.nlm.nih.gov/compound/5280666)  [10382485](https://pubchem.ncbi.nlm.nih.gov/compound/10382485)  [13584](https://pubchem.ncbi.nlm.nih.gov/compound/13584)  [129671096](https://pubchem.ncbi.nlm.nih.gov/compound/129671096)  [5315190](https://pubchem.ncbi.nlm.nih.gov/compound/5315190)  [151108](https://pubchem.ncbi.nlm.nih.gov/compound/151108)  [101802953](https://pubchem.ncbi.nlm.nih.gov/compound/101802953)  [2543](https://pubchem.ncbi.nlm.nih.gov/compound/2543)  [93147](https://pubchem.ncbi.nlm.nih.gov/compound/93147)  [98523](https://pubchem.ncbi.nlm.nih.gov/compound/98523)  [3081990](https://pubchem.ncbi.nlm.nih.gov/compound/3081990)  [6449999](https://pubchem.ncbi.nlm.nih.gov/compound/6449999) |
|  |  | cannabigerolic acid monomethyl ether^8^  cannabigerovarinic acid^8^  cannabigerovarin^8^  cannabichromevarin^8^  cannabidiorcol^8^  cannabinol methylether^8^  cannabivarin^8^  cannabichromanon^8^  trigonelline^8^  muscarine^8^  neurine^8^  Piperidine^8^  Hordenine^8^  n-propylamine^8^  n-butylamine^8^  diethylamine^8^  pyrrolidine^8^  Cannabisativine^8^  Alanine^8^  galacturonic acid^8^  erythritol^8^  galactitol^8^  ribitol^8^ | [24739091](https://pubchem.ncbi.nlm.nih.gov/compound/24739091)  [59444383](https://pubchem.ncbi.nlm.nih.gov/compound/59444383)  [59444407](https://pubchem.ncbi.nlm.nih.gov/compound/59444407)  [6451726](https://pubchem.ncbi.nlm.nih.gov/compound/6451726)  [20586765](https://pubchem.ncbi.nlm.nih.gov/compound/20586765)  [628150](https://pubchem.ncbi.nlm.nih.gov/compound/628150)  [622545](https://pubchem.ncbi.nlm.nih.gov/compound/622545)  [25105340](https://pubchem.ncbi.nlm.nih.gov/compound/25105340)  [5570](https://pubchem.ncbi.nlm.nih.gov/compound/5570)  [9308](https://pubchem.ncbi.nlm.nih.gov/compound/9308)  [10042](https://pubchem.ncbi.nlm.nih.gov/compound/10042)  [8082](https://pubchem.ncbi.nlm.nih.gov/compound/8082)  [68313](https://pubchem.ncbi.nlm.nih.gov/compound/68313)  [7852](https://pubchem.ncbi.nlm.nih.gov/compound/7852)  [8007](https://pubchem.ncbi.nlm.nih.gov/compound/8007)  [8021](https://pubchem.ncbi.nlm.nih.gov/compound/8021)  [31268](https://pubchem.ncbi.nlm.nih.gov/compound/31268)  [442846](https://pubchem.ncbi.nlm.nih.gov/compound/442846)  [602](https://pubchem.ncbi.nlm.nih.gov/compound/602)  [439215](https://pubchem.ncbi.nlm.nih.gov/compound/439215)  [222285](https://pubchem.ncbi.nlm.nih.gov/compound/222285)  [11850](https://pubchem.ncbi.nlm.nih.gov/compound/11850)  [6912](https://pubchem.ncbi.nlm.nih.gov/compound/6912) |
|  |  | Malonic acid^8^  Phosphoric acid^8^  Pyroglutamic acid^8^  Quinic acid^8^  Behenic acid^8^  Sativic acid^8^  hexyl caproate^8^  campest-4-en-3-one^8^  camphenehydrate^8^  carvacrol^8^  carvone^8^  citronellol^8^  dihydrocarveyl acetate^8^  dihydrocarvone^8^  pinocarveol^8^  pinocarvone^8^  allo-aromadendrene^8^  longifolene^8^  nerolidol^8^  epi-beta-santalene^8^  vomifoliol^8^  dihydrovomifoliol^8^  cannabispiradienone^8^  beta-cannabispiranol^8^  cannabispirenone^8^  cannabispirone^8^  canniprene^8^  orientin^8^ | [867](https://pubchem.ncbi.nlm.nih.gov/compound/867)  [1004](https://pubchem.ncbi.nlm.nih.gov/compound/1004)  [7405](https://pubchem.ncbi.nlm.nih.gov/compound/7405)  [6508](https://pubchem.ncbi.nlm.nih.gov/compound/6508)  [633932](https://pubchem.ncbi.nlm.nih.gov/compound/633932)  [92800](https://pubchem.ncbi.nlm.nih.gov/compound/92800)  [22873](https://pubchem.ncbi.nlm.nih.gov/compound/22873)  [11988279](https://pubchem.ncbi.nlm.nih.gov/compound/11988279)  [22155067](https://pubchem.ncbi.nlm.nih.gov/compound/22155067)  [10364](https://pubchem.ncbi.nlm.nih.gov/compound/10364)  [7439](https://pubchem.ncbi.nlm.nih.gov/compound/7439)  [8842](https://pubchem.ncbi.nlm.nih.gov/compound/8842)  [30248](https://pubchem.ncbi.nlm.nih.gov/compound/30248)  [24473](https://pubchem.ncbi.nlm.nih.gov/compound/24473)  [102667](https://pubchem.ncbi.nlm.nih.gov/compound/102667)  [121719](https://pubchem.ncbi.nlm.nih.gov/compound/121719)  [42608158](https://pubchem.ncbi.nlm.nih.gov/compound/42608158)  [289151](https://pubchem.ncbi.nlm.nih.gov/compound/289151)  [5284507](https://pubchem.ncbi.nlm.nih.gov/compound/5284507)  [11106484](https://pubchem.ncbi.nlm.nih.gov/compound/11106484)  [5280462](https://pubchem.ncbi.nlm.nih.gov/compound/5280462)  [129317062](https://pubchem.ncbi.nlm.nih.gov/compound/129317062)  [90475437](https://pubchem.ncbi.nlm.nih.gov/compound/90475437)  [194174](https://pubchem.ncbi.nlm.nih.gov/compound/194174)  [10105874](https://pubchem.ncbi.nlm.nih.gov/compound/10105874)  [162936](https://pubchem.ncbi.nlm.nih.gov/compound/162936)  [53439651](https://pubchem.ncbi.nlm.nih.gov/compound/53439651)  [5281675](https://pubchem.ncbi.nlm.nih.gov/compound/5281675) |
| 46. | *Carthamus tinctorius*  (33) | Carthamin^9^  Safflor yellow A^9^  Safflor yellow B^9^  Saffloflavonesides A^9^  Saffloflavonesides B^9^  Safflomin A (Hydroxysafflor yellow A)^9^  Tinctormine^9^  Safflomin C^9^  Precarthamin^9^  Saffloquinoside A^9^  Saffloquinoside B^9^  Cartormin^9^  Anhydrosafflor yellow B^9^  Acacetin 7-O-alpha-L-rhamnopyranoside^9^  Luteolin 7-O-beta-D-glucopyranoside^9^  Acacetin^9^  Isorhamnetin^9^  Umbelliferone^9^  Daphnoretin^9^  N-feruloylserotonin^9^  N-(p-coumaroyl)serotonin^9^  Serotobenine^9^  N-feruloyltryptamine^9^  1-Tridecene-3,5,7,9,11-pentayne^9^  Sinapic acid^9^  Roseoside^9^  Methylsyringin^9^  Coniferyl alcohol^9^  Sinapyl alcohol^9^  Secoisolariciresinol^9^  Matairesinol^9^  Arctigenin^9^  Trachelogenin^9^ | [135565560](https://pubchem.ncbi.nlm.nih.gov/compound/135565560)  [71463725](https://pubchem.ncbi.nlm.nih.gov/compound/71463725)  [131751452](https://pubchem.ncbi.nlm.nih.gov/compound/131751452)  [101878345](https://pubchem.ncbi.nlm.nih.gov/compound/101878345)  [101878346](https://pubchem.ncbi.nlm.nih.gov/compound/101878346)  [6443665](https://pubchem.ncbi.nlm.nih.gov/compound/6443665)  [42607657](https://pubchem.ncbi.nlm.nih.gov/compound/42607657)  [42607658](https://pubchem.ncbi.nlm.nih.gov/compound/42607658)  [101928647](https://pubchem.ncbi.nlm.nih.gov/compound/101928647)  [45276863](https://pubchem.ncbi.nlm.nih.gov/compound/45276863)  [101501319](https://pubchem.ncbi.nlm.nih.gov/compound/101501319)  [131751684](https://pubchem.ncbi.nlm.nih.gov/compound/131751684)  [102240413](https://pubchem.ncbi.nlm.nih.gov/compound/102240413)  [44257896](https://pubchem.ncbi.nlm.nih.gov/compound/44257896)  [13093777](https://pubchem.ncbi.nlm.nih.gov/compound/13093777)  [5280442](https://pubchem.ncbi.nlm.nih.gov/compound/5280442)  [5281654](https://pubchem.ncbi.nlm.nih.gov/compound/5281654)  [5281426](https://pubchem.ncbi.nlm.nih.gov/compound/5281426)  [5281406](https://pubchem.ncbi.nlm.nih.gov/compound/5281406)  [5969616](https://pubchem.ncbi.nlm.nih.gov/compound/5969616)  [5458879](https://pubchem.ncbi.nlm.nih.gov/compound/5458879)  [11725426](https://pubchem.ncbi.nlm.nih.gov/compound/11725426)  [5458878](https://pubchem.ncbi.nlm.nih.gov/compound/5458878)  [441552](https://pubchem.ncbi.nlm.nih.gov/compound/441552)  [637775](https://pubchem.ncbi.nlm.nih.gov/compound/637775)  [9930064](https://pubchem.ncbi.nlm.nih.gov/compound/9930064)  [131752679](https://pubchem.ncbi.nlm.nih.gov/compound/131752679)  [1549095](https://pubchem.ncbi.nlm.nih.gov/compound/1549095)  [5280507](https://pubchem.ncbi.nlm.nih.gov/compound/5280507)  [65373](https://pubchem.ncbi.nlm.nih.gov/compound/65373)  [119205](https://pubchem.ncbi.nlm.nih.gov/compound/119205)  [64981](https://pubchem.ncbi.nlm.nih.gov/compound/64981)  [452855](https://pubchem.ncbi.nlm.nih.gov/compound/452855) |
| 47. | *Syzygium aromaticum*  (18) | Guaiacol^10^  Phloroglucinol^10^  Phenylacetic acid^10^  Gentisic acid^10^  Naringenin Chalcone^10^  Glycitein^10^  Delphinidin^10^  Malvidin^10^  Allo Ocimene^10^  Pinene-2-OL^10^  Linalyl Acetate^10^  Elemicin^10^  5-Hexene-2-one^11^  Guaiol^11^  Benzene-1-butylheptyl^11^  Nootkatin^11^  9,17-Octadecadienal^11^  Octadecanoic acid butyl ester^11^ | [460](https://pubchem.ncbi.nlm.nih.gov/compound/460)  [359](https://pubchem.ncbi.nlm.nih.gov/compound/359)  [999](https://pubchem.ncbi.nlm.nih.gov/compound/999)  [3469](https://pubchem.ncbi.nlm.nih.gov/compound/3469)  [5280960](https://pubchem.ncbi.nlm.nih.gov/compound/5280960)  [5317750](https://pubchem.ncbi.nlm.nih.gov/compound/5317750)  [68245](https://pubchem.ncbi.nlm.nih.gov/compound/68245)  [159287](https://pubchem.ncbi.nlm.nih.gov/compound/159287)  [5368821](https://pubchem.ncbi.nlm.nih.gov/compound/5368821)  [22013424](https://pubchem.ncbi.nlm.nih.gov/compound/22013424)  [8294](https://pubchem.ncbi.nlm.nih.gov/compound/8294)  [10248](https://pubchem.ncbi.nlm.nih.gov/compound/10248)  [7989](https://pubchem.ncbi.nlm.nih.gov/compound/7989)  [227829](https://pubchem.ncbi.nlm.nih.gov/compound/227829)  [20661](https://pubchem.ncbi.nlm.nih.gov/compound/20661)  [238797](https://pubchem.ncbi.nlm.nih.gov/compound/238797)  [6431297](https://pubchem.ncbi.nlm.nih.gov/compound/6431297)  [31278](https://pubchem.ncbi.nlm.nih.gov/compound/31278) |
| 48. | *Cornus officinalis*  (103) | Tellimagrandin II^12^  Cornusiin A^12^  Cornusiin B^12^  Cornusiin C^12^  Gemin D^12^  Isoterchebin^12^  Tellimagrandin I^12^  Cornusiin G^12^  Cornusiin D^12^  Zoomeric acid^12^  Nervonic acid^12^  Lignoceric acid^12^  Phthalic anhydride^12^  Trans-9-Octadecenoic acid^12^  3, 5-Bis (1, 1-dimethylethyl)phenol^12^  4-Methyloctanoic acid^12^  2-Dibenzofuransulfonic acid^12^  Morroniside^12^  Cornuside^12^  Loniceroside^12^  Kingiside^12^  Sweroside^12^  Secoxyloganin^12^  8-epikingiside^12^  Secologanoside^12^  Cornin (Verbenalin)^12^  Hastatoside^12^  Loganin^12^  Loganic acid^12^  7-O-methyl-morroniside^12^  7-O-ethyl-morroniside^12^  10-hydroxycornin^12^  10-hydroxyhastatoside^12^  Secologanin^12^  Arjunglucoside Ⅱ^12^  Linalyl propionate^12^  6, 10-Dimethyl-2-undecanone^12^  p-Menth-1-ene-7, 8-diol^12^  Cubebol^12^  Tachioside^12^ | [151590](https://pubchem.ncbi.nlm.nih.gov/compound/151590)  [16129730](https://pubchem.ncbi.nlm.nih.gov/compound/16129730)  [16131156](https://pubchem.ncbi.nlm.nih.gov/compound/16131156)  [16132407](https://pubchem.ncbi.nlm.nih.gov/compound/16132407)  [471119](https://pubchem.ncbi.nlm.nih.gov/compound/471119)  [442685](https://pubchem.ncbi.nlm.nih.gov/compound/442685)  [442690](https://pubchem.ncbi.nlm.nih.gov/compound/442690)  [102061533](https://pubchem.ncbi.nlm.nih.gov/compound/102061533)  [101826455](https://pubchem.ncbi.nlm.nih.gov/compound/101826455)  [445638](https://pubchem.ncbi.nlm.nih.gov/compound/445638)  [5281120](https://pubchem.ncbi.nlm.nih.gov/compound/5281120)  [11197](https://pubchem.ncbi.nlm.nih.gov/compound/11197)  [6811](https://pubchem.ncbi.nlm.nih.gov/compound/6811)  [637517](https://pubchem.ncbi.nlm.nih.gov/compound/637517)  [70825](https://pubchem.ncbi.nlm.nih.gov/compound/70825)  [62089](https://pubchem.ncbi.nlm.nih.gov/compound/62089)  [522803](https://pubchem.ncbi.nlm.nih.gov/compound/522803)  [11228693](https://pubchem.ncbi.nlm.nih.gov/compound/11228693)  [11228694](https://pubchem.ncbi.nlm.nih.gov/compound/11228694)  [179500](https://pubchem.ncbi.nlm.nih.gov/compound/179500)  [12304884](https://pubchem.ncbi.nlm.nih.gov/compound/12304884)  [161036](https://pubchem.ncbi.nlm.nih.gov/compound/161036)  [162868](https://pubchem.ncbi.nlm.nih.gov/compound/162868)  [12304886](https://pubchem.ncbi.nlm.nih.gov/compound/12304886)  [14136854](https://pubchem.ncbi.nlm.nih.gov/compound/14136854)  [73467](https://pubchem.ncbi.nlm.nih.gov/compound/73467)  [92043450](https://pubchem.ncbi.nlm.nih.gov/compound/92043450)  [87691](https://pubchem.ncbi.nlm.nih.gov/compound/87691)  [89640](https://pubchem.ncbi.nlm.nih.gov/compound/89640)  [127258930](https://pubchem.ncbi.nlm.nih.gov/compound/127258930)  [74399180](https://pubchem.ncbi.nlm.nih.gov/compound/74399180)  [101637374](https://pubchem.ncbi.nlm.nih.gov/compound/101637374)  [100952769](https://pubchem.ncbi.nlm.nih.gov/compound/100952769)  [161276](https://pubchem.ncbi.nlm.nih.gov/compound/161276)  [52951052](https://pubchem.ncbi.nlm.nih.gov/compound/52951052)  [61098](https://pubchem.ncbi.nlm.nih.gov/compound/61098)  [95495](https://pubchem.ncbi.nlm.nih.gov/compound/95495)  [110662](https://pubchem.ncbi.nlm.nih.gov/compound/110662)  [11276107](https://pubchem.ncbi.nlm.nih.gov/compound/11276107)  [11962143](https://pubchem.ncbi.nlm.nih.gov/compound/11962143) |
|  |  | Glycidol^13^  3-Vinyl-1-cyclobutene^13^  1,3-Cyclohexadiene^13^  Methacrolein^13^  2-Vinylfuran^13^  1-Ethoxypropan-2-yl acetate^13^  Pyridine^13^  Pyrrole^13^  3-Pyrrolidinol^13^  3-Furaldehyde^13^  3-Furanmethanol^13^  Propargylamine^13^  Furfural^13^  2-Furanmethanol^13^  Cis-bicyclo[4.2.0]octane^13^  4-Cyclopentene-1,3-dione^13^  1-Nonene^13^  Pentanoic acid^13^  2-Formylhistamine^13^  2(5H)-Furanone^13^  2-Cyclohexen-1-one^13^  1-Methylpyrazol-3-amine^13^  2H-Pyran-2-one^13^  1H-Pyrrole-2-carboxaldehyde^13^  4-Methyl-5H-furan-2-one^13^  p-Cresol^13^  2-Pentyne^13^  Maltol^13^  Benzyl nitrile^13^  Dehydromevalonic lactone^13^  E-7-Tetradecenol^13^  Creosol^13^  Catechol^13^  5-Hydroxymethylfurfural^13^  2-Coumaranone^13^  4,6-Dioxadodecane^13^  1-Tridecene^13^  Kessane^13^  Oxaceprol^13^  2-Methoxy-4-vinylphenol^13^ | [11164](https://pubchem.ncbi.nlm.nih.gov/compound/11164)  [556371](https://pubchem.ncbi.nlm.nih.gov/compound/556371)  [11605](https://pubchem.ncbi.nlm.nih.gov/compound/11605)  [6562](https://pubchem.ncbi.nlm.nih.gov/compound/6562)  [73881](https://pubchem.ncbi.nlm.nih.gov/compound/73881)  [171378](https://pubchem.ncbi.nlm.nih.gov/compound/171378)  [1049](https://pubchem.ncbi.nlm.nih.gov/compound/1049)  [8027](https://pubchem.ncbi.nlm.nih.gov/compound/8027)  [98210](https://pubchem.ncbi.nlm.nih.gov/compound/98210)  [10351](https://pubchem.ncbi.nlm.nih.gov/compound/10351)  [20449](https://pubchem.ncbi.nlm.nih.gov/compound/20449)  [239041](https://pubchem.ncbi.nlm.nih.gov/compound/239041)  [7362](https://pubchem.ncbi.nlm.nih.gov/compound/7362)  [7361](https://pubchem.ncbi.nlm.nih.gov/compound/7361)  [643590](https://pubchem.ncbi.nlm.nih.gov/compound/643590)  [70258](https://pubchem.ncbi.nlm.nih.gov/compound/70258)  [31285](https://pubchem.ncbi.nlm.nih.gov/compound/31285)  [7991](https://pubchem.ncbi.nlm.nih.gov/compound/7991)  [541600](https://pubchem.ncbi.nlm.nih.gov/compound/541600)  [10341](https://pubchem.ncbi.nlm.nih.gov/compound/10341)  [13594](https://pubchem.ncbi.nlm.nih.gov/compound/13594)  [137254](https://pubchem.ncbi.nlm.nih.gov/compound/137254)  [68154](https://pubchem.ncbi.nlm.nih.gov/compound/68154)  [13854](https://pubchem.ncbi.nlm.nih.gov/compound/13854)  [145832](https://pubchem.ncbi.nlm.nih.gov/compound/145832)  [2879](https://pubchem.ncbi.nlm.nih.gov/compound/2879)  [12310](https://pubchem.ncbi.nlm.nih.gov/compound/12310)  [8369](https://pubchem.ncbi.nlm.nih.gov/compound/8369)  [8794](https://pubchem.ncbi.nlm.nih.gov/compound/8794)  [557445](https://pubchem.ncbi.nlm.nih.gov/compound/557445)  [5362726](https://pubchem.ncbi.nlm.nih.gov/compound/5362726)  [7144](https://pubchem.ncbi.nlm.nih.gov/compound/7144)  [289](https://pubchem.ncbi.nlm.nih.gov/compound/289)  [237332](https://pubchem.ncbi.nlm.nih.gov/compound/237332)  [68382](https://pubchem.ncbi.nlm.nih.gov/compound/68382)  [57645665](https://pubchem.ncbi.nlm.nih.gov/compound/57645665)  [17095](https://pubchem.ncbi.nlm.nih.gov/compound/17095)  [11310616](https://pubchem.ncbi.nlm.nih.gov/compound/11310616)  [65784](https://pubchem.ncbi.nlm.nih.gov/compound/65784)  [332](https://pubchem.ncbi.nlm.nih.gov/compound/332) |
|  |  | 1,2,3-Benzenetriol^13^  1,2,4-Benzenetriol^13^  3,4-Dimethyl-o-phenylenediamine^13^  3,5-Dimethoxy-4-hydroxytoluene^13^  4-Methoxycinnamaldehyde^13^  D-Allose^13^  1-Pentadecene^13^  Pentadecane^13^  Cyclotetradecane^13^  Butyrovanillone^13^  Tridecanoic acid^13^  1-Heptadecene^13^  9-Hexadecenoic acid^13^  5-Amino-2-thiocyanoacetophenone^13^  Cyanoacetic acid, dodecyl ester^13^  1,5-Dodecadiene^13^  cis-1-Chloro-9-octadecene^13^  1-Nonadecene^13^  1-Octadecene^13^  Methyl 13-methyl-eicosanoate^13^  Tricosane^13^  Oxacycloheptadecan-2-one^13^  1-Docosene^13^ | [1057](https://pubchem.ncbi.nlm.nih.gov/compound/1057)  [10787](https://pubchem.ncbi.nlm.nih.gov/compound/10787)  [521077](https://pubchem.ncbi.nlm.nih.gov/compound/521077)  [240925](https://pubchem.ncbi.nlm.nih.gov/compound/240925)  [641294](https://pubchem.ncbi.nlm.nih.gov/compound/641294)  [439507](https://pubchem.ncbi.nlm.nih.gov/compound/439507)  [25913](https://pubchem.ncbi.nlm.nih.gov/compound/25913)  [12391](https://pubchem.ncbi.nlm.nih.gov/compound/12391)  [67524](https://pubchem.ncbi.nlm.nih.gov/compound/67524)  [14035098](https://pubchem.ncbi.nlm.nih.gov/compound/14035098)  [12530](https://pubchem.ncbi.nlm.nih.gov/compound/12530)  [23217](https://pubchem.ncbi.nlm.nih.gov/compound/23217)  [5282745](https://pubchem.ncbi.nlm.nih.gov/compound/5282745)  [284762](https://pubchem.ncbi.nlm.nih.gov/compound/284762)  [12606407](https://pubchem.ncbi.nlm.nih.gov/compound/12606407)  [5363382](https://pubchem.ncbi.nlm.nih.gov/compound/5363382)  [5367784](https://pubchem.ncbi.nlm.nih.gov/compound/5367784)  [29075](https://pubchem.ncbi.nlm.nih.gov/compound/29075)  [8217](https://pubchem.ncbi.nlm.nih.gov/compound/8217)  [85964992](https://pubchem.ncbi.nlm.nih.gov/compound/85964992)  [12534](https://pubchem.ncbi.nlm.nih.gov/compound/12534)  [7984](https://pubchem.ncbi.nlm.nih.gov/compound/7984)  [74138](https://pubchem.ncbi.nlm.nih.gov/compound/74138) |
| 49. | *Eucommia ulmoides*  (57) | (−)-olivil^14^  Lariciresinol^14^  Balanophonin^14^  vladinol D^14^  (+)-cyclo-olivil^14^  (+)-medioresinol^14^  (+)-pinoresinol^14^  (+)-syringaresinol^14^  (+)-epipinoresinol^14^  Arctiin^14^  citrusin B^14^  threo-dihydroxydehydrodiconiferyl alcohol^14^  hedyotol C^14^  genipin^14^  aucubin^14^  geniposide^14^  geniposidic acid^14^  asperulosidic acid^14^  asperulosidic acid ethyl ester^14^  Ajugoside^14^  Reptoside^14^  eucommiol^14^  eucommioside II^14^  1-deoxyeucommiol^14^  Epieucommiol^14^  ulmoidoside A^14^  ulmoidoside B^14^ | [5273570](https://pubchem.ncbi.nlm.nih.gov/compound/5273570)  [332427](https://pubchem.ncbi.nlm.nih.gov/compound/332427)  [23252258](https://pubchem.ncbi.nlm.nih.gov/compound/23252258)  [70698172](https://pubchem.ncbi.nlm.nih.gov/compound/70698172)  [5316262](https://pubchem.ncbi.nlm.nih.gov/compound/5316262)  [181681](https://pubchem.ncbi.nlm.nih.gov/compound/181681)  [73399](https://pubchem.ncbi.nlm.nih.gov/compound/73399)  [443023](https://pubchem.ncbi.nlm.nih.gov/compound/443023)  [637584](https://pubchem.ncbi.nlm.nih.gov/compound/637584)  [100528](https://pubchem.ncbi.nlm.nih.gov/compound/100528)  [131752580](https://pubchem.ncbi.nlm.nih.gov/compound/131752580)  [5317204](https://pubchem.ncbi.nlm.nih.gov/compound/5317204)  [21636185](https://pubchem.ncbi.nlm.nih.gov/compound/21636185)  [442424](https://pubchem.ncbi.nlm.nih.gov/compound/442424)  [91458](https://pubchem.ncbi.nlm.nih.gov/compound/91458)  [107848](https://pubchem.ncbi.nlm.nih.gov/compound/107848)  [443354](https://pubchem.ncbi.nlm.nih.gov/compound/443354)  [11968867](https://pubchem.ncbi.nlm.nih.gov/compound/11968867)  [21580986](https://pubchem.ncbi.nlm.nih.gov/compound/21580986)  [9865184](https://pubchem.ncbi.nlm.nih.gov/compound/9865184)  [44584096](https://pubchem.ncbi.nlm.nih.gov/compound/44584096)  [154373](https://pubchem.ncbi.nlm.nih.gov/compound/154373)  [6325167](https://pubchem.ncbi.nlm.nih.gov/compound/6325167)  [5316559](https://pubchem.ncbi.nlm.nih.gov/compound/5316559)  [101714787](https://pubchem.ncbi.nlm.nih.gov/compound/101714787)  [14540382](https://pubchem.ncbi.nlm.nih.gov/compound/14540382)  [14540384](https://pubchem.ncbi.nlm.nih.gov/compound/14540384) |
|  |  | asperuloside^14^  ulmoside^14^  eucomoside A^14^  eucomoside B^14^  eucomoside C^14^  daphylloside^14^  scandoside methyl ester^14^  loganin^14^  7-epi-loganin^14^  artselaenin C^14^  harpagide^14^  catalpol^14^  avicularin^14^  oroxylin A^14^  licochalcone A^14^  thunberginol C^14^  C-veratroylglycol^14^  alternariol^14^  salicifoliol^14^  eucophenoside^14^  Uvaol^14^  eleganoside A^14^  borreriagenin^14^ | [84298](https://pubchem.ncbi.nlm.nih.gov/compound/84298)  [11968295](https://pubchem.ncbi.nlm.nih.gov/compound/11968295)  [102382672](https://pubchem.ncbi.nlm.nih.gov/compound/102382672)  [23624546](https://pubchem.ncbi.nlm.nih.gov/compound/23624546)  [23625086](https://pubchem.ncbi.nlm.nih.gov/compound/23625086)  [21602024](https://pubchem.ncbi.nlm.nih.gov/compound/21602024)  [442433](https://pubchem.ncbi.nlm.nih.gov/compound/442433)  [87691](https://pubchem.ncbi.nlm.nih.gov/compound/87691)  [10548420](https://pubchem.ncbi.nlm.nih.gov/compound/10548420)  [100930978](https://pubchem.ncbi.nlm.nih.gov/compound/100930978)  [10044294](https://pubchem.ncbi.nlm.nih.gov/compound/10044294)  [91520](https://pubchem.ncbi.nlm.nih.gov/compound/91520)  [5490064](https://pubchem.ncbi.nlm.nih.gov/compound/5490064)  [5320315](https://pubchem.ncbi.nlm.nih.gov/compound/5320315)  [5318998](https://pubchem.ncbi.nlm.nih.gov/compound/5318998)  [10333412](https://pubchem.ncbi.nlm.nih.gov/compound/10333412)  [15765124](https://pubchem.ncbi.nlm.nih.gov/compound/15765124)  [5359485](https://pubchem.ncbi.nlm.nih.gov/compound/5359485)  [10955962](https://pubchem.ncbi.nlm.nih.gov/compound/10955962)  [102044905](https://pubchem.ncbi.nlm.nih.gov/compound/102044905)  [92802](https://pubchem.ncbi.nlm.nih.gov/compound/92802)  [101085911](https://pubchem.ncbi.nlm.nih.gov/compound/101085911)  [44583980](https://pubchem.ncbi.nlm.nih.gov/compound/44583980) |
|  |  | 3-O-acetylerythrodiol^15^  aplyolide D^15^  Umbelliferone^15^  Eriodictyol^15^  Homoeriodictyol^15^  Aromadendrol^15^  cyclo(Pro-Pro)^15^ | [118796402](https://pubchem.ncbi.nlm.nih.gov/compound/118796402)  [10708956](https://pubchem.ncbi.nlm.nih.gov/compound/10708956)  [5281426](https://pubchem.ncbi.nlm.nih.gov/compound/5281426)  [440735](https://pubchem.ncbi.nlm.nih.gov/compound/440735)  [73635](https://pubchem.ncbi.nlm.nih.gov/compound/73635)  [122850](https://pubchem.ncbi.nlm.nih.gov/compound/122850)  [529063](https://pubchem.ncbi.nlm.nih.gov/compound/529063) |
| 50. | *Foeniculum vulgare*  (57) | Anisketone^16^  Apiol^16^  4-Carene^16^  3-Carene^16^  Cathine^16^  2-Propyn-1-ol^16^  2,6-Dimethyl-2,4,6-octatriene^16^  Sabinene hydrate^16^  Fenchyl acetate^16^  Dicyclopropyl carbinol^16^  Estragole^16^  *trans*-p-2,8-menthadien-1-ol^16^  1,4-Dimethoxybenzene^16^  *trans*-Anethole^16^  Allantoic acid^16^  1-Undecanol^16^  Benzothiazole^16^  1,6-Hexanediol^16^  Mephenesin^16^  4-Fluorohistamine^16^  1-(3-Methoxyphenyl)-1-propanone^16^ | [31231](https://pubchem.ncbi.nlm.nih.gov/compound/31231)  [10659](https://pubchem.ncbi.nlm.nih.gov/compound/10659)  [530422](https://pubchem.ncbi.nlm.nih.gov/compound/530422)  [26049](https://pubchem.ncbi.nlm.nih.gov/compound/26049)  [441457](https://pubchem.ncbi.nlm.nih.gov/compound/441457)  [7859](https://pubchem.ncbi.nlm.nih.gov/compound/7859)  [5368821](https://pubchem.ncbi.nlm.nih.gov/compound/5368821)  [62367](https://pubchem.ncbi.nlm.nih.gov/compound/62367)  [107217](https://pubchem.ncbi.nlm.nih.gov/compound/107217)  [84336](https://pubchem.ncbi.nlm.nih.gov/compound/84336)  [8815](https://pubchem.ncbi.nlm.nih.gov/compound/8815)  [155626](https://pubchem.ncbi.nlm.nih.gov/compound/155626)  [9016](https://pubchem.ncbi.nlm.nih.gov/compound/9016)  [637563](https://pubchem.ncbi.nlm.nih.gov/compound/637563)  [203](https://pubchem.ncbi.nlm.nih.gov/compound/203)  [8184](https://pubchem.ncbi.nlm.nih.gov/compound/8184)  [7222](https://pubchem.ncbi.nlm.nih.gov/compound/7222)  [12374](https://pubchem.ncbi.nlm.nih.gov/compound/12374)  [4059](https://pubchem.ncbi.nlm.nih.gov/compound/4059)  [541569](https://pubchem.ncbi.nlm.nih.gov/compound/541569)  [584765](https://pubchem.ncbi.nlm.nih.gov/compound/584765) |
|  |  | 1,5 dicaffeoylquinic acid^17^  Hesperidin^17^  3- caffeoylquinic acid^17^  4- caffeoylquinic acid^17^  imperatorin^17^  psoralen^17^  bergapten^17^  xanthotoxin^17^  isopimpinellin^17^  Cuminal^17^  cis-Anethole^17^  p-Anisaldehyde^17^  2-methyl-3-oxoestran- 17-yl acetate^17^  Vetivenene^17^  Anthracene^17^  2-Methoxy-4-ethylphenol^17^  1,2-Dimethoxy-4-methylbenzene^17^  Cedran-9-one^17^  2,2-dimethyl-3-phenylpropanoate^17^  3,3,6-Trimethyl-1-indanone^17^  o-Benzenedicarboxylic acid^17^  3,4-Dimethyl-1,5-cyclooctadiene^17^  capric acid^17^  undecanoic acid^17^  pentadecadienoic acid^17^  erucic acid^17^  n-Hexacosane^17^  n-Heptacosane^17^  n-Octacosane^17^  n-Nonacosane^17^  n-Triacontane^17^  n-Dotriacontane^17^  n-Tetratriacontane^17^  n-Hexatriacontane^17^  n-Octatriacontane^17^  n-Tetracontane^17^ | [122685](https://pubchem.ncbi.nlm.nih.gov/compound/122685)  [10621](https://pubchem.ncbi.nlm.nih.gov/compound/10621)  [102111217](https://pubchem.ncbi.nlm.nih.gov/compound/102111217)  [9798666](https://pubchem.ncbi.nlm.nih.gov/compound/9798666)  [10212](https://pubchem.ncbi.nlm.nih.gov/compound/10212)  [6199](https://pubchem.ncbi.nlm.nih.gov/compound/6199)  [2355](https://pubchem.ncbi.nlm.nih.gov/compound/2355)  [4114](https://pubchem.ncbi.nlm.nih.gov/compound/4114)  [68079](https://pubchem.ncbi.nlm.nih.gov/compound/68079)  [326](https://pubchem.ncbi.nlm.nih.gov/compound/326)  [1549040](https://pubchem.ncbi.nlm.nih.gov/compound/1549040)  [31244](https://pubchem.ncbi.nlm.nih.gov/compound/31244)  [22212600](https://pubchem.ncbi.nlm.nih.gov/compound/22212600)  [529892](https://pubchem.ncbi.nlm.nih.gov/compound/529892)  [8418](https://pubchem.ncbi.nlm.nih.gov/compound/8418)  [62465](https://pubchem.ncbi.nlm.nih.gov/compound/62465)  [68126](https://pubchem.ncbi.nlm.nih.gov/compound/68126)  [21723990](https://pubchem.ncbi.nlm.nih.gov/compound/21723990)  [6947002](https://pubchem.ncbi.nlm.nih.gov/compound/6947002)  [594538](https://pubchem.ncbi.nlm.nih.gov/compound/594538)  [1017](https://pubchem.ncbi.nlm.nih.gov/compound/1017)  [5365753](https://pubchem.ncbi.nlm.nih.gov/compound/5365753)  [2969](https://pubchem.ncbi.nlm.nih.gov/compound/2969)  [8180](https://pubchem.ncbi.nlm.nih.gov/compound/8180)  [19047235](https://pubchem.ncbi.nlm.nih.gov/compound/19047235)  [5281116](https://pubchem.ncbi.nlm.nih.gov/compound/5281116)  [12407](https://pubchem.ncbi.nlm.nih.gov/compound/12407)  [11636](https://pubchem.ncbi.nlm.nih.gov/compound/11636)  [12408](https://pubchem.ncbi.nlm.nih.gov/compound/12408)  [12409](https://pubchem.ncbi.nlm.nih.gov/compound/12409)  [12535](https://pubchem.ncbi.nlm.nih.gov/compound/12535)  [11008](https://pubchem.ncbi.nlm.nih.gov/compound/11008)  [26519](https://pubchem.ncbi.nlm.nih.gov/compound/26519)  [12412](https://pubchem.ncbi.nlm.nih.gov/compound/12412)  [23599](https://pubchem.ncbi.nlm.nih.gov/compound/23599)  [20149](https://pubchem.ncbi.nlm.nih.gov/compound/20149) |

**References:**

1. Wang, Z. *et al.* Phytochemistry, pharmacology, quality control and future research of Forsythia suspensa (Thunb.) Vahl: A review. *J. Ethnopharmacol.* **210**, 318–339 (2018).

2. Shu, Z. *et al.* Alisma orientale: Ethnopharmacology, Phytochemistry and Pharmacology of an Important Traditional Chinese Medicine. *Am. J. Chin. Med.* **44**, 227–251 (2016).

3. Choi, J. W., Kim, K. H., Lee, I. K., Choi, S. U. & Lee, K. R. Phytochemical constituents of Amomum xanthioides. *Nat. Prod. Sci.* **15**, 44–49 (2009).

4. Kim, K. H., Choi, J. W., Choi, S. U. & Lee, K. R. Cytotoxic sesquiterpenoid from the seeds of Amomum xanthioides. *Nat. Prod. Sci.* **17**, 10–13 (2011).

5. Thinh, B. B., Doudkin, R. V. & Thanh, V. Q. Chemical composition of essential oil of amomum xanthioides wall. Ex baker from Northern Vietnam. *Biointerface Res. Appl. Chem.* **11**, 12275–12284 (2021).

6. Peng, W. *et al.* Areca catechu L. (Arecaceae): A review of its traditional uses, botany, phytochemistry, pharmacology and toxicology. *J. Ethnopharmacol.* **164**, 340–356 (2015).

7. Saroya, A. S. *The Phytocannabinoids*. *Contemporary Phytomedicines* (2017). doi:10.1201/9781315367071-34.

8. C. E. Turner, M.A. Ellsohly, E. G. B. CONSTITUESTS OF CAS-TABIS SATIVA L. XT‘II. Ai REVIEIT OF THE XXTURXL CONSTITUENTS. *J. Nat. Prod.* (1980).

9. Zhang, L. Le *et al.* Phytochemistry and Pharmacology of Carthamus tinctorius L. *Am. J. Chin. Med.* **44**, 197–226 (2016).

10. Jimoh, S. O., Arowolo, L. A. & Alabi, K. A. Phytochemical Screening and Antimicrobial Evaluation of Syzygium aromaticum Extract and Essential oil. *Int. J. Curr. Microbiol. Appl. Sci.* **6**, 4557–4567 (2017).

11. Nassar, M. I. *et al.* Chemical constituents of clove (Syzygium aromaticum , Fam . Myrtaceae) and their antioxidant activity. *Rev. Latinoam. Quim.* **35**, 47–57 (2007).

12. Huang, J. *et al.* Ethnopharmacology, phytochemistry, and pharmacology of Cornus officinalis Sieb. et Zucc. *J. Ethnopharmacol.* **213**, 280–301 (2018).

13. Yue, X. *et al.* Molecules and functions of Cornus officinalis bark volatiles. *Emirates J. Food Agric.* **30**, 828–838 (2018).

14. Wang, C. Y., Tang, L., He, J. W., Li, J. & Wang, Y. Z. Ethnobotany, Phytochemistry and Pharmacological Properties of Eucommia ulmoides: A Review. *Am. J. Chin. Med.* **47**, 259–300 (2019).

15. Li, R. *et al.* Phytochemical constituents, chemotaxonomic significance and anti-arthritic effect of Eucommia ulmoides Oliver staminate flowers. *Nat. Prod. Res.* **0**, 1–5 (2020).

16. Badgujar, S. B., Patel, V. V. & Bandivdekar, A. H. Foeniculum vulgare Mill: A review of its botany, phytochemistry, pharmacology, contemporary application, and toxicology. *Biomed Res. Int.* **2014**, (2014).

17. Majid, A. The Chemical Constituents and Pharmacological Effects of Nigella sativa. *J. Biosci. Appl. Res.* **4**, 389–400 (2018).
