# Supplementary material for: An in silico approach to develop potential therapies against Middle East Respiratory Syndrome Coronavirus (MERS-CoV)
Source: Heliyon. 2024 Feb 9;10(4):e25837. doi: 10.1016/j.heliyon.2024.e25837 (PMC10877303; doi:10.1016/j.heliyon.2024.e25837)
Supplement: Multimedia component 6 [file mmc6.docx]

| **Compound CID** | **Compound Name** | **Docking Score**  **(kcal/mol)** |
| --- | --- | --- |
| CID_156766 | Kihadanin B | -9.8 |
| CID_5281694 | Robustaflavone | -9.4 |
| CID_14335962 | 3-beta-O-(trans-p-Coumaroyl)maslinic acid | -9.2 |
| CID_102081298 | Sprengerinin A | -9.1 |
| CID_10032468 | Actein | -9.1 |
| CID_21668683 | 23-EPI-26-Deoxyactein | -9.1 |
| CID_91439 | Smilagenin | -9.1 |
| CID_71767755 | Timosaponin A1 | -9.1 |
| CID_99474 | Diosgenin | -9.1 |
| CID_9847547 | 16,23-Oxidoalisol B | -9.0 |
| CID_132492418 | Astramembrannin II | -9.0 |
| CID_92095 | Sarsasapogenin | -9.0 |
| CID_21676348 | Ephedrannin A | -9.0 |
| CID_25051177 | Ephedrannin B | -9.0 |
| CID_5281600 | Amentoflavone | -8.9 |
| CID_12305177 | Arborinol | -8.9 |
| CID_99516 | Tigogenin | -8.9 |
| CID_442431 | Narirutin | -8.9 |
| CID_12314056 | Pennogenin | -8.9 |
| CID_14831162 | Chiratenol | -8.8 |
| CID_25233029 | Panaxadione | -8.8 |
| CID_181096 | Isobauerenyl acetate | -8.8 |
| CID_5321255 | Sennidin C | -8.7 |
| CID_15558620 | Alisol B | -8.7 |
| CID_101403595 | Platycoside M1 | -8.7 |

**Supplementary Table 1:** The docking interactions energy of the best 25 compounds in ‘Autodock vina’ docking program.

**Supplementary Table 2:** Re-docking interactions energy of the best 25 compounds in the 'Autodock Vina' docking program.

| **Compound CID** | **Compound Name** | **Docking Score**  **(kcal/mol)** |
| --- | --- | --- |
| CID_156766 | Kihadanin B | -9.8 |
| CID_5281694 | Robustaflavone | -9.4 |
| CID_14335962 | 3-beta-O-(trans-p-Coumaroyl)maslinic acid | -9.2 |
| CID_10032468 | Actein | -9.0 |
| CID_21668683 | 23-EPI-26-Deoxyactein | -9.0 |
| CID_21676348 | Ephedrannin A | -9.0 |
| CID_91439 | Smilagenin | -9.0 |
| CID_71767755 | Timosaponin A1 | -9.0 |
| CID_9847547 | 16,23-Oxidoalisol B | -9.0 |
| CID_99474 | Diosgenin | -9.0 |
| CID_132492418 | Astramembrannin II | -9.0 |
| CID_25051177 | Ephedrannin B | -9.0 |
| CID_92095 | Sarsasapogenin | -8.9 |
| CID_99516 | Tigogenin | -8.9 |
| CID_12314056 | Pennogenin | -8.9 |
| CID_181096 | Isobauerenyl acetate | -8.8 |
| CID_14831162 | Chiratenol | -8.8 |
| CID_12305177 | Arborinol | -8.8 |
| CID_101403595 | Platycoside M1 | -8.8 |
| CID_442431 | Narirutin | -8.8 |
| CID_102081298 | Sprengerinin A | -8.8 |
| CID_25233029 | Panaxadione | -8.7 |
| CID_15558620 | Alisol B | -8.6 |
| CID_5281600 | Amentoflavone | -8.5 |
| CID_5321255 | Sennidin C | -8.4 |

**Supplementary Table 3:** The docking interactions energy of the top ten compounds.

| **Compound CID** | **Compound Name** | **Docking Score**  **(kcal/mol)** |
| --- | --- | --- |
| CID_156766 | Kihadanin B | -9.8 |
| CID_5281694 | Robustaflavone | -9.4 |
| CID_14335962 | 3-beta-O-(trans-p-Coumaroyl)maslinic acid | -9.2 |
| CID_10032468 | Actein | -9.0 |
| CID_21668683 | 23-EPI-26-Deoxyactein | -9.0 |
| CID_21676348 | Ephedrannin A | -9.0 |
| CID_91439 | Smilagenin | -9.0 |
| CID_71767755 | Timosaponin A1 | -9.0 |
| CID_9847547 | 16,23-Oxidoalisol B | -9.0 |
| CID_99474 | Diosgenin | -9.0 |
